# A revision of the Neotropical species of *Bolitogyrus* Chevrolat, a geographically disjunct lineage of Staphylinini (Coleoptera, Staphylinidae)

**DOI:** 10.3897/zookeys.423.7536

**Published:** 2014-07-07

**Authors:** Adam J. Brunke, Alexey Solodovnikov

**Affiliations:** 1 Biosystematics, Natural History Museum of Denmark, University of Copenhagen, Universitetsparken 15, Copenhagen 2100, Denmark

**Keywords:** Staphylininae, Staphylinini, Neotropical, disjunct distribution, rSEM

## Abstract

The Neotropical species of the rarely collected genus *Bolitogyrus* (Coleoptera: Staphylinidae: Staphylininae: Staphylinini) are revised. The genus exhibits an uncommon, disjunct distribution between the Neotropical and Oriental Regions and is of unknown phylogenetic position within Staphylinini. Morphological evolution remarkable for Staphylinini was discovered within *Bolitogyrus*, including sexually dimorphic modifications of the pronotum that may be involved in male competition for females. rSEM interactive animations were used to establish morphological species boundaries between two highly variable species and are provided to illustrate diagnostic characters of the genitalia in unconventional views. The genus is redescribed based on the world fauna and twenty-eight Neotropical species are considered valid. Of these, nineteen are described as new to science: *Bolitogyrus ashei* **sp. n.**; *B. apicofasciatus* **sp. n.**; *B. brevistellus* **sp. n.**; *B. bufo* **sp. n.**; *B. cheungi* **sp. n.**; *B. cornutus* **sp. n.**; *B. divisus* **sp. n.**; *B. falini* **sp. n.**; *B. gracilis* **sp. n.**; *B. inexspectatus* **sp. n.**; *B. longistellus* **sp. n.**; *B. marquezi* **sp. n.**; *B. newtoni* **sp. n.**; *B. pseudotortifolius* **sp. n.**; *B. pulchrus* **sp. n.**; *B. silex* **sp. n.**; *B. thomasi* **sp. n.**; *B. tortifolius* **sp. n.**; and *B. viridescens* **sp. n.** *Bolitogyrus sallei* (Kraatz), **stat. r.** is removed from synonymy with *B. buphthalmus* (Erichson) and the following new synonyms are proposed: *Cyrtothorax cyanescens* Sharp, 1884, **syn. n.** = *Quedius buphthalmus* Erichson, 1840; *C. nevermanni* Scheerpeltz, 1974, **syn. n.** = *C. costaricensis* Wendeler, 1927. A summary of all available bionomic and distributional data, as well as an illustrated identification key to and diagnoses of all Neotropical species are provided.

## Introduction

*Bolitogyrus* Chevrolat (Staphylinidae: Staphylininae: Staphylinini) is a rarely encountered genus of rove beetles that exhibits a widely disjunct distribution in the northern Neotropical and Oriental regions. This distribution pattern is uncommonly reported in insects but is found elsewhere in the rove beetle tribe Staphylinini between sister genera *Alesiella* Brunke and Solodovnikov (Oriental) and *Quediomacrus* Sharp (northern Neotropical), and within the genus *Misantlius* Sharp. The disjunction between *Alesiella* and *Quediomacrus* was attributed to vicariance following the Early Eocene climatic optimum when subtropical forests were more widespread, allowing for broad ‘boreotropical’ distributions across land bridges in the Atlantic and Pacific oceans (Brunke and Solodovnikov 2013). In contrast to the above taxa, which are very species-poor on one or both sides of the Pacific Ocean, the known diversity of *Bolitogyrus* is moderate in both the Neotropical and Oriental regions. Thus, *Bolitogyrus* is a focal taxon with great potential to facilitate more detailed and rigorous investigations of the timing of, and the environmental factors and evolutionary mechanisms forming these distribution patterns. However, these inquiries will depend on a sound taxonomic framework followed by detailed phylogenetic analyses within the genus and within Staphylinini, the higher taxon where it belongs.

**Figure 1.**
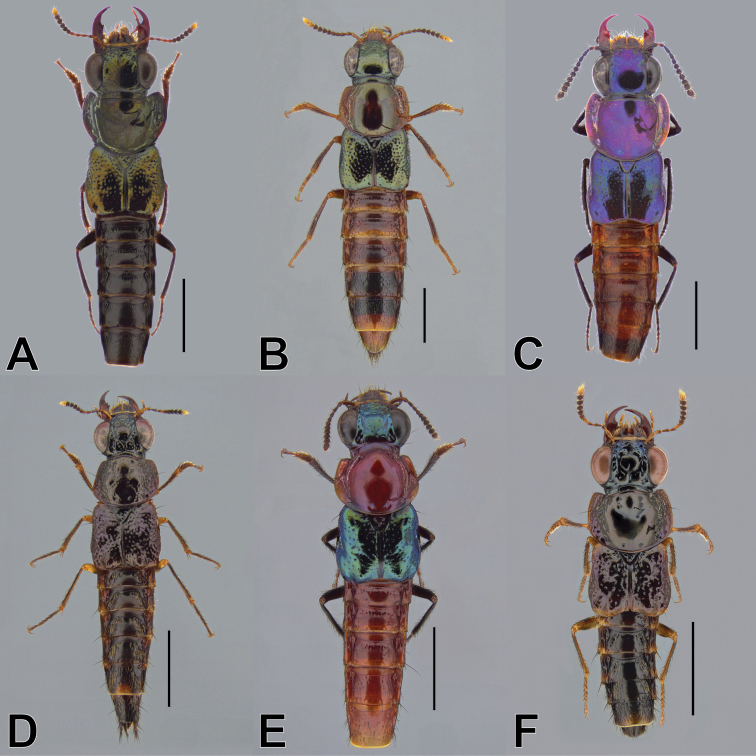
Dorsal habitus of *Bolitogyrus buphthalmus* (Erichson) (**A**), *Bolitogyrus fulgidus* (Sharp) (**B**), *Bolitogyrus pulchrus* Brunke (**C**), *Bolitogyrus bullatus* (Sharp) (**D**), *Bolitogyrus ashei* Brunke (**E**) and *Bolitogyrus tortifolius* Brunke (**F**). Scale bars = 2 mm.

**Figure 2.**
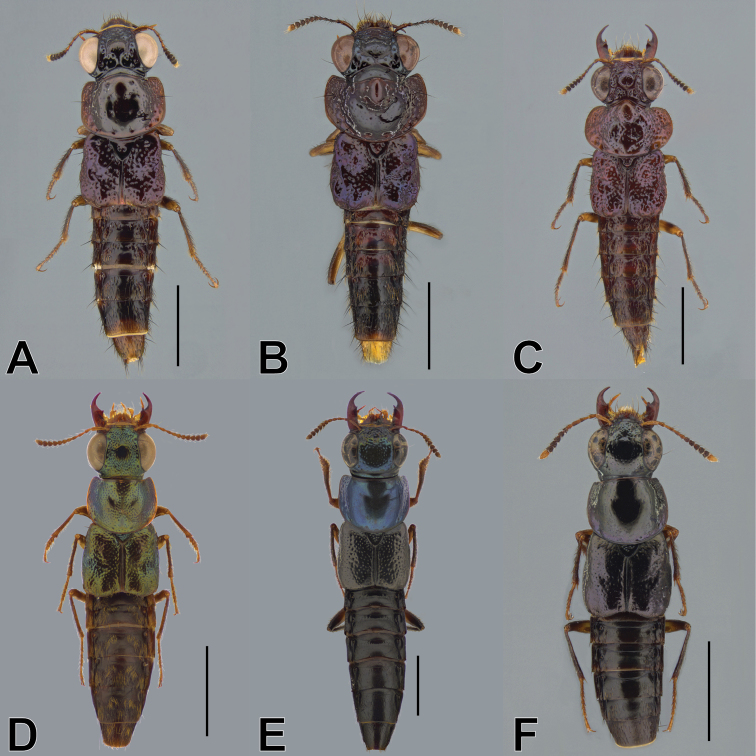
Dorsal habitus of *Bolitogyrus divisus* Brunke (**A**), *Bolitogyrus cornutus* Brunke (**B**), *Bolitogyrus bufo* Brunke (**C**), *Bolitogyrus viridescens* Brunke (**D**), *Bolitogyrus bechyneorum* (Scheerpeltz) (**E**) and *Bolitogyrus marquezi* Brunke (**F**). Scale bars = 2 mm.

Despite the vivid coloration and relatively large size (> 1 cm) of some species (Figs 1–2), *Bolitogyrus* is poorly known taxonomically and rare in collections. It is a member of the diverse (> 5700 species (A. Newton, unpublished database)) and globally distributed rove beetle tribe Staphylinini (Staphylininae) but its sister and genus group relationships remain unclear. Recent molecular and morphological phylogenetic analyses of Staphylinini (Chatzimanolis et al. 2010, Brunke and Solodovnikov 2013) have recovered several well-defined groups including a hyper-diverse ‘Staphylinini propria’, the Amblyopinina and the relictual ‘Quedionuchus lineage’ The latter two groups were formerly classified within the classical but artificial subtribe Quediina (*sensu* Herman 2001b) based on plesiomorphic morphology of the pronotum (Brunke and Solodovnikov 2013). However, the majority of the taxa previously or presently in Quediina, including *Bolitogyrus*, are still of unknown position (table 1 in Chatzimanolis et al. 2010) and represent the most challenging problem in the systematics of Staphylinini, termed the ‘*Quedius*-complex’ by Solodovnikov (2009) and discussed in Brunke and Solodovnikov (2013, figure 1). Thus far, *Bolitogyrus* has been recovered as the sister group to the Oriental genus *Indoquedius* Blackwelder (morphology, Brunke and Solodovnikov 2013) or as an isolated taxon, outside of any known lineages included in an analysis of molecular data (Chatzimanolis et al. 2010). Additional molecular analyses specifically targeting the resolution of the *Quedius*-complex are in progress (Brunke et al., *in prep.*) and will include a greater sample of taxa and genes.

The monophyly of *Bolitogyrus* has never been rigorously tested but Smetana (1995) included two Taiwanese species in a morphological phylogenetic analysis, which placed them as sister taxa based on five unique, derived characters. The majority of, especially recent, taxonomic work on *Bolitogyrus* has focused on the Oriental species and the genus lacks a modern redescription based on the world fauna. The primary sexual characters of the Neotropical species have never been studied and the only key to species (Scheerpeltz 1974) relies on coloration. In recent years, a relatively large number of specimens have accumulated in a few collections due to targeted fieldwork in the Neotropics and indicate a much greater biodiversity than previously known. Therefore, the present work aims to redescribe *Bolitogyrus* based on the world fauna, revise the Neotropical species of the genus, describe previously unrecognized Neotropical diversity, and summarize all available data on these taxa. A similar treatment of the Oriental *Bolitogyrus* is in preparation by the first author.

## Material and methods

### Specimens

This study is based on 715 specimens that are deposited in the following collections:

BMNH Natural History Museum, London, U.K. (R. Booth)

CMNH Carnegie Museum of Natural History, Pennsylvania, U.S.A. (R. Davidson)

CNC Canadian National Collection of Insects, Ontario, Canada (A. Davies, A. Smetana)

CZUG Centro de Estudios en Zoología, Universidad de Guadalajara, Jalisco, Mexico (J. Navarette-Heredia)

DEBU University of Guelph Insect Collection, Ontario, Canada (S. Marshall)

SDEI Senckenberg Deutsches Entomologisches Institut, Müncheberg, Germany (S. Blank)

FMNH Field Museum of Natural History, Illinois, U.S.A. (J. Boone, M. Thayer, A. Newton)

INBIO Instituto Nacional de Biodiversidad, Heredia, Costa Rica (A. Solís)

MZFC Museo de Zoología, Mexico City, Mexico (J. Márquez)

NHRS Naturhistoriska Riksmuseet, Stockholm, Sweden (J. Bergsten)

NMW Naturhistorisches Museum Wien, Vienna, Austria (H. Schillhammer)

PTC Personal collection of Paul Thomas, Illinois, U.S.A. (P. N. Thomas)

SEMC Snow Entomological Collection, Biodiversity Institute, Kansas, U.S.A. (Z. Falin)

UAEH Universidad Autónoma de Estado Hidalgo, Hidalgo, Mexico (J. Márquez)

USNM National Museum of Natural History, Washington D.C., U.S.A. (F. Shockley, D. Furth)

UTCI University of Tennessee at Chattanooga, Tennessee (S. Chatzimanolis)

ZMHB Museum für Naturkunde der Humboldt-Universität, Berlin, Germany (M. Uhlig)

ZMUC Zoological Museum, Natural History Museum of Denmark, University of Copenhagen, Denmark.

### Specimen dataset

Specimens without GIS coordinates on their labels were georeferenced using either Google Earth or Fuzzy Gazetteer (http://isodp.hof-university.de/fuzzyg/query/). Localities published in the Biologia Centrali-Americana (Sharp 1888) were georeferenced using Selander and Vaurie (1962). Specimens with only country or ‘country, state’ data were not georeferenced. Specimen data were exported to Darwin Core Archive (DwCA) format, are available under a Creative Commons CCZero 1.0 License (http://ipt.pensoft.net/ipt/resource.do?r=neobolito1) and were registered with GBIF.

### Microscopy, illustration and photography

All specimens were examined using a Leica MZ APO stereomicroscope. Specimens to be dissected were relaxed in distilled water and then the genital segment (and sometimes abdominal segment VIII) was removed. Genitalia were cleared in a 10% potassium hydroxide solution and then washed with distilled water, then with 70% alcohol and finally placed in glycerin for observation. Genitalia were placed in glycerin-filled vials for long-term storage, which were pinned with their respective specimen.

Line illustrations were digitally drawn from reference photographs in Adobe Illustrator CS v5.1. All photographs were taken using a Leica DFC 420 camera attached to a Leica MZ16A microscope with the help of Leica Application Suite (Leica Microsystems). Photomontage was accomplished using Zerene Stacker (Zerene Systems LLC) and photos were edited in Adobe Photoshop CS v5.1.

Specimens for examination using scanning electron microscopy were first cleaned of extraneous tissue, dehydrated in 96% ethanol, then acetone and air dried before mounting. Specimens were secondarily mounted on an SEM stub using aluminum tape and sputter coated with platinum/palladium. rSEMs of genitalia were created using the methodology outlined in Cheung et al. (2013) using a JEOL JSM-6335F scanning electron microscope. Images were processed in Adobe Lightroom, combined into an rSEM using Adobe Flash CS v5.1 and exported as SWF files.

### Distribution maps

Distribution maps were created using QGIS (2013), with Natural Earth (naturalearthdata.com) vector layers for countries and states/provinces, and a raster background layer (cross blended hypsometric tints with shaded relief and water) to highlight topographical patterns. Specimens with only country or state-level locality data or considered mislabeled were not included in distribution maps. Cases of suspected mislabeling or incorrect locality names are discussed individually under their respective species.

### Measurements

All measurements were made using an eyepiece ocular micrometer placed in the abovementioned microscope. Measurements were taken as listed below, but only proportional (HW/HL, PW/PL, EW/EL, ESut/PL, PW/HW) and forebody measurements were stated directly in descriptions due to a wide variability in body size. Total body length is generally not diagnostic of *Bolitogyrus* species and was not measured due to the contractile nature of the abdomen. Measurements of the eyes were taken but as very few consistent interspecific or sexual differences were found, these values are not given. In most species, eyes generally occupied a larger proportion of the head width in females but much overlap in variation was observed.

HL: Head Length, at middle, from the anterior margin of frons to the nuchal ridge.

HW: Head Width, the greatest width, including the eyes.

PL: Pronotum Length, at middle.

PW: Pronotum Width, greatest width.

EL: Elytral Length, greatest length taken from level of the anterior most large, lateral macroseta to apex of elytra (this seta can be seen in Fig. 9B, C and F). This length approximates the length of the elytra not covered by the pronotum and therefore contributing to the forebody length.

ESut: Sutural Length, length of elytral suture.

Forebody: HL + PL + EL.

## Data resources

A specimen level dataset was made available as a Darwin Core Archive (http://ipt.pensoft.net/ipt/resource.do?r=neobolito1) and was deposited in GBIF.

## Results

### Species

A revision of the Neotropical *Bolitogyrus* species resulted in the discovery of 19 new species, 2 new synonyms and the revalidation of *Bolitogyrus sallei* (Kraatz), resulting in a total of 28 valid species. The species were organized into five putative species groups to facilitate comparisons between species. Four of these five species groups were included inside a larger lineage concept, the ‘Bullatus Lineage’ (see checklist below). Three species were not placed to any of the species groups and monotypic groups were avoided. Currently, the country with the greatest number of species is Costa Rica with 13 species, followed by Mexico and Panama with 9 species each. However, the available material from each of the latter countries is a third or less of that available from Costa Rica. As far as known, the number of putatively endemic species is greatest in Costa Rica with 8 species known only from that country.

### Distribution

*Bolitogyrus* is distributed in two widely disjunct areas. In the Oriental region, we have seen specimens from southern India, Meghalaya, Nepal and West Bengal, Myanmar south to Java and Borneo, north to Taiwan and Mainland China. The genus was not previously known from Myanmar but several widespread southeast Oriental species occur there (Brunke, *unpublished data*). The northernmost Oriental specimen known is from northeastern Sichuan province, China (Hu et al. 2011). As far as known, *Bolitogyrus* does not occur east of Wallace’s line and does not occur in the Philippines. A more complete Oriental distribution will be available after the species are revised (Brunke, *in prep*). In the Neotropical region, *Bolitogyrus* occurs from Jalisco and Hidalgo in southern Mexico, north to Belize and south to Darién province in Panama, with one species occurring in South America on the eastern slopes of the Andes (*Bolitogyrus cornutus* Brunke, sp. n.) (Fig. 2B). As elaborated on below, *Bolitogyrus* is rarely collected and most countries have been inadequately sampled for members of this genus. Additional collecting in Mexico (especially near the northern distributional limit and in Chiapas), Panama (especially Darién) and in the Andes of South America is expected to yield the greatest number of new species.

### Natural history and specimen collection

Very early on in the taxonomic history of the genus, it was known that species of *Bolitogyrus* were found in or on fungus or fungus covered, rotten logs (e.g., Fauvel (1878), in communication with Sallé). Despite this early knowledge, specimens are typically rare in collections, of which most were found to have only one to three individuals, if any. Based on fieldwork by the author in Costa Rica and from label data from the Neotropical material, it appears that *Bolitogyrus* typically occur only in a small percentage of the logs or standing dead trees in a given area and may favor those near a constant source of moisture (e.g. those in ravines or overhanging streams). In Costa Rica, the majority of fungusy logs were inhabited by species of Xanthopygina (e.g. *Plociopterus* Kraatz) or Philonthina (e.g., *Belonuchus* Nordmann) rather than *Bolitogyrus*, with very little overlap between the former two taxa and the latter. *Bolitogyrus* species are restricted to moist, mostly evergreen forests at varied elevations but most Neotropical species occur at medium elevations (1000-1800 m) and thus far, specimens have not been collected above 2460 m. Only 8 specimens were collected below 500 m; most of these belonged to species represented by few specimens. Virtually nothing is known in detail about the ecology of the genus, though its species are assumed to be predaceous on smaller invertebrates. One unidentifiable female specimen (USNM) of the Cornutus Group from Panama (probably *Bolitogyrus gracilis* Brunke, sp. n.) bore label data recording it from the stomach of a cane toad (*Bufo marinus*).

Although specimens are occasionally collected passively by Malaise or flight intercept traps, far greater success has been achieved by low scale insecticide ‘fogging’. The method involves placing a white cloth sheet underneath or beside a rotten log and spraying the log with a pyrethrum or pyrethroid-based insecticide (those advertising residual activity on the label were not used). Almost immediately afterward, arthropods can be found on the sheet and placed into alcohol or another killing agent. Just over half of all specimens included in the present revision were collected by this method (50.8%, 363/715) compared to flight-intercept (11.2%, 80/715) or Malaise traps (5.5%, 39/715). The remaining specimens were mostly collected by hand from fungusy wood. The Neotropical *Bolitogyrus* species apparently do not normally inhabit the leaf litter layer as only 1.5% (11/715) of specimens were collected by sifting leaf litter or processing it in a Berlese funnel. The use of fogging in the Neotropics is limited to relatively few localities and has primarily been utilized by staff at SEMC (Kansas, U.S.A) (e.g., J. S. Ashe, Z. Falin) and by P.N. Thomas (Illinois, U.S.A), with most collecting events in Costa Rica. It is therefore unsurprising that the majority of material included herein (81.9%) is derived from these two sources alone.

### Morphology

Morphological terminology generally follows that of Brunke and Solodovnikov (2013), with chaetotaxy of the head and pronotum following that of Smetana (1971) except ‘additional punctures’ along the inner margin of the eye between the anterior and posterior marginal punctures are given the name *oculomarginal punctures* (Fig. 6B). Following Smetana (1988), these punctures are always situated close to the inner eye margin but they are here considered homologous even at a distance from the margin as wide as the puncture depression. In general, the density and distribution of asetose punctures on the head, pronotum and elytra are variable within species such that quantitative descriptions of them were often not possible. However, consistent, mostly qualitative character states of punctation were observed and are described under the respective species and in the identification key. The side of the aedeagus with the paramere attached is considered ventral and is referred to herein as ‘parameral’. Many morphological features observed in *Bolitogyrus* are atypical or unique within Staphylininae and are outlined below.

**Sculpture of the forebody**

Unlike the Oriental species, the Neotropical *Bolitogyrus* possess several raised areas of the cuticle that are diagnostic of species, species groups or large lineages within the genus. On the dorsal side of the head there may be a *central protuberance* (Fig. 6A) and a pair of *posterior head protuberances* (Fig. 6A) that are delimited by impressed areas of the cuticle. In most species of *Bolitogyrus*, the frons has a Y or X-shaped *frontal impression* though it is weakly impressed in the Neotropical species (Fig. 6A) compared to the Oriental species. Anterior to this impression are minor protuberances that are generally not diagnostic, as they may exhibit wide intraspecific variation. In all Neotropical species, there is a pair of medial depressions on the pronotum (Fig. 7B) and in most species, a *pronotal protuberance* between them that is best observed in lateral view (Fig. 7E–H, 8A–E). The elytra may possess *elytral protuberances* (e.g., Fig. 8E) but the size and shape of these are rather variable within species and were generally not found to be of diagnostic value.

**Sexual dimorphism of the pronotum**

The pronotal protuberance can be developed in the following states: absent in both sexes (e.g., *Bolitogyrus bechyneorum* Scheerpeltz); distinct in males but barely visible in females (e.g., *Bolitogyrus costaricensis* Wendeler) (Fig. 7E–F); more or less equally well developed in both sexes (e.g. *Bolitogyrus divisus* Brunke, sp. n.) (Fig. 7G–H); or produced into a horn in males but not in females (e.g. *Bolitogyrus bufo* Brunke, sp. n.) (Fig. 8A–B). In *Bolitogyrus cornutus*, a male was observed with a ‘female-like’ pronotum (Fig. 8D), demonstrating polymorphism for these traits (Fig. 8C–E). An analogous pattern of male polymorphism is known in dung beetles (Scarabaeidae: Scarabaeinae) where female-like, smaller males gain access to copulations with females by avoiding male-male competition (Emlen 1997). Although male-male conflict has been observed in the subfamily Staphylininae (Alcock 1991), the use of horns or similar structures to gain access to females has not been reported, though these structures exist in several rove beetle genera belonging to other rove beetle subfamilies. Measurements of additional ‘pseudofemale’ males in *Bolitogyrus* and behavioral observations in this genus are necessary to advance this beyond speculation.

**Margin of the pronotum**

The pronotal margin of all *Bolitogyrus* species is more or less expanded and explanate, at least narrowly (Fig. 7A–D); only in some Oriental species does it become strongly expanded basally and basolaterally (Fig. 7A). In many Neotropical species, the lateral portions of the pronotum have become strongly explanate rather than the margin itself (Fig. 7B–D).

**Abdomen**

In all Neotropical species of *Bolitogyrus*, the anterior transverse basal carina of each abdominal tergite is extended posteriad and reaches or exceeds the level of the spiracles (Fig. 12B, E; 13E–F). In the Oriental species, this line is transverse or, rarely, sinuate (Fig. 12A). If demonstrated to be synapomorphic, this character would indicate a single origin of the Neotropical species.

**Division of the median lobe of the aedeagus**

In some Neotropical species, the median lobe has become divided into two *lateral lobes* (Fig. 19K; 20A, H) or bears a *median suture* in its apical third (e.g., Fig. 21L) (Divisus and Cornutus Groups), which may indicate a re-fusion of the lateral lobes. In *Bolitogyrus cornutus*, the median lobe bears a medioapical notch and more strongly sclerotized areas that trace an outline similar in shape to the lateral lobes (Fig. 21J).

**Female genital segment**

In many *Bolitogyrus* species, female tergite X is typical of Staphylinini and entirely separate from laterotergal sclerites IX (Fig. 25A–L; 26A–F). In these species the disc of tergite X may be raised above the plane of the lateral portions to create a shape that is often diagnostic (e.g., Fig. 26A). The basal margin of female tergite X in other species (e.g., Bullatus Lineage) may be thickened and fused with the basal margin of laterotergal sclerites IX (Fig. 26H). This fused structure may be expanded posteriad to form an *accessory sclerite* (Fig. 26G), which can become independent of laterotergal sclerites IX (e.g., the Divisus and Cornutus Groups) (Fig. 27C). Some species with an accessory sclerite may have the basal half of tergite X strongly reduced (Fig. 27C). The medial margins of laterotergal sclerites IX in the Bullatus Lineage have become variably expanded toward the center, covering portions of tergite X (Fig. 27C). In *Bolitogyrus cheungi* Brunke, the laterotergal sclerites have expanded and fused overtop tergite X, which is reduced to an anchor-shaped sclerite (Fig. 27F). Species with complex female genitalia tend to have males with complex aedeagi and the functional morphology of these characters warrants further research.

## Taxonomy

### Bolitogyrus

Taxon classificationAnimaliaColeopteraStaphylinidae

Chevrolat, 1842

Bolitogyrus Chevrolat, 1842: 641. Type species: *Quedius buphthalmus* Erichson, 1840: 534, fixed by monotypy (see Herman 2001a).
Cyrtothorax Kraatz, 1858: 366. Type species: *Cyrtothorax sallei* Kraatz, 1858: 367, fixed by subsequent designation by Scheerpeltz 1974.
Cyrtothorax ; Kraatz 1858 (description of genus, species included: *sallei*, *erythrurus*), Fauvel 1878 (key to species), Sharp 1884 (notes), Cameron 1932 (key to species of ‘British India’), Blackwelder 1952 (type species designation, invalid, see Herman 2001b), Scheerpeltz 1962 (key to genera of Quediini), Scheerpeltz 1974 (type species designation, key to world species), Shibata 1986 (species list, Taiwan), Smetana 1988 (synonym of *Bolitogyrus*)
Bolitogyrus ; Blackwelder 1952 (type species designation, invalid, *cribripennis* not available), Hammond 1984 (checklist of Borneo species), Smetana 1988 (characters, discussion of availability of *Bolitogyrus* and its type species), Smetana 1995 (characters, key to species of Taiwan), Smetana 2000 (key to species of mainland China), Herman 2001a (discussion of availability of *Bolitogyrus* and its type species), Rougemont 2001 (microhabitat notes), Navarette-Heredia et al. 2002 (key to genera of Staphylininae, distribution in Mexico and Neotropics), Yuan et al. 2007 (characters, incomplete key to mainland Chinese species), Hu et al. 2011 (characters, key to mainland Chinese species).

#### Taxonomic history.

The first instance of the genus group name ‘*Bolitogyrus*’ was in Dejean (1836) as ‘*Bolitogyrus cribripennis*’, however both the genus and species names were *nomina nuda* and unavailable as no description was published. Chevrolat (1842) mentioned that he had sent ‘*Bolitogyrus cribripennis*’ to Erichson, who considered it synonymous with *Quedius buphthalmus* Erichson, 1840 from ‘Mexico’. No subsequent authors, or Chevrolat (1842) provided characters for ‘*Bolitogyrus cribripennis*’ and thus, the name remains unavailable. However, as argued by Herman (2001a), Chevrolat’s acceptance of Erichson’s identification made *Bolitogyrus* available by indication and by monotypy, *Quedius buphthalmus* Erichson became the type species of the former genus group name. This concept is accepted herein.

Kraatz (1858) described the genus *Cyrtothorax* for two new species: *Cyrtothorax sallei* from ‘Mexico’ and *Cyrtothorax erythrurus* from ‘Nova Grenada’ (=Panama + Colombia). No mention was made to either *Bolitogyrus* or *Quedius buphthalmus*, and it can be assumed that Kraatz was unaware of Erichson’s species and the obscure literature records of the name *Bolitogyrus*. Fauvel (1878) was the first to associate *Bolitogyrus*, *Cyrtothorax* and *Quedius buphthalmus* together. However, he attributed *Bolitogyrus* to Dejean, listed it as a junior synonym of *Cyrtothorax* and listed *Cyrtothorax sallei* as a junior synonym of *Quedius buphthalmus*, all without explanation. In an influential catalog by Blackwelder (1952), *Bolitogyrus* was listed as a senior synonym of *Cyrtothorax* for reasons not considered under the Code as valid (summarized by Herman 2001a). Nevertheless, other, later authors (Hammond 1984, Smetana 1988) accepted Blackwelder’s opinion and helped to establish *Bolitogyrus* as the correct name for this genus. Until 1988, both names appeared in the literature.

Fauvel (1858) was the first to recognize that the genus also occurred in the Oriental region and described *Cyrtothorax vulneratus* from ‘Cochinchine’ (= southern Vietnam) and *Cyrtothorax carnifex* from Cambodia. In the Biologia Centrali Americana, Sharp (1884) described *Cyrtothorax cyanescens* and *Cyrtothorax salvini* from Guatemala, *Cyrtothorax fulgidus* from Nicaragua and *Cyrtothorax bullatus* from Panama. Bernhauer (1915) extended the range of *Bolitogyrus* to the island of Borneo with his description of *Cyrtothorax caesareus*. Wendeler described *Cyrtothorax costaricensis* from Costa Rica in 1927 and a year later described *Cyrtothorax strigifrons* from Mexico. Cameron described *Cyrtothorax signatus* from Ceylon (=Sri Lanka) in 1932, *Cyrtothorax elegans*, *Cyrtothorax octomaculatus*, and *Cyrtothorax rufipennis* from Java in 1937, and *Cyrtothorax borneensis* and *Cyrtothorax proximus* from Borneo in 1942. Scheerpeltz (1974) provided a review of the entire genus (as *Cyrtothorax*), an identification key and descriptions of five new species: *Cyrtothorax bechyneorum* from El Salvador, *Cyrtothorax doesbergi* from Java, *Cyrtothorax fukiensis* from southeast China, *Cyrtothorax nevermanni* from Costa Rica and *Cyrtothorax vietnamensis* from Vietnam. Unfortunately, the key is difficult to use as it relies almost entirely on coloration and cannot accommodate the considerable color variation often encountered within species of this genus.

Shibata (1979) was the first to study and illustrate the aedeagus of *Bolitogyrus* and described *Cyrtothorax rufomaculatus* from Taiwan. Following Shibata (1979), all further descriptions of *Bolitogyrus* included the aedeagus if the male was known. Zheng (1988) described *Cyrtothorax cyanipennis* from China and Hayashi (1991) described *Cyrtothorax taiwanensis* from Taiwan. Smetana and Zheng (2000) described a further three species from China: *Bolitogyrus electus*, *Bolitogyrus kitawakii* and *Bolitogyrus pictus*. Smetana (2000) described *Bolitogyrus nigropolitus* from Sichuan, China and provided a key to the species known from Mainland China. He also introduced a lineage concept for the Chinese species based on the punctation of the anterior angles of the pronotum. Rougemont (2001) extended the range of *Bolitogyrus* to southern India with his description of *Bolitogyrus lasti* and Yuan et al. (2007) described three more species from China: *Bolitogyrus elegantulus*, *Bolitogyrus flavus* and *Bolitogyrus nigerrimus*. The latter authors also developed upon the lineage concept of Smetana (2000) by adding characters of the prosternum and female tergite VIII. Finally, Hu et al. (2011) described *Bolitogyrus huanghoi* from China and updated the key to the Mainland Chinese species.

Unfortunately, no Neotropical species of *Bolitogyrus* have been described or otherwise treated since Scheerpeltz (1974) and the male sexual characters of these species remain unknown.

#### Diagnosis.

*Bolitogyrus* can be easily recognized among other Staphylinini by its characteristic habitus (Figs 1–2) and the following five characters: antennomeres I–V without dense, tomentose pubescence (Fig. 5A–E); lateral face of hind tibia without spines, only setae (Fig. 11F); eyes strongly convex and occupying nearly entire lateral head length (Fig. 6A–G); disc of head and pronotum without microsculpture (sometimes fragments of microsculpture present around roughly sculptured areas); elytral punctures not limited to discrete rows and without appressed setae typical of Staphylinini, punctures asetose except those bearing erect (usually long and coarse) macrosetae in a sutural, discal and lateral row (best visible in Fig. 9B and 10D). Among the taxa not belonging to Staphylinini Propria (*sensu* Brunke and Solodovnikov 2013), the antennae pubescence, spineless lateral face of the hind tibia and the elytral punctation are potential, unique synapomorphies of *Bolitogyrus*.

Diagnoses given previously for *Bolitogyrus* (e.g., Smetana 1988, Hu et al. 2011) have included a V-shaped impression on the frons, markedly explanate basal and posterolateral pronotal margins and a mesotibia without spines on its lateral face. These character states were found to apply only to a limited portion of the genus, especially concerning the Neotropical species. The frontal impression is weakly formed in the Neotropical species and a few species (e.g., *Bolitogyrus newtoni* Brunke) either lack the impression on the frons or have it barely discernable. The markedly explanate margins of the pronotum are most strongly developed in the Oriental species, especially in the non-ridged prosternum group *sensu* Yuan et al. (2007) (e.g., *Bolitogyrus pictus* Smetana & Zheng (Fig. 7A)). The margins of those Oriental species with a ridged prosternum (e.g., *Bolitogyrus electus* Smetana & Zheng) are much less expanded, and those of the Neotropical region are barely expanded at all (Fig. 7B–D). In many species of *Bolitogyrus*, the lateral face of the mesotibia does bear spines, though they are thinner than in most genera of Staphylinini and often hidden amongst setae of the same length (Fig. 11D). In *Bolitogyrus marquezi* Brunke, these spines are distinct (Fig. 7E) and as well-developed as in some species of *Indoquedius* Blackwelder. However, the lateral face of the metatibia in all examined *Bolitogyrus* lacks spines (Fig. 7F) and this character state may be a synapomorphy of the genus.

Due to the glossy body surface and large eyes, *Bolitogyrus* is most similar in habitus to *Anaquedius* Casey, *Astrapaeus* Gravenhorst, *Hemiquedius* Casey, *Indoquedius*, *Parisanopus* Brèthes, *Quedius (Cyrtoquedius)* Bernhauer and *Quwatanabius* Smetana but can be distinguished from these genera solely by the irregular, mostly asetose elytral punctation or glabrous antennomeres I–V.

#### Redescription.

Large to medium sized, glossy Staphylinini, often with bright metallic reflections or contrasting patterns of red or yellow and black, legs often yellow with dark bands.

*Head* moderately to strongly transverse; dorsally without microsculpture except as fragments near coarse sculptured areas; eyes large and strongly convex, occupying nearly entire lateral head length, temple almost non-existent; neck constriction well-developed; medial frontoclypeal puncture present (*e.g*., ‘f’ in Fig. 6C; with two to four, rarely five or more, oculomarginal punctures along inner margin of eye (‘d’ in Fig. 6B); without additional punctures between anterior frontal punctures; with only one vertical puncture at base of head; antenna subclavate, non-geniculate, antennomeres VI–XI wider and often more darkly colored than preceding, I–V without tomentose pubescence; labrum broad, with lateral areas poorly sclerotized, with medial incision; mandible large, slender, apical half distinctly more slender and curved mediad, with dorsolateral groove, with two teeth, basal tooth with blunt apex, apical tooth much smaller and acute, left mandible with teeth on same plane, right mandible with apical tooth below dorsal plane of mandible, some species with an additional, minute tooth just basad of the basal tooth; ventral surface of head with fine microsculpture of transverse waves; maxillary palpi with apical palpomere fusiform, glabrous to sparsely covered with minute, pale setae in some species, and longer and narrower than previous segment; labial palpi with apical palpomere elongate to broad fusiform, as wide to narrower than previous segment, glabrous to distinctly setose; mentum transverse, alpha seta present, beta seta present (fig 7B in Brunke and Solodovnikov 2013); ligula with only minute notch apically; gular sutures strongly convergent posteriad, nearly touching and running parallel from midlength to near base; postgenal and ventral basal ridges present; nuchal ridge present dorsally and laterally; in most species, infraorbital ridge appearing as absent, some species with infraorbital ridge present as short, fine fragment posteriad of ventral extension of nuchal ridge; ridge of unknown homology extending from base of mandible and fusing with nuchal ridge/infraorbital ridge, forming a sharp angle at the point of fusion; dorsal basal ridge absent.

*Pronotum* slightly to strongly transverse, widest anteriorly, convex, surface without microsculpture except as fragments near areas of coarse sculpture, basal and posterolateral margins at least slightly expanded and explanate (Fig. 7B), markedly sharply explanate and expanded in some Oriental species (Fig. 7A); with pair of impressions medially (Fig. 7B), developing medially into an, often sexually dimorphic, pronotal protuberance in some species (Neotropical species) (Fig. 8A–B) or without impressions or a protuberance (nearly all Oriental species) (Fig. 7A); dorsal rows of pronotum with one to three punctures; hypomeron partially visible in lateral view; inferior line of hypomeron present, ending near anterior coxa, not connected to superior line; postcoxal process well-developed but membranous; pronotum and prosternum not fused inside procoxal cavity; prosternum acute apically and produced ventrad (Fig. 11C), with only scattered, short setae medially, pair of medial macrosetae absent, sharp median carina not present (Fig. 11C), basisternum without (most species) or with rounded longitudinal ridge (several Oriental species).

*Elytra* with dorsal surface varying from slightly but evenly convex to highly uneven with protuberances, entire surface irregularly, sparsely punctate, most punctures asetose but those in sutural, discal and lateral rows with coarse, erect macrosetae (Fig. 9B–F); scutellum coarsely punctate (punctures transversely rugose in Strigifrons Group), posterior scutellar ridge present (Fig. 10E–F); subbasal ridge present, sinuate and directed anteriad (Fig. 10E) (horizontal and connected to humerus in Strigifrons Group and *Bolitogyrus marquezi* (Fig. 10E)); hind wings fully developed, MP4 and CuA veins separate, vein MP3 present (Fig. 9A); row of humeral spines present (*e.g.*, Fig. 10B); intercoxal area of mesocoxal acetabulum distinctly recessed compared to metaventrite and intercoxal process, mesocoxae therefore contiguous.

*Procoxa* with anterior portion of external ridge fading out and not extending beyond half the length of coxa in most species (fig. 9E in Brunke and Solodovnikov 2013) (external ridge extended beyond half the length of coxa in some Neotropical species); procoxa with internal ridge present and extended dorsad nearly the entire length of coxa (fig. 9F in Brunke and Solodovnikov 2013); metacoxa without transverse carina; mesotibia not heavily spinose, spines thin and usually inconspicuous among setae of similar length, in some species, mesotibia entirely without spines; metatibia without spines on lateral face, medial face sometimes with one or two small, thin spines; protarsomeres with dorsal surface setose, setae not restricted to margins; metatarsomeres II–V setose on dorsal surface; metatarsomeres IV with ventral spine-like setae distinctly interrupted medially and removed from apical margin (fig. 9A in Brunke and Solodovnikov 2013); one pair of empodial setae present on all tarsi, pairs of length subequal to each other, shorter than tarsal claws; both sexes without brush of adhesive setae on basal mesotarsomere; both sexes with tenant setae on ventral face of protarsus, in many species protarsus slightly more expanded in males than in females.

*Abdomen* with prototergal glands present and with well-developed acetabulum; abdominal tergites III–V deeply impressed at base, impression with coarse punctures; disc of abdominal tergites III–V or III–VI impunctate at middle (*e.g.*, Fig. 12A, B, E); abdominal tergite II with basal longitudinal carina entire, connecting anterior margin of tergite with transverse basal carina; abdominal tergites with only anterior transverse basal line, without pair of accessory ridges or curved ridge (*e.g.*, Fig. 12A); abdominal sternite III with basal transverse carina sharply produced at middle and forming an acute angle (Fig. 14E); sternite VII without porose structure; male sternite VIII with vaguely emarginate to deeply incised apical margin, triangular area near emargination more sparsely punctate than surround area to entirely glabrous, varying in size, male sternite VI and VII sometimes with similar modifications; in some species, female tergite VIII with small, semicircular to elongate notch; aedeagus with parameres fused into one solid sclerite (‘the paramere’), paramere often divided into two lobes in apical half or with median seam; paramere with peg setae (except *Bolitogyrus cornutus*); median lobe of aedeagus variable, apical half divided into two lobes in several Neotropical species; internal sac of aedeagus with only small sclerites, in some species (*Bolitogyrus marquezi* and the Strigifrons Group) with one or a pair of more strongly sclerotized sclerites; female gonocoxite with apical stylus; female tergite X in many species with raised disc (*e.g.*, Fig. 25A–F), in some species (Bullatus Lineage) basal portion sometimes strongly reduced and covered at middle by expanded medial portions of the laterotergal sclerites and basal by an accessory sclerite (*e.g.*, Fig. 27E). Females with spermatheca unsclerotized.

#### Checklist of Neotropical *Bolitogyrus*

Buphthalmus Group

*Bolitogyrus buphthalmus* (Erichson, 1840)

*Bolitogyrus costaricensis* (Wendeler, 1927)

*Bolitogyrus erythrurus* (Kraatz, 1858)

*Bolitogyrus fulgidus* (Sharp, 1884)

*Bolitogyrus pulchrus* Brunke, sp. n.

*Bolitogyrus sallei* (Kraatz, 1858) stat. r.

*Bolitogyrus salvini* (Sharp, 1884)

Bullatus Lineage

Unplaced

*Bolitogyrus bullatus* (Sharp, 1884)

*Bolitogyrus apicofasciatus* Brunke, sp. n.

Ashei Group

*Bolitogyrus ashei* Brunke, sp. n.

*Bolitogyrus tortifolius* Brunke, sp. n.

*Bolitogyrus pseudotortifolius* Brunke, sp. n.

Cornutus Group

*Bolitogyrus cornutus* Brunke, sp. n.

*Bolitogyrus brevistellus* Brunke, sp. n.

*Bolitogyrus bufo* Brunke, sp. n.

*Bolitogyrus cheungi* Brunke, sp. n.

*Bolitogyrus gracilis* Brunke, sp. n.

*Bolitogyrus longistellus* Brunke, sp. n.

*Bolitogyrus thomasi* Brunke, sp. n.

Divisus Group

*Bolitogyrus divisus* Brunke, sp. n.

*Bolitogyrus falini* Brunke, sp. n.

*Bolitogyrus inexspectatus* Brunke, sp. n.

Strigifrons Group

*Bolitogyrus strigifrons* (Wendeler, 1928)

*Bolitogyrus silex* Brunke, sp. n.

*Bolitogyrus viridescens* Brunke, sp. n.

Unplaced *Bolitogyrus*

*Bolitogyrus bechyneorum* (Scheerpeltz, 1974)

*Bolitogyrus marquezi* Brunke, sp. n.

*Bolitogyrus newtoni* Brunke, sp. n.

Undescribed species

#### Key to the Neotropical species of *Bolitogyrus*

**Table:** 

1	Head without central protuberance delimited by depressed areas of the cuticle (Fig. 6B)	2
–	Head with central protuberance delimited by depressed areas of the cuticle (Fig. 6A)	Bullatus Lineage, 12
2	Antenna dark brown, antennomere II with dark reddish base (Fig. 5E); known only from the Talamanca mountains in Costa Rica and Panama; aedeagus as in Fig. 16A–B	*Bolitogyrus pulchrus* Brunke, sp. n.
–	Antennomeres I–V at least partially pale, distinctly contrasting with VI–X in most specimens (Fig. 5A)	3
3	Abdominal segment VIII and genital segment distinctly yellow to orange, contrasting with previous segment (Fig. 12D)	4
–	Abdominal segment VIII and genital segment dark: black or brownish, not strongly contrasting with previous segment (Fig. 12C)	6
4	Surface of elytral disc without distinct protuberances, rather evenly convex (Fig. 9B); elytra with epipleuron and extreme base reddish, contrasting with blue to green metallic color on disc (Fig. 10A); female tergite X distinct: sharply produced at apex and with unique beveled apical area (Fig. 26E–F); female secondary gonocoxite swollen at base (Fig. 27H); known from Nicaragua to Costa Rica (not east of the Central Cordillera)	*Bolitogyrus fulgidus* (Sharp) [male unknown]
–	Surface of elytral disc with protuberances, distinctly uneven (Fig. 10B); elytra entirely reddish or entirely dark, with metallic reflections (Fig. 10B); female tergite X less sharply produced at apex, without beveled apical area (Fig. 25I–K; 26A–C); female secondary gonocoxite regularly shaped, not distinctly swollen (Fig. 27G); Mexico to Panama	5
5	Female tergite X with raised area on disc that is slender and subparallel in basal half (Fig. 26A–C); known from Chiapas, Mexico to central Nicaragua; aedeagus as in Fig. 16C–H	*Bolitogyrus salvini* (Sharp) (morphotypes I and II).
–	Female tergite X with raised area on disc that is broader at base and distinctly narrowing to apex in basal half (Fig. 25I–K); distributed in Talamanca mountains (southern Costa Rica and northern Panama)	*Bolitogyrus erythrurus* (Kraatz) [male unknown]
6	Antennomere VI quadrate when viewed in widest profile (Fig. 5C); disc of elytra with small, poorly impressed punctures (Fig. 9D); mesotibia with distinct spines, not obscured by setae of the same length (Fig. 11E); lateral area of abdominal tergites III–VII with characteristic short, appressed setae (Fig. 12F); known only from Mexico; aedeagus as in Fig. 17D–F	*Bolitogyrus marquezi* Brunke, sp. n.
–	Antennomere VI transverse when viewed in widest profile (Fig. 5B); disc of elytra with most punctures more strongly impressed, creating wider depressions (Fig. 9B, C and E); mesotibia with, at most, thin spines obscured by setae of the same length (Fig. 11D); lateral area of abdominal tergites III–VII with longer setae that are not appressed (Fig. 12E)	7
7	Disc of elytra more or less even, punctation dense and more regularly spaced (Fig. 9C); forebody bright metallic blue to blue-green, elytra with dull silver reflection in clean specimens (Fig. 2E), elytra never bright metallic purple or bronze; male with sternites VII and VIII deeply impressed and distinctly emarginate (Fig. 13B); known from Mexico to central Nicaragua; aedeagus as in Fig. 16J–L	*Bolitogyrus bechyneorum* Scheerpeltz
–	Disc of elytra uneven, punctation more irregularly spaced (Fig. 9E; 10B); forebody with metallic reflection of variable color, including blue, elytra bronze, dark green, greenish-bronze, purple or bluish-purple, never with silvery reflection; male with sternites VII and VIII not deeply impressed or distinctly emarginate (Fig. 13A)	8
8	Apical antennomere not paler than previous subapical segments (Fig. 5D); apical half of sternites with sparse microsculpture, interspaces many times wider than lines (Fig. 13C); male with forecoxa entirely dark (Fig. 11C); female with distinctively shaped tergite X (Fig. 25H); forebody 4.0–4.5 mm; known from southern Mexico to Guatemala; aedeagus as in Fig. 17A–C	*Bolitogyrus newtoni* Brunke, sp. n.
–	Apical antennomere distinctly paler than previous subapical segments in nearly all specimens, always at least slightly paler (Fig. 5A, B); apical half of sternites with fine microsculpture, width of interspaces subequal to that of lines (Fig. 13D); male with forecoxa entirely to mostly pale (Fig. 11A, B); female with tergite of a different shape (Fig. 25A–F, L; 26A–C); forebody length variable; Mexico to Costa Rica; dissected males needed to continue	9
9	Parameral arms divergent (Fig. 16C–E, I)	10
–	Parameral arms convergent (Fig. 15A–B, E–F)	11
10	Median lobe in parameral view narrow (Fig. 16I); female forecoxa at least partially pale (Fig. 11B); female tergite X broader (Fig. 25L); known from Veracruz and Hidalgo in Mexico	*Bolitogyrus sallei* (Kraatz)
–	Median lobe in parameral view broader (Fig. 16C); female forecoxa completely dark (c.f. Fig. 11C); female tergite X narrower (Fig. 26A–C); known from Chiapas, Mexico	*Bolitogyrus salvini* (Sharp) (morphotype III)
11	Peg setae fields of parameral arms obtuse at base (Fig. 15G–H); aedeagus recurved in lateral view (Fig. 15F)	*Bolitogyrus costaricensis* (Wendeler)
–	Peg setae fields of parameral arms acute at base (Fig. 15C–D); aedeagus projected ventrad in lateral view (Fig. 15B)	*Bolitogyrus buphthalmus* (Erichson)
12	Elytra with patch of strigulose sculpture, creating metallic reflection different in color from surrounding surface in clean specimens (Fig. 9F); abdominal tergites with dense, yellow setae organized in patches (Fig. 13E–F)	Strigifrons Group, 13
–	Elytra coarsely punctate, without strigulose sculpture; abdominal tergites with only sparse yellow setae, organized in patches or not (Fig. 14A–D)	15
13	Base of head with a pair of large, glossy, protuberances, creating expansive impunctate areas (Fig. 6A); Guatemala; aedeagus as in Fig. 17L–O	*Bolitogyrus silex* Brunke, sp. n.
–	Base of head without large glossy protuberances, a few specimens with a pair of small, well separated protuberances that do not create expansive impunctate areas (Fig. 6G); Mexico	14
14	Base of head with strongly strigulose sculpture (Fig. 6F); tergite VI with impunctate area medially (Fig. 13E); aedeagus as in Fig. 17G–K	*Bolitogyrus strigifrons* (Wendeler)
–	Base of head with strongly impressed, often rugose punctures (Fig. 6G); tergite VI without impunctate area medially (Fig. 13F); aedeagus as in Fig. 18A–E	*Bolitogyrus viridescens* Brunke, sp. n.
15	Pronotum with only one setose puncture in the dorsal row (the anterior marginal puncture) (Fig. 7B, arrow)	16
–	Pronotum with at least two setose punctures in the dorsal row (Fig. 7C–D, arrows)	18
16	Scutellum orange-red, paler than metallic green or purple elytra (Fig. 1E); discoid protuberance of head weakly delimited, bordered only laterally by a pair of linear, sub-parallel furrows (Fig. 6E); aedeagus as in Fig. 19A–C	*Bolitogyrus ashei* Brunke, sp. n.
–	Scutellum dark, not contrasting with elytra (Fig. 1D); elytra not metallic; discoid protuberance of head well delimited by depressed areas around its circumference (Fig. 6C–D)	17
17	Hind femur with dark band relatively close to apex, separated from apex by a distance distinctly less than half its length (Fig. 11H); paired protuberances on head, if present, weakly formed and not separated by a narrow channel (Fig. 6D); tergite VII with apex evenly paler (Fig. 14B); distributed in southern Mexico and Guatemala; aedeagus as in Fig. 18F–H	*Bolitogyrus apicofasciatus* Brunke, sp. n.
–	Hind femur with dark band relatively far from apex, separated from apex by a distance distinctly greater than half its length (Fig. 11G); base of head with a pair of distinct protuberances separated by a distinct channel (Fig. 6C); tergite VII with pale triangular to semi-circular spot at apex (Fig. 14A) (1 specimen seen missing this spot); distributed in Costa Rica and western Panama; aedeagus as in Fig. 18I–N	*Bolitogyrus bullatus* (Sharp)
18	Pronotum with two setose punctures in the dorsal row (Fig. 7C, arrows)	19
–	Pronotum with three setose punctures in the dorsal row (Fig. 7D, arrows)	22
19	Abdominal segment VIII pale yellow to orange, contrasting with previous, dark brown segments (Fig. 2B); aedeagus as in Fig. 21A–D	*Bolitogyrus cornutus* Brunke, sp. n.
–	Abdominal segment VIII dark brown, not contrasting with previous segments (Fig. 2C)	20
20	Lateral margins of pronotum only weakly convergent anteriad, distinctly less than posteriad, margins nearly straight in anterior half (c.f. Fig. 7D); humeral area of elytra with relatively large pale spot, wider than half the distance between scutellum and humeral angle (Fig. 10D); aedeagus as in Fig. 19K–M	*Bolitogyrus inexspectatus* Brunke, sp. n.
–	Lateral margins of pronotum strongly convergent anteriad, similar to lateral margins in posterior half (Fig. 7C); humeral area of elytra often with pale, crescent-shaped marking, but marking distinctly narrower than half the distance between scutellum and humeral angle (Fig. 10C)	21
21	Occurring in the Tilarán cordillera of Costa Rica (known only from the Monteverde area); aedeagus in parameral view with paramere more strongly asymmetrical, rows of peg setae broader (Fig. 19D, F); median lobe in lateral view slightly recurved dorsad, usually with a sharp angle at this point (Fig. 19E)	*Bolitogyrus tortifolius* Brunke, sp. n.
–	Occurring in the Central and Talamanca Cordilleras of Costa Rica and Panama; aedeagus in parameral view with paramere less strongly asymmetrical, rows of peg setae narrower (Fig. 19G, J); median lobe in lateral view more slender, produced ventrad, with a small subapical tooth (Fig. 19H–I)	*Bolitogyrus pseudotortifolius* Brunke, sp. n.
22	Lateral margins of pronotum only weakly convergent anteriad, distinctly less than posteriad, margins nearly straight in anterior half (Fig. 7D)	23
–	Lateral margins of pronotum strongly convergent anteriad, similar to lateral margins in posterior half (Fig. 7C)	24
23	Genital segment of both sexes bright yellow to orange, contrasting with previous segments (Fig. 14C); distributed in the Guanacaste, Tilarán and Central Cordilleras of Costa Rica; lateral arms of median lobe with apex simple, not flanged and flattened, subapical tooth sharp and not flattened (Fig. 32A); apex of paramere relatively broad, nearly truncate (Fig. 20C–D, F–G)	*Bolitogyrus divisus* Brunke, sp. n.
–	Genital segment of both sexes dark brown, not contrasting with previous segments (Fig. 14D); distributed in the Talamanca Cordillera of Costa Rica and Panama; lateral arms of median lobe with apex flanged and flattened, subapical tooth broader at base and flattened (Fig. 32B); apex of paramere acute, relatively narrow (Fig. 20I, K–L)	*Bolitogyrus falini* Brunke, sp. n.
24	Genital segment in both sexes entirely dark (c.f. Fig. 14D); median lobe divided into two lateral lobes (Fig. 21L); known from Darién province, Panama	*Bolitogyrus cheungi* Brunke, sp. n.
–	Genital segment in both sexes pale, contrasting with tergite VII (c.f. Fig. 14C); median lobe entire but divided by median suture (Fig. 21H); known from Costa Rica and Panama, west of Darién province; males needed to continue	25
25	Male sternite VII distinctly emarginate, with extensive area around emargination flattened and glabrous (Fig. 14E–F); occurring in the Central Cordillera and along the northern edge of the Talamanca Cordillera, in Costa Rica	26
–	Male sternite VII not distinctly emarginate, with relatively small area around middle glabrous and not flattened (c.f. Fig. 13A); occurring in Panama but may occur in Costa Rica in the southern portion of the Talamanca Cordillera; dissected males needed to continue	27
26	Male sternite VII with emargination nearly as deep as wide (Fig. 14E); aedeagus as in Fig. 22G–I	*Bolitogyrus thomasi* Brunke, sp. n.
–	Male sternite VII with emargination distinct but much wider than deep (Fig. 14F); aedeagus as in Fig. 21H–K	*Bolitogyrus bufo* Brunke, sp. n.
27	Median lobe in parameral view evenly converging to apex (Fig. 22A); known from Chiriquí province, Panama; aedeagus as in Fig. 22A–D	*Bolitogyrus gracilis* Brunke, sp. n.
–	Median lobe in parameral view distinctly narrowed in apical third (Fig. 21E; 22E); known from Panama, east of Chiriquí province	28
28	Peg setae fields long, present along entire lateral margin of apical fourth (Fig. 22F); known from Panamá province, Panama	*Bolitogyrus longistellus* Brunke, sp. n.
–	Peg setae fields long, present only as apical cluster (Fig. 21G); known from Veraguas province, Panama	*Bolitogyrus brevistellus* Brunke, sp. n.

### Buphthalmus Group

This species group includes those Neotropical species without the central protuberance on the head and with a median lobe that is sharply constricted in parameral view (e.g., Fig. 15A) (*Bolitogyrus buphthalmus*, *Bolitogyrus costaricensis*, *Bolitogyrus erythrurus*, *Bolitogyrus fulgidus*, *Bolitogyrus pulchrus*, *Bolitogyrus sallei*, *Bolitogyrus salvini*). Although *Bolitogyrus erythrurus* and *Bolitogyrus fulgidus* are known only from females, they are provisionally placed here based on a combination of the first character, habitus and overall similarity of female tergite X to others of this group. This species group is widespread and occurs from Mexico to Panama.

#### Bolitogyrus buphthalmus

Taxon classificationAnimaliaColeopteraStaphylinidae

(Erichson, 1840)

Figs 1A , 4A–B , 15A–D , 23A , 25E–F , 29A (map)

Quedius buphthalmus Erichson, 1840: 534
Cyrtothorax buphthalmus : Sharp 1884: 340
Cyrtothorax cyanescens Sharp, 1884: 341, **syn. n.**
Cyrtothorax buphthalmus : Fauvel 1878: 164 (in key)
Cyrtothorax buphthalmus : Scheerpeltz 1974: 180 (in key)
Bolitogyrus buphthalmus : Navarrete-Heredia et al. 2002 (distributional data)
Bolitogyrus buphthalmus : Márquez 2006: 184 (distributional data)

##### Type locality.

‘Mexico’.

##### Type material.

*Quedius buphthalmus* Erichson, 1840

**Syntype** (♀, BMNH): [specimen minution mounted on card], 3I. [small white label]/ 106 [small pink label]/ France [white label]/ Sharp Coll., 1905.-313 [upside down] / Cyrtothorax Sallei Kraatz, Berl. Ent. Zeitsch., 1858. p 366 pls …[rest illegible]/ Coll. Chevrolat [small label, hand printed in Sharp’s hand]/ Quedius, Buphthalmus Er, [in Chevrolat’s hand, illegible notation underneath], cribricollis Chev. Cat Dej Bolitogyrus, Sallé, Mexico [large green label with confused history] / **Syntype** ♀, *Quedius buphthalmus* Erichson, 1840, Des. Brunke 2013 [red label].

Erichson (1840) stated that his description of this species was based on material from the Chevrolat collection. The entire Staphylinidae portion of this collection was accessed by the British Museum under the care of D. Sharp (Horn et al. 1990a). The single female detailed above (Fig. 4B) is the only specimen of *Bolitogyrus* present in the Chevrolat collection at the BMNH. Although the green label (Fig. 4A) bears the name ‘Quedius buphthalmus Er.’ in Chevrolat’s hand (similar to that in Horn et al. 1990b) and the label ‘coll. Chevrolat’ (in Sharp’s hand) confirm that this specimen belonged to Chevrolat, Erichson’s handwriting is missing from any labels currently on the specimen. The multiple names and entries on the green label (Fig. 4A) demonstrates the original confusion surrounding the type species of the genus and an unavailable name (‘*Bolitogyrus cribripennis*’). For details see ‘Taxonomic History’ above. However, the text ‘*Quedius buphthalmus* Er. Mexico’ appears to be the original entry of the label. Erichson (1840) did not provide any evidence that only one specimen was included in the type series of *Quedius buphthalmus* and so this specimen is interpreted as a syntype of that species. The Latin expression ‘insectum singulare’ at the beginning of his description means ‘unique insect’ and refers to the habitus of the specimen, unique among described taxa. This interpretation is consistent with the style of Erichson (1840), who typically commented on the habitus of his species at the beginning of his descriptions. This specimen is consistent with the original description and the concept for this species given in the present work. *Bolitogyrus buphthalmus* is interpreted here as the most common species of the *buphthalmus* group occurring in Mexico. We have no doubt that this was the species described by Erichson as the female syntype has completely dark coxae, which would exclude the similar *Bolitogyrus sallei* Kraatz, and the abdominal microsculpture is extremely fine, excluding *Bolitogyrus newtoni* Brunke, sp. n.

#### Cyrtothorax sallei

Taxon classificationAnimaliaColeopteraStaphylinidae

Kraatz, 1858 (ex. parte)

##### Type locality.

‘Mexico’.

##### Paralectotypes

(specimens 3, 4 and 5 are *Bolitogyrus buphthalmus*, 2 ♂, 1 ♀, DEI): Mexico [blue label with script] / coll. Kraatz [white label, printed] / SYNTYPUS [red label, printed] / PARALECTOTYPE, *Cyrtothorax sallei* Kraatz, 1858, det. A. Brunke 2013 [yellow printed label] / *Bolitogyrus buphthalmus* (Erichson) Det. A. Brunke 2012 [white printed label].

For details, see under *Bolitogyrus sallei*.

#### Cyrtothorax cyanescens

Taxon classificationAnimaliaColeopteraStaphylinidae

Sharp, 1884 syn. n.

##### Type locality.

[Finca] Capetillo, Sacatepéquez, Guatemala (approximately 14.48, -90.80).

**Lectotype** (♂, here designated, BMNH): ‘♂, Cyrtothorax cyanescens, Capetillo, Guat.[emala], Champion’ [Sharp’s handwriting on card] / ‘Capetillo, Guatemala, Champion’ [printed label] / ‘B.C.A. Col. I.2., Cyrtothorax cyanescens, Sharp’ [printed label] / ‘Sharp Coll. 1905.-313’ [printed label] / ‘Syntype’ [beige disc with blue border] / LECTOTYPE ♂, *Cyrtothorax cyanescens* Sharp, 1884, des. A. Brunke 2013 [red printed label] / *Bolitogyrus buphthalmus* (Erichson) Det. A. Brunke 2012 [white printed label].

**Paralectotypes** (9 total: 5 ♂, 1 ♀ examined, FMNH (2), BMNH (7)): same information as Lectotype, **Paralectotype** *Cyrtothorax cyanescens* Sharp, 1884, det. A. Brunke 2013 [yellow printed label] / *Bolitogyrus buphthalmus* (Erichson) det. A. Brunke 2012 [white printed label], 1 ♂ (BMNH); same as previous, except with: ‘sp. figured’ [printed label] / ‘G’ [square handwritten label], 1 ♂ (BMNH); ‘Capetillo, Guatemala, Champion’ / ‘B.C.A. Col. I.2., Cyrtothorax cyanescens, Sharp’ / ‘Sharp Coll. 1905.-313’ / ‘Syntype’ [beige disc with blue border] / **Paralectotype** *Cyrtothorax cyanescens* Sharp, 1884, det. A. Brunke 2013 [yellow printed label] / *Bolitogyrus buphthalmus* (Erichson) det. A. Brunke 2012 [white printed label] 2 ♂ (BMNH); ‘♂, Capetillo, Guat.[emala], Champion’ [Sharp’s handwriting on card] / ‘Capetillo, Guatemala, Champion’ / ‘B.C.A. Col. I.2., Cyrtothorax cyanescens, Sharp’ / ‘Chicago Nat. Hist. Mus. (ex. D. Sharp Colln. by exchange with Brit. Mus. Nat. Hist.)’ / **Paralectotype** *Cyrtothorax cyanescens* Sharp, 1884, det. A. Brunke 2013 [yellow printed label] / *Bolitogyrus buphthalmus* (Erichson) det. A. Brunke 2012 [white printed label], 1 ♂, 1 ♀ (FMNH).

Sharp (1884) described this species from southern Guatemala based on a series of 10 specimens, all possessing a striking deep purple metallic reflection on the forebody. Sharp (l884) also reported a relatively large body size and greater extent of dark markings on the legs. Study of the syntype series and several additional deep purple specimens from the same area of Guatemala revealed that both size and leg banding are variable within this group of specimens and that this variation falls within that of *Bolitogyrus buphthalmus*. More importantly, the pattern of peg setae, the shape of the paramere and the shape of the median lobe in both lateral and parameral view of the syntypes of *Bolitogyrus cyanescens* are consistent with *Bolitogyrus buphthalmus*, and *Bolitogyrus cyanescens* is therefore synonymized with it. To promote stability of nomenclature, a male syntype was selected and is here designated as a Lectotype of *Cyrtothorax cyanescens* Sharp, 1884.

##### Other material.

**MEXICO:** ‘Sharp Coll. 1905.-313.’, 1 ♂ (BMNH); ‘Mex.’, 1 ♂ (FMNH), 1 ♂ (BMNH); ‘Mexico’, Coll. C. Felsche, Geschenk 1907, 1 ♂ (DEI); ‘Mexico’, 1 ♂ (NHRS). *Puebla*: Xicotepec de Juárez, Hidroeléctrica Patla, 487m, selva mediana [=tropical forest], en tronco [=in log], 6.III.2002, J. Asiain y J. Márquez, 1 ♂, 1 ♀ (MZFC). *Veracruz*: ‘Cordova’ [=Córdoba], “Sharp Coll. 1905.-313.”, Sallé, 2 ♂ (BMNH); same as previous except: Höge, 1 ♂ (FMNH); Jalapa, La Herrandura, bosque mesófilo de Montaña [=mountain cloud forest], ex. tronco caído podrido [=rotten fallen log], 21.II.1999, Q. Santiago and J. Márquez, 1 ♂ (MZFC); ‘Jalappa’ [=Jalapa], 1 ♂ (NMW); ‘Toxpam’ [=Tuxpan, IN ERROR], Sallé, 1 ♂ (BMNH). **GUATEMALA:** *Chimaltenango*: Yepocapa, La Jolla Grande (Finca Monserrat), NW slope of Volcan Fuego, 5700 ft., under log, 3.V.1948, 1 ♂, 1 ♀ (FMNH). *Escuintla*: Zapote, El Zapote, 4000 ft., 7.VII.1948, ex. fungi, 1 ♂ (ZMUC). *Quetzaltenango*: ‘Cerro Zunil’, ‘4-5000 ft.’, Champion, ‘Sharp Coll. 1905.-313.’, 1 ♂ (BMNH); 14.4 km SW of Zunil, 1340m, 18.VI.1993, J. Ashe, R. Brooks, ex. crustose fungus on log, SM0037986 and SM0037987, 2 ♂ (SEMC). **COSTA RICA:** *Cartago*: Turrialba, 800m, 1 ♂ (ZMHB) [probably in error].

##### Diagnosis.

Head without central protuberance; antenna and legs distinctly bicolored (Fig. 5A, 11G); genital and abdominal segments VIII not distinctly paler than previous segments; abdominal sternites with fine transverse microsculpture, interspaces about as wide as lines (Fig. 13D); female procoxa completely dark; median lobe projecting ventrad, not recurved (Fig. 15B); paramere with convergent arms; paramere with fields of peg setae narrow and angulate at base of arms (Fig. 15C–D); female tergite X with raised disc converging strongly toward apex (Fig. 25E–F), strongly pigmented area sharply incised at apicolateral margin; female secondary gonocoxite not distinctly swollen at base.

##### Redescription.

Measurements ♂ (n=5): HW/HL 1.46–1.67; PW/PL 1.50–1.69; EW/EL 1.16–1.41; ESut/PL 0.69–0.79; PW/HW 1.14–1.17; forebody length 4.4–5.2 mm.

Measurements ♀ (n=3): HW/HL 1.47–1.50; PW/PL 1.32–1.41; EW/EL 1.24–1.25; ESut/PL 0.73–0.77; PW/HW 1.14–1.19; forebody length 5.1–5.5 mm.

Coloration: Body black, head and pronotum usually, elytra always with faint bronze to greenish-bronze (Mexico) or dark purple to dark bluish-purple (Guatemala) metallic reflection; lateral outline of pronotum paler in some specimens; apical fifth of abdominal tergites slightly paler in some specimens; abdominal segment VIII and genital segment entirely dark; maxillary and labial palpi pale to dark reddish-brown; antennomeres 1-5 reddish-orange, with some darkened areas, antennomeres 6-10 dark brown, contrasting with previous, apical segment usually distinctly paler, yellow to orange, occasionally only slightly paler or dark; legs bicolored: forecoxa yellow, often with some basal darkening (males) or completely dark brown (females), mid and hind coxa dark brown, femur yellow and distinctly darkened apically, tibia brown to dark brown, often with medial face lighter, tarsus light brown.

Head distinctly transverse, without central protuberance or pair of posterior protuberances, without wide impunctate areas.

Pronotum distinctly transverse, especially in some males; impunctate on disc, with one puncture in dorsal row (i.e., only marginal puncture), lateral portion explanate, and irregularly, shallowly punctate, these punctures without setae and sometimes running together; with protuberance, more pronounced in lateral view in males than in females (Fig. 7E–F). Elytra weakly transverse, shorter than pronotum at middle, surface moderately to strongly uneven (Figs 9E, 10B); surface without microsculpture, with coarse irregularly spaced punctures.

Abdomen with sparse, dark brown pubescence; disc of tergites III–VI impunctate at middle; sternites III–V with basal transverse line sharply projected posteriad at middle; apical half of abdominal sternites with dense, fine, transverse microsculpture, interspaces about as wide as lines.

Median lobe in lateral view projected ventrad (Fig. 15B); apical portion of median lobe in parameral view sharply constricted to form narrow base and dilated midway to a variable degree (Fig. 15A); paramere divided into two lobes, convergent apicad (Fig. 15A), unevenly convex on inner surface; peg setae fields acute at base of parameral lobe and not continuing toward base (Fig. 15C–D). Male sternite VII without distinct modifications. Male sternite VIII weakly emarginate, with triangular asetose area medially (Fig. 13A), transverse basal line complete medially; male tergite X with apex entire, rounded and weakly acute; male sternite IX distinctly emarginate, base strongly asymmetrical (Fig. 23A).

Female tergite X shield-shaped, apex weakly to strongly acute and produced, with many long setae, strongly pigmented area strongly incised at apicolateral margin (Fig. 25E–F).

##### Distribution.

Figure 29A. *Bolitogyrus buphthalmus* is known from Puebla and Veracruz in Mexico, and in southern Guatemala. It is likely that the distribution in Mexico extends much further south but more collecting is needed in Oaxaca and Chiapas. The record for Oaxaca given by Navarrette-Heredia et al. (2002) based on an FMNH specimen corresponds to *Bolitogyrus newtoni*. Scheerpeltz (1974) included a record of *Bolitogyrus buphthalmus* from El Salvador but this is based on a misidentified specimen of *Bolitogyrus bechyneorum*. One male was seen from Turrialba, Costa Rica (ZMHB) but this isolated record is treated as doubtful as all other Costa Rican specimens seen were *Bolitogyrus costaricensis*. The label data from a specimen in Sharp’s collection (BMNH) from ‘Tuxpan’ is considered to be inaccurate as this city is located near the coast at approximately sea level, distinctly outside this species’ elevational range.

##### Bionomics.

*Bolitogyrus buphthalmus* has been collected in tropical and cloud forests from 487–1737m, in association with fungusy or rotten logs. Specimens have been collected in February to March and May to July.

##### Comments.

*Bolitogyrus buphthalmus* is most similar to and is only reliably separated from *Bolitogyrus costaricensis* by characters on the male genitalia. The pigmented area of female tergite X on the available, male-associated females is distinctly more incised at the apicolateral margin than in *Bolitogyrus costaricensis*. All confirmed specimens of *Bolitogyrus buphthalmus* south of Mexico (in Guatemala) are deep purple on the forebody, indicating that this population may be partially or entirely isolated from the population in Mexico. One male seen from Guatemala (Cerro Zunil) is metallic bronze-green and possesses peg setae fields characteristic of *Bolitogyrus buphthalmus* but the median lobe in lateral view is recurved dorsad as is characteristic of *Bolitogyrus costaricensis*. This specimen is difficult to assign to a species but may represent a hybrid between *Bolitogyrus buphthalmus* and *Bolitogyrus costaricensis* within the overlap of these two species’ distributions. Alternatively, the shape of the parameral peg setae fields may be shown to be unreliable with the study of additional material; this specimen would then be assigned to *Bolitogyrus costaricensis* based on the median lobe. All other males studied from Guatemala were unambiguously identified as either species.

#### Bolitogyrus costaricensis

Taxon classificationAnimaliaColeopteraStaphylinidae

(Wendeler, 1927)

Figs 5A , 6B , 7E–F , 11A , 12C , 13A, D , 15E–H , 23B , 25A–D , 29A–B (maps)

Cyrtothorax costaricensis Wendeler, 1927: 8
Cyrtothorax costaricensis : Scheerpeltz 1974: 181 (in key)
Cyrtothorax nevermanni Scheerpeltz, 1974: 190, **syn. n.**

##### Type locality.

Turrialba Volcano, Cartago, Costa Rica

##### Type material.

*Cyrtothorax costaricensis* Wendeler, 1927

##### Holotype

(♀, ZMHB): ZENTR. AMERIKA, Costarica, Vukan Turialba, 10 km NÖ [northeast] v.[ulkan] Irazu [beige printed label]/Cyrtothorax costaricensis, Wendeler [white label, name in script, author in print]/costaricensis [large green label, folded, hand printed]/Holotypus [red label, printed]/ Bolitogyrus, costaricensis, Det. Chatzimanolis 2010 [white label, hand printed] / Holotype ♀, *Cyrtothorax costaricensis* Wendeler, 1927, det. A. Brunke 2013 [red printed label].

The specimen located in ZMHB is interpreted as the holotype as Wendeler (1927) stated that only one specimen was seen. The female holotype possesses blue-purple metallic reflections on the elytra rather than deep purple, as stated by Wendeler (1927), a dark abdominal apex, fine abdominal microsculpture, a dark apical band on the mesofemur, a characteristically shaped tergite X and entirely dark forecoxae. Therefore, it is morphologically consistent with a species with known male sexual characters that is distributed from Guatemala to Costa Rica, here considered distinct from *Bolitogyrus buphthalmus* based on features of the male genitalia.

#### Cyrtothorax nevermanni

Taxon classificationAnimaliaColeopteraStaphylinidae

Scheerpeltz, 1974 syn. n.

##### Holotype

(♀, NMW): Costa Rica, Carpintera, leg. Nevermann [white hand printed label]/ ♀ [white printed card]/ Cyrtothorax Nevermanni, n. sp./ Allotypus [red printed label]/ Nevermanni, Scheerp. [green hand printed card]/ ex. coll. Scheerpeltz [blue printed label]/ TYPUS, Cyrtothorax, nevermanni, O. Scheerpeltz [red hand printed label]/ Holotype ♀, *Cyrtothorax nevermanni* Scheerpeltz, 1974, det. A. Brunke 2013 [red printed label] / Bolitogyrus costaricensis (Wendeler) Det. A. Brunke 2012 [white printed label].

The single specimen located in NMW is interpreted as the holotype as Scheerpeltz (1974) stated that only one specimen was seen. Although this holotype is also female, it can be associated with the same species concept as the type of *Bolitogyrus costaricensis* for the same reasons (above). Therefore, *Bolitogyrus nevermanni* (Scheerpeltz) becomes a synonym of *Bolitogyrus costaricensis* (Wendeler).

##### Other material.

**GUATEMALA:** *Baja Verapaz*, Sinanjá, Champion, “Sharp Coll.1905.-313”, 1 ♂ (BMNH). *San Marcos*: 2 km E de Tajomulco [=Tajumulco], Bosque mesófilo Perturbado [=disturbed cloud forest], 2062m, 15°04'99"N, 91°55'563W, en hongos de reprisa [=in shelf fungus], 23.IX.1999, J. Márquez, 1 ♂ (MZFC). **HONDURAS:** *Comayagua*: 13 km SE El Mochito, Sta. B, 22.VII.1977, ‘CW & L.’, O’Brien & Marshall, 1 ♂ (FMNH). *El Paraiso*: 9.4 km SE Zamaranom & 9.4 km SE Galeras, ‘Los Lavenderos’, 13°24'N, 86°55'W, 1450m, 11.VI.1994, ex. crustose fungi under logs, Ashe, Brooks, SM0037968, SM0037969, SM0037970, SM0037971, 1 ♂, 3 ♀ (SEMC). *Francisco Morazán*: 10.4 km W Zamorano, Cerro Uyuca, 14°02'N, 87°04'W, 1500m, 27.VI.1994, ex. crustose fungi under log, Ashe, Brooks, SM0037967, 1 ♂ (SEMC); 7.6 km N Guaimaca, 14°36'N, 86°49'W, 820m, 26.VI.1994, crustose fungi under logs, J. Ashe, R. Brooks, SM0037975, SM0037976, SM0037977, SM0037978, SM0037979, SM0037980, SM0037981, SM0037982, 6 ♂, 2 ♀ (SEMC); *Lempira*: 5.9 km SW Gracias, Mt. Celaque, 14°33'N, 88°38'W, 1250m, 17.VI.1994, on/under fungusy log, SM0037965, SM0037966, 2 ♂ (SEMC); 13.1 km NE & 7.3 km E Gracias, Mt. Puca, 14°41'N, 88°31'W, 1320m, 18.VI.1994, crustose fungi under logs, J. Ashe, R. Brooks, SM0037973 and SM0037974, 1 ♂, 1 ♀ (SEMC); same as previous except: under wood slabs, SM0037963, SM0037962, 1 ♂, 1 ♀ (SEMC). *Santa Barbara*: Santa Barbara, Mt. Santa Barbara, 11.5 km S & 5.6 km W Pefia Blanca, 14°57'N, 88°05'W’, 1800m, 20.VI.1994, beating stick pile, R. Brooks, J. Ashe, SM0037972, 1 ♂ (SEMC). **NICARAGUA:** *Grenada*: Res. Nat. Volcán Mombacho, Bmm, 11°50'25N, 85°58'593W, 1039 m, ex. tronco podridos [=rotten logs], 2.II.2000, J. Márquez, 1 ♂, 1 ♀ (MZFC); same as previous except: 3.II.2000, 1 ♂ (MZFC); same as previous except: 4.II.2000, 2 ♂ (MZFC); Res. Nat. Volcan Mombacho, entrance road, 11°50.05'N, 85°58.83'W, 1060m, flight intercept trap, 1 to 5.VI.2002, R. Brooks, Z. Falin, S. Chatzimanolis, SM0575457, SM0575458, SM0575459, 2 ♂, 1 ♀ (SEMC); same as previous except: 800-1000 m, pyrethrum fogging fungusy logs, NIC1BFC02 145, SM0413171, 1 ♂ (SEMC); Res. Nat. Volcan Mombacho, station, 11°50.05'N, 85°58.83'W, 1150m, mercury vapor/UV light, 2.VI.2002, R. Brooks, Z. Falin, S. Chatzimanolis, SM0533433, 1 ♂ (SEMC); Res. Nat. Volcan Mombacho, 11°50.05'N, 85°58.83'W, 1150m, pyrethrum fogging fungusy logs, 2.VI.2002, R. Brooks, Z. Falin, S. Chatzimanolis, SM0533722, SM0413244, SM0413245, SM0413246, 3 ♂, 1 ♀ (SEMC); same as previous except: 1200m, ex. cut lumber, SM0413175, SM0413176, SM0413177, SM0413178, 4 ♂ (SEMC); same as previous except: pyrethrum fogging fungusy logs, SM0413179, SM0413180, 1 ♂, 1 ♀ (SEMC, ZMUC); Res. Nat. Volcan Mombacho, 11°50.05'N, 85°58.83'W, 1150 m, pyrethrum fogging fungusy logs, 3.VI.2002, R. Brooks, Z. Falin, S. Chatzimanolis, SM0413225, SM0413226, SM0413228, SM0413230, SM0413231, SM0413233, SM0413235, SM0413236, SM0413237, SM0413238, SM0413239, SM0413240, SM0413241, 7 ♂, 6 ♀ (SEMC); same as previous except: 4.VI.2002, SM0413264, SM0413265, SM0413266, SM0413267, SM0413268, SM0413269, SM0413270, SM0413271, SM0413272, 5 ♂, 4 ♀ (SEMC); same as previous except: 5.VI.2002, SM0555363, SM0555364, SM0555365, SM0555366, 4 ♂ (SEMC, UTCI, ZMUC). *Jinotega*: 16 km N Matagalpa, Matagalpa-Jinotega road, 13°02.70'N, 85°56.10'W, 1385m, 22.V.2002, pyrethrum fogging fungusy logs, R. Brooks, Z. Falin, S. Chatzimanolis, SM0413166, SM0413167, SM0413168, SM0413169, 3 ♂, 1 ♀ (SEMC). *Matagalpa*: 6 km N Matagalpa, Selva Negra Hotel, 12°59.99'N, 85°54.53'W, 1240m, flight intercept trap, Bavaria trail, 18 to 21.V.2002, S. Chatzimanolis, SM0413258, 1 ♂ (SEMC); same as previous except: 1300m, Cody trail, R. Brooks, Z. Falin, S. Chatzimanolis, SM0555396, SM0555397, 2 ♂ (SEMC); 6 km N Matagalpa, Selva Negra, 12°59.9'N, 85°54.6'W, 1250m, flight intercept trap, 18 to 22.V.2002, S. Peck, SM0560604, SM0560605, SM0560610, SM0560623, SM0560627, SM0560630, SM0560632, SM0560635, SM0560636, SM0560638, SM0560639, SM0560645, SM0560647, SM0560652, 8 ♂ 5 ♀ (SEMC); 6 km N Matagalpa, Selva Negra Hotel, 12°59.99'N, 85°54.53'W, 1250m, 19.V.2002, pyrethrum fogging fungusy logs, R. Brooks, Z. Falin, S. Chatzimanolis, SM0575440, SM0575442, SM0575441, SM0575439, SM0558105, SM0558113, 3 ♂ 3 ♀ (SEMC); same as previous except: 1350m, crustose polypore, SM0540977, SM0540978, SM0540979, SM0540980, SM0540981, SM0540982, SM0541459, 1 ♂ 6 ♀ (SEMC); 6 km N Matagalpa, Selva Negra Hotel, 12°59.99'N, 85°54.53'W, 1520m, 20.V.2002, pyrethrum fogging fungusy logs, R. Brooks, Z. Falin, S. Chatzimanolis, SM0542449, SM0542488, SM0575433, 2 ♂, 1 ♀ (SEMC); same as previous except: 1350m, ex. crustose polypore, SM0540939, SM0540940, SM05409941, SM0540942, SM0540943, SM0540944, SM540946, SM0540949, SM0540950, SM0540951, SM0540952, SM0540953, SM0540954, SM0540955, SM0540956, SM0540957, SM0540958, SM0540960 SM0540963, SM540964, 13 ♂, 7 ♀ (SEMC); same as previous except: 21.V.2002, SM0541899, SM0541903, SM0543437, SM0575530, SM0575531, SM0575532, SM0575533, SM0575534, SM0575535, SM0575536, SM0575537, SM0575538, SM0575539, SM0575540, SM0575541, SM0575542, SM0575543, SM0575544, SM0575545, SM0575546, SM0575547, SM0575548, SM0575549, SM0575550, SM0575551, SM0575552, SM0575553, SM0575554, SM0575555, SM0575556, 19 ♂, 11 ♀ (SEMC); 6 km N Matagalpa, Selva Negra Hotel, 12°59.99'N, 85°54.53'W, 1350m, 21.V.2002, ex. small white bracket fungi, R. Brooks, Z. Falin, S. Chatzimanolis, SM0558419, 1 ♂ (SEMC); same as previous except: ex. cut lumber, SM0575557, SM0575558, SM0575560, SM0575563, 2 ♂, 2 ♀ (SEMC); same as previous except: on Xylariaceae, SM0575488, SM0575489, SM0575490, SM0575491, SM0575492, SM0575493, SM0575494, SM0575495, SM0575496, SM0575497, 8 ♂, 2 ♀ (SEMC). **COSTA RICA:** [locality illegible], 18.IV.1938, 1 ♂ (FMNH); country record only, unter loser Rinde [under loose bark], 9.XI.1936, 1 ♀ (USNM). *Cartago*: Carpintera, 16.VI.1940, 2 ♂, 2 ♀ (FMNH); Tres Ríos, 23.VI.1940, 4 ♂ (FMNH); Cerro Carpintera, Iztaru Scout Camp, 1670m, ex. fog dry log, 21.IV.2000, CR-1090, P.N. Thomas, 3 ♂, 4 ♀ (PTC); Santa Cruz de Turrialba, ex. sub bark log in pasture near road, 26.VIII.1994, P.N. Thomas, CR-432, 1 ♀ (PTC). *Guanacaste*: P. N. Guanacaste, Lado SO [=southwest side] vol. Cacao, Est. Cacao, 1000-1400 m, 21 to 29.V.1992, F. A. Quesada, CR1000378852, 1 ♂ (INBIO); same as previous except: R. Blanco & C. Chavez, 1000086948, 1 ♂ (INBIO); P.N. Guanacaste, Lado O [=west side] Volcan Orosi, Estac. Maritza, 600 m, malaise, 1989, CR1000037210, 1 ♂ (INBIO); same as previous except: 1992, CR1000377704, 1 ♂ (INBIO); Rincon de la Vieja, Pailas Station, 740-790 m, ex. fog asst. logs, 1.XII.2000, CR-1147, 1 ♂ (PTC). *Limón*: Parismina, M. Valerio coll., 1 ♀ (USNM) [LOC. IN ERROR]. *Puntarenas*: Monte Verde, 1250m, 7.V.1989, J. Ashe, R. Brooks, R. Leschen, SM0037961, 1 ♂ (SEMC); Monte Verde, 1400m, ex. flight intercept trap, 21.V.1989, J. Ashe, R. Brooks, R. Leschen, SM0037960, 1 ♂ (SEMC). *San Jose*: La Caja, VIII.1938, 2 ♂ (FMNH); San Jose, 1935, 1 ♂ (FMNH), same except 1937, 1 ♀ (USNM); Río Virilla, 15.V.1941, Bierig, 1 ♂ (FMNH); Rincon de Santa Maria South, 1650 m, ex. rotten stump, 17.XI.2003, CR-1481, P.N. Thomas, 3 ♂ (PTC); San Francisco de Leon Cortes, 1780 m, ex. fog dry logs, 3.IV.2000, CR-1119, P.N. Thomas, 3 ♂, 4 ♀ (PTC); San Marcos [in error] [San Lorenzo] de Tarrazú, Zapotal, 1500 m, ex. rotten oak logs, 4.I.1996, sample #65, P.N. Thomas, 3 ♂, 3 ♀ (PTC); same except: ex. logs with moss, 18.XII.1993, sample #267, 2 ♂, 3 ♀ (PTC); San Marcos, San Lorenzo de Tarrazú, Cerro Zapotal, 1500 m, ex. dried mushrooms, pan, 16.XII.1993, CR-255, P.N. Thomas, 1 ♂ (PTC); same except: ex. encino and poro log, 23.VIII.1994, CR-417, 1 ♂ (PTC).

##### Diagnosis.

Head without central protuberance; antenna and legs distinctly bicolored (Fig. 5A; 11G); genital and abdominal segments VIII not distinctly paler than previous segments; abdominal sternites with fine transverse microsculpture, interspaces about as wide as lines (Fig. 13D); female procoxa completely dark; median lobe recurved at apex (Fig. 15F); paramere with convergent arms; paramere with fields of peg setae wide and circular at base of arms (Fig. 15G–H); female tergite X with raised disc converging strongly toward apex, strongly pigmented area not or weakly incised at apicolateral margin (Fig. 25A–D); female secondary gonocoxite not distinctly swollen at base.

##### Redescription.

Measurements ♂ (n=5): HW/HL 1.54–1.62; PW/PL 1.29–1.67; EW/EL 1.24–1.26; ESut/PL 0.68–0.80; PW/HW 1.10–1.25; forebody length 4.1–4.9 mm.

Measurements ♀ (n=5): HW/HL 1.50–1.67; PW/PL 1.35–1.47; EW/EL 1.21–1.30; ESut/PL 0.75–0.78; forebody length - 4.5–5.1 mm.

Morphology as in *Bolitogyrus buphthalmus*, differing only in the following: metallic reflections of body bronze to greenish or grayish-bronze or violet to violet-blue; median lobe in lateral view recurved dorsad (Fig. 15F); peg setae fields broad and circular at base of parameral lobe (Fig. 15G–H); male sternite IX with apical emargination deeper (Fig. 23B); female tergite X with strongly pigmented area not or weakly incised lateroapically (Fig. 25A–D).

##### Distribution.

Figure 29A–B. *Bolitogyrus costaricensis* is known from Guatemala, Honduras, Nicaragua and Costa Rica, west to the northern part of the Talamanca Cordillera. Two females from Belize were seen that might belong to this species or *Bolitogyrus buphthalmus*. The specimen from Limón province in Costa Rica was almost certainly collected from elsewhere as Parismina is a city located on the Caribbean coast, situated at sea level, which is distinctly outside the elevational range of this species.

##### Bionomics.

Most specimens of *Bolitogyrus costaricensis* have been collected by pyrethrum fogging of fungusy logs, while others were collected from bracket fungi, dried mushrooms, small crustose polypores, fungi in the family Xylariaceae, various woody debris and in FIT or Malaise traps; one slightly teneral specimen came to light. *Bolitogyrus costaricensis* has been collected in forests at elevations ranging from 600-2062 m in every month of the year.

##### Comments.

*Bolitogyrus costaricensis* is most similar to *Bolitogyrus buphthalmus* and can only be separated from it by characters on the aedeagus (see above).

#### Bolitogyrus erythrurus

Taxon classificationAnimaliaColeopteraStaphylinidae

(Kraatz, 1858)

Figs 25I–K , 29B (map)

Cyrtothorax erythrurus Kraatz, 1858: 368
Cyrtothorax erythrurus : Scheerpeltz 1974: 182 (in key)

##### Type locality.

“Nova Grenada” [=Panama/Columbia]

##### Type material.

*Cyrtothorax erythrurus* Kraatz, 1858.

**Syntype** (♀, DEI): Nov. Gren. [green label, script]/ Coll. Kraatz [white label, printed]/ erythrurus [green label, script]/ Syntypus [red label, printed] / Syntype ♀, *Cyrtothorax erythrurus* Kraatz, 1858, det A. Brunke 2013 [red printed label].

As Kraatz (1858) did not provide evidence that only one specimen was studied, the only specimen located in DEI with Kraatz’s labels is interpreted as a syntype.

##### Other material.

**COSTA RICA:** *Puntarenas*: Las Mellizas, Térraba Valley, 2 km NW of Triplets [from INBIO database], Fca. Cafrosa, P.N. La Amistad, 1300m, 8.8915, -82.7929 [from INBIO database], V-1990, M. Ramirez & G. Mora, INBIO 000259065, 1 ♀ (INBIO). **PANAMA:** *Chiriquí*: 20 km N of Gualaca, Finca La Suiza, 1350m, 8°39'N, 82°12'W, 10-VI-1995, ex. fogging fungusy log, J. Ashe & R. Brooks, SM0037994, 1 ♀ (SEMC); Boquete, 15.VI.1978, G.J. Umphrey, 1 ♀ (DEBU).

##### Diagnosis.

Head without central protuberance; antenna distinctly bicolored (Fig. 5A); genital segment, abdominal segment VIII and narrow apex of VII distinctly paler than previous segments; female procoxa completely dark; female tergite X with elongate, raised area, that is widest near base and distinctly converges toward apex, outline of female tergite X widest near middle, strongly pigmented area not as strongly incised at apicolateral margin as in *Bolitogyrus buphthalmus* (Fig. 25I–K); female secondary gonocoxite not distinctly swollen at base.

##### Redescription.

Measurements ♀ (n=4): HW/HL 1.47–1.61; PW/PL 1.41–1.50; EW/EL 1.24–1.32; ESut/PL 0.79–0.80; PW/HW 1.14; forebody length 4.9–5.3 mm.

Similar to *Bolitogyrus buphthalmus* and differing only in the following: legs bicolored or not (legs of syntype not distinctly bicolored, possibly discolored due to killing agent or age); with metallic reflections as in *Bolitogyrus costaricensis*; narrow apex of segment VII, segment VIII and genital segment yellow to orange, distinctly contrasting with previous segment; female tergite X with raised area more elongate and widest near base; outline of female tergite X widest near middle.

##### Distribution.

Figure 29B. *Bolitogyrus erythrurus* is known from the Talamanca Mountain Range: southern Puntarenas in Costa Rica and the adjacent province of Chiriquí in Panama.

##### Bionomics.

Specimens were collected from May-June at elevations ranging from 1300–1350m. One specimen was pyrethrum-fogged from a fungusy log.

##### Comments.

The female syntype and three other females studied differ from others of the Buphthalmus Group by the pale abdominal apex, bicolored antennae and overall shape of female tergite X. The shape of female tergite X is roughly similar to that of *Bolitogyrus buphthalmus*, *Bolitogyrus costaricensis* and *Bolitogyrus sallei* but some slight differences were observed (see Description). Previous authors, including Kraatz, apparently overlooked the pale abdominal apex of the holotype, which was partially retracted into the preceding segments and less noticeable due to the teneral coloration of the remaining abdomen. This dark red coloration on abdominal segments V–VII described by Kraatz (1858) is not significant, as this occurs variably in other species of the Buphthalmus Group, possibly due to incomplete sclerotization after eclosion. *Bolitogyrus erythrurus* is most similar to *Bolitogyrus costaricensis* but is allopatric with it and is tentatively retained as a valid species, pending the availability of similarly colored males from the Talamanca Mountains.

#### Bolitogyrus fulgidus

Taxon classificationAnimaliaColeopteraStaphylinidae

(Sharp, 1884)

Figs 1B , 5B , 7B , 9B , 10A , 11D, F , 12D–E , 26E–F , 27H , 29C (map)

Cyrtothorax fulgidus Sharp, 1884: 341
Cyrtothorax fulgidus : Scheerpeltz 1974: 182 (in key)

##### Type locality.

Chontales, Nicaragua.

##### Type material.

*Cyrtothorax fulgidus* Sharp, 1884. **Holotype** (♀, BMNH):“Cyrtothorax fulgidus, Type D.S., Chontales Nicaragua, Janson” [written on card in Sharp’s hand, with specimen]/ Chontales, Nicaragua, Janson [printed label]/ B.C.A. Col. I.2., Cyrtothorax, fulgidus, Sharp. [printed label]/ Type [printed on disc with red border]/ Sharp Coll., 1905.-313. [printed label] / Holotype ♀, *Cyrtothorax fulgidus* Sharp (1884), det. A. Brunke 2013 [red printed label].

The single, female specimen located in the BMNH is interpreted as the holotype as Sharp (1884) stated that only one specimen was seen. The holotype differs from all other species of the genus by the beveled apex of female tergite X (Fig. 26E–F).

##### Other material.

**COSTA RICA:** *Alajuela*: Estación San Ramón Oeste, [10.8832, -85.4135 retrieved from INBIO specimen database] 620m, 3 to 19.IV.1994, F. Quesada, 001777049, ♀ (INBIO). *Heredia*: La Selva Biological Station, ‘10.26°N, 84.01°W’ [10°26'0” N, 84°1'0"W], 50-150m, ex. Malaise, 15.XII.1993 to 3.I.1994, ALAS, M/05/308, 1 ♀ (PTC); same except: 1.VI to 1.VII.1993, M/06/151, 1 ♀ (PTC). *Puntarenas*: Monteverde, 10.30, -84.80 [retrieved from SEMC database], 1550m, 15.X.2000, R. M. Timm, SM0694452, 1 ♀ (SEMC).

##### Diagnosis.

Head without central protuberance; antenna distinctly bicolored (Fig. 5A); genital and abdominal segments VIII distinctly paler than previous segments; abdominal sternites with fine transverse microsculpture, interspaces about as wide as lines (Fig. 13D); female procoxa entirely pale not contrasting with profemur; female tergite X with beveled apex (Fig. 26E–F); female gonocoxite distinctly swollen at base (Fig. 27H).

##### Redescription.

Measurements ♀ (n=5): HW/HL 1.48–1.56; PW/PL 1.32–1.43; EW/EL 1.24–1.31; ESut/PL 0.66–0.73 (one specimen = 0.80); PW/HW 1.14–1.19; forebody length 5.2–5.5 mm.

Similar to *Bolitogyrus buphthalmus* and differing only in the following: head with metallic green to greenish-bronze reflections; pronotum varying from bright orange-red to dark reddish brown with a metallic greenish-bronze reflection and broadly reddish margins; elytra with metallic blue to greenish-bronze reflection, epipleuron and extreme base of elytra partly reddish; abdominal segments III–V reddish, with variable amounts of darkening both dorsally and ventrally; entire abdominal segment VI and base of VII dark brown, apex of VII onward, including genital segment, yellow to orange, distinctly paler than previous segments; antennomeres XI or X–XI paler than preceding segments; legs not distinctly bicolored: coxa light reddish-brown and remainder of legs either equally pale or gradually darkened to dark brown, toward apex of tibia; median frontal impression weak; head with impunctate area in middle of disc; last segment of labial palpus distinctly setose, setae more numerous and longer than in *Bolitogyrus buphthalmus*; pronotum with pair of depressions but without distinct protuberance in lateral view; surface of elytra relatively even, without distinct protuberances; disc of tergites III–V and sometimes VI, impunctate at middle; sternites III–IV and usually V with basal transverse lines sharply projected posteriad at middle; female tergite X transverse to weakly transverse, sharply produced apically, with beveled apex (Fig. 26E–F); female gonocoxite distinctly swollen at base (Fig. 27H).

##### Distribution.

Figure 29C. *Bolitogyrus fulgidus* is known from Nicaragua and Costa Rica (not east of the Central Cordillera).

##### Bionomics.

*Bolitogyrus fulgidus* has been collected in April, October and December/January at elevations ranging from 50–1550 m (but see below). All specimens with collecting data were captured in Malaise traps. Currently only females have been collected and nothing is known about this species’ microhabitat preferences. It is notable that this species was never collected by fogging fungusy logs or from other fungi. It is possible that this species normally occurs in a forest stratum higher than accessed by most collecting efforts.

##### Comments.

*Bolitogyrus fulgidus* is most similar to *Bolitogyrus pulchrus* and *Bolitogyrus salvini* but may be easily distinguished by the bicolored antennae and pale base of the elytra. Four recently collected female specimens *Bolitogyrus fulgidus* from Costa Rica are included in this species concept based on the shape of female tergite X, though in one specimen (SM0694452), the tergite is distinctly more elongate (Fig. 26F), antennomere X is not paler than the preceding segments, the pronotum-to-head width ratio is slightly greater, and the elytra are slightly longer. This specimen was also collected at a much higher elevation (1550 m) than the other specimens including the holotype (50-620 m) and from a locality on the Pacific slope of the continental divide in Costa Rica. *Bolitogyrus fulgidus* is otherwise known from the Caribbean lowlands of Nicaragua and Costa Rica. Additional collecting in Costa Rica will hopefully determine the identity of this specimen from the Pacific cloud forest, especially if males of this species are discovered from both side of the divide.

#### Bolitogyrus pulchrus

Taxon classificationAnimaliaColeopteraStaphylinidae

Brunke sp. n.

http://zoobank.org/70032F4F-F59B-4964-974A-2466C1A9D928

Figs 1C , 5E , 16A–B , 23C , 26D , 29C (map)

##### Type locality.

Costa Rica, San José, San Gerardo de Dota, Finca los Chacón, 9.5489, -83.8106 [co-ordinates from INBIO database].

##### Type material.

**Holotype** ♂ (INBIO): COSTA RICA, Prov. San José, Dota, Savegre, Finca los Chacón, 2151m, 30 JUN – 1 JUL 2002, R. González, ‘Libre’. [=hand collected], L_S_388700_484200 #70747 / INB0003522884, INBIOCRI COSTA RICA [INBIO barcode label] / Holotype, *Bolitogyrus pulchrus*, Des. A. Brunke [red label].

Material excluded from type series: **PANAMA:** Chiriqui Prov., La Fortuna, “Hydro Trail”, 8°42'N, 82°14'W, 1150 m, 23-V- 9 VI 1995, J. Ashe, R. Brooks, #156, ex: flight intercept trap [white printed label], SM0037993, KUNHM-ENT, 1 ♀ (SEMC).

##### Diagnosis.

Head without central protuberance; antenna and legs (except for coxa and base of antennomere II) dark brown (Fig. 5E); entire or apical half of abdominal segment VIII and entire genital segment dark, contrasting with pale segments III–V; abdominal sternites with fine transverse microsculpture, interspaces about as wide as lines (Fig. 13D); female procoxa completely pale, contrasting with rest of leg; arms of paramere divergent and with fields of peg setae extended basad (Fig. 16A–B); female tergite X with raised disc contiguous with base but converging strongly toward apex (Fig. 26D); female gonocoxite not distinctly swollen at base.

##### Description.

Measurements ♂ (n=1): HW/HL 1.43; PW/PL 1.37; EW/EL 1.37; ESut/PL 0.86; PW/HW 1.20; forebody length 5.1 mm.

Measurements ♀ (n=1): HW/HL 1.40; PW/PL 1.41; EW/EL 1.29; ESut/PL 0.79; PW/HW 1.14; forebody length 5.3 mm.

Similar to *Bolitogyrus buphthalmus* and differing only in the following: head and pronotum reddish orange with bronze metallic reflection (female specimen) or head dark with bluish-purple metallic reflection, pronotum with bright violet metallic reflection and margins broadly reddish (holotype); elytra with bluish-purple to violet metallic reflection and with extreme base reddish; abdominal segments III–V reddish, VI reddish (male holotype) or red with dark base (female specimen), VII reddish with dark apex (male holotype) or reddish (female specimen), VIII reddish with dark apex (female specimen) or entirely dark (male holotype), genital segment dark; all antennomeres dark brown, base of II dark red; legs not distinctly bicolored: coxa light reddish-brown and remainder of leg entirely dark brown; median frontal impression weak; head with punctation finer than in *Bolitogyrus buphthalmus*; last segment of labial palpus distinctly setose, setae more numerous and longer than in *Bolitogyrus buphthalmus*; pronotum with pair of depressions but without distinct protuberance in lateral view; male sternite VII with impressed, apical triangular area about as long as half the length of sternite; median lobe in lateral view recurved dorsad (c.f. Fig. 15F); paramere with divergent arms, distinctly more parallel sided in basal half, peg setae fields extended basad (Fig. 16A–B); male sternite IX similar to *Bolitogyrus buphthalmus* but with shallower and wider emargination and smaller base (Fig. 23C); female tergite X with raised disc contiguous with base but converging strongly toward apex, apex evenly formed to a point, not produced (Fig. 26D).

##### Distribution.

Figure 29C. Known from the Talamanca Cordillera in Costa Rica and in adjacent Panama (Chiriquí province).

##### Bionomics.

This species has been collected in May and June/July at 1150 and 2150 m from a flight intercept trap and by hand collecting. Nothing is known about this species’ habitat preferences.

##### Etymology.

The species epithet means ‘beautiful’ in Latin and refers to the striking combination of metallic purple and non-metallic red in this taxon.

##### Comments.

*Bolitogyrus pulchrus* is most similar in habitus to *Bolitogyrus fulgidus* but can be distinguished by the dark antennae and abdominal apex, the more uneven elytral disc and the differently shaped female tergite X. The female specimen is from a much lower elevation on the Caribbean slope of the Panamanian portion of the Talamancas and differs from the male holotype in abdominal and head coloration. Both specimens share a dark genital segment and the unique, entirely dark antennae and legs. However, the female specimen is excluded from the type series as it may represent another undescribed species closely related to *Bolitogyrus pulchrus*.

#### Bolitogyrus sallei

Taxon classificationAnimaliaColeopteraStaphylinidae

(Kraatz, 1858) stat. r.

Figs 11B , 16I , 23D , 25L , 29D (map)

Cyrtothorax sallei Kraatz, 1858: 367
Cyrtothorax sallei : Fauvel 1878: 164, as synonym of *buphthalmus* Erichson
Cyrtothorax sallei : Sharp 1884: 340, as synonym of *buphthalmus* Erichson

##### Type locality.

“Mexico”.

##### Type material.

*Cyrtothorax sallei* Kraatz, 1858 (ex. parte). LECTOTYPE (♂, here designated, DEI): Mexico [blue label, script]/ Coll. Kraatz [white label, printed]/ SYNTYPUS [red label, printed] / LECTOTYPE ♂, *Cyrtothorax sallei* Kraatz, 1858, des. A. Brunke 2013 [red label, printed].

The syntype series of *Cyrtothorax sallei* in DEI contains 5 specimens corresponding to three different species. Specimens #3-5 (2 ♂, 1 ♀) are conspecific with *Bolitogyrus buphthalmus* and specimens #1-2 (both ♂) are each a different species. Specimen #1 belongs to a species rather similar to *Bolitogyrus buphthalmus* and known only from Mexico. This specimen was designated as the lectotype of *Bolitogyrus sallei* to associate this species concept with an available name. Specimen #2 belongs to ‘morphotype 3’ of *Bolitogyrus salvini* (see comments under that species). The variation referred to by Kraatz (1858) as ‘var. nigricoxis’ corresponds to specimen #5, a female of *Bolitogyrus buphthalmus* with characteristically dark coxae.

##### Other material.

‘März’, 1 ♂ (NMW). **MEXICO:** “Cyrtothorax buphthalmus D.S.”, [two ♂ specimens mounted on one card], “Sharp Coll. 1905.-313”, Flohr, 2 ♂ (BMNH); Mexico, ‘6.J’ [on first specimen], J. Flohr, 2 ♂ (ZMHB). *Hidalgo*: Chapulhuacán, La Piedra, carretera a Picafiores, Selva Mediana Caducifolia [=mid-elevation deciduous forest], 841m, 21°9'46"N, 98°57'18.7"W, Tronco [=log], 8.IV.2006, J. Márquez y J. Asiain, 2 ♂ (UAEH); Chapulhuacán, Tamaulipas, bosque mesófilo de Montaña [=mountain cloud forest], 1115m, 21°9'56"N, 98°55'50.2"W, trampa interception de vuelo [=FIT], 14 to 17.VII.2007, P. Martinez, J. Asiain, I. Rodriguez & J. Márquez, 1 ♂, 1 ♀ (UAEH). *Veracruz*, ‘Cordova’ (=Córdoba), Höge, “Sharp Coll. 1905.-313”, 1 ♂ (BMNH).

##### Diagnosis.

Head without central protuberance; antenna and legs distinctly bicolored (Fig. 5A); genital and abdominal segment VIII not distinctly paler than previous segments; abdominal sternites with fine transverse microsculpture, interspaces about as wide as lines (Fig. 13D); female procoxa mostly pale (Fig. 11B); median lobe recurved dorsad at apex (Fig. 15F); paramere with divergent arms (Fig. 16I); paramere with fields of peg setae varying from wide and circular to narrow and angulate, at base of arms (c.f. Fig. 15C, D, G, H); female tergite X with raised disc not converging strongly toward apex, strongly pigmented area strongly incised at apicolateral margin (Fig. 25L); female secondary gonocoxite not distinctly swollen at base.

##### Redescription.

Measurements ♂ (n=5): HW/HL 1.58–1.80; PW/PL 1.27–1.71; EW/EL 1.24–1.43; ESut/PL 0.72–0.79; PW/HW 1.06–1.14; forebody length 3.8–4.4 mm.

Measurements ♀ (n=1): HW/HL 1.46; PW/PL 1.33; EW/EL 1.28; ESut/PL 0.75; PW/HW 1.05; forebody length 4.6 mm.

Similar to *Bolitogyrus buphthalmus* and differing only in the following: forebody distinctly shorter than in most specimens of *Bolitogyrus buphthalmus*; forebody (head and elytra) with bronze metallic reflections or not, never with green, purple or blue reflections; forecoxa in females yellow with some basal and lateral darkening; median lobe in lateral view recurved dorsad (c.f. Fig. 15F); paramere with fields of peg setae varying from wide and circular to narrow and angulate, at base of arms (c.f. Fig. 15C, D, G, H); male sternite IX with apical emargination slightly deeper and with asymmetrical base ending in a small knob (Fig. 23D); female tergite X with raised disc more elongate and only weakly converging toward apex (Fig. 25L).

##### Distribution.

Figure 29D. *Bolitogyrus sallei* is known only from the Mexican states of Hidalgo and Veracruz.

##### Bionomics.

*Bolitogyrus sallei* has been collected from cloud to deciduous forest, in June and July, at elevations ranging from 841-1115 m. One specimen was collected from a rotting log and another from a flight intercept trap.

##### Comments.

*Bolitogyrus sallei* is most similar to and sympatric with *Bolitogyrus buphthalmus* from which it differs in the smaller size of the forebody (overlapping but rarely), the partially pale forecoxa in females and the morphology of the aedeagus. It is rare for very similar and thus presumably closely related species of *Bolitogyrus* to occur sympatrically but future collecting will hopefully establish whether *Bolitogyrus sallei* and *Bolitogyrus buphthalmus* share the same habitat or microhabitat. Kraatz (1858) remarked that the black specimen in his type series of *Bolitogyrus sallei* could in fact represent a distinct species. Study of the male genitalia of this and additional, non-type specimens demonstrated this to be correct. By designation of this syntype as a lectotype, the name *Bolitogyrus sallei* was used for that species. Kraatz (1858) also observed differences in punctation of the abdominal tergites of specimens he examined but this variation did not correspond to the different species in his syntype series distinguished here.

#### Bolitogyrus salvini

Taxon classificationAnimaliaColeopteraStaphylinidae

(Sharp, 1884)

**Figure 3.**
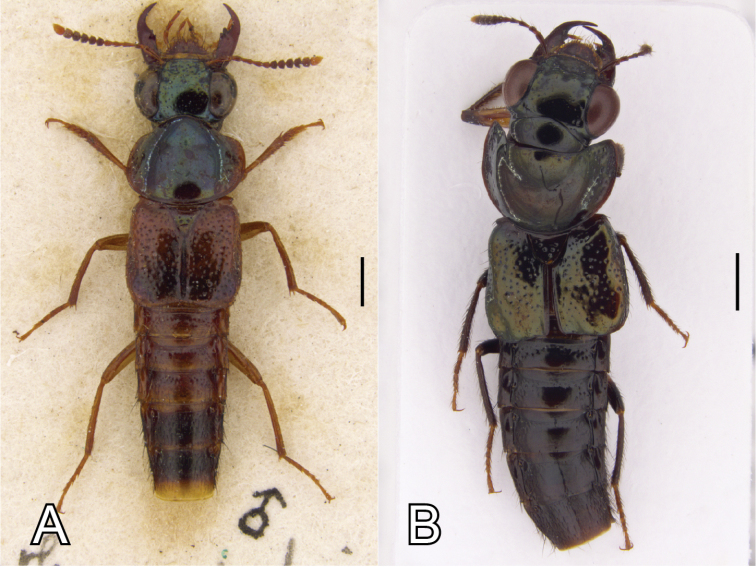
Habitus of *Bolitogyrus salvini* (Sharp): Morphotype I (male lectotype) (**A**); Morphotype III (non-type male) (**B**). Scale bars = 1 mm.

**Figure 4.**
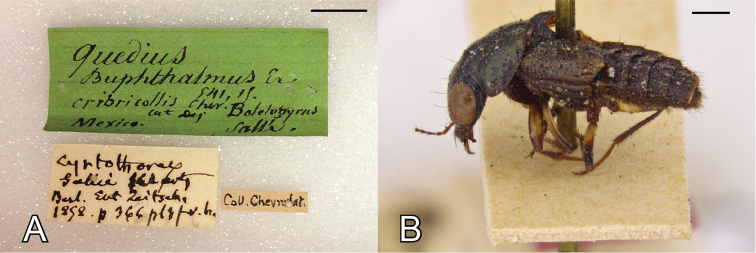
Syntype of *Quedius buphthalmus* Erichson (BMNH): historical labels (**A**); specimen (**B**). Scale bars = 10 mm (**A**), 1 mm (**B**).

Figs 3A–B , 10B , 16C–H , 23E–F , 26A–C , 27G , 30A (map)

Cyrtothorax salvini Sharp, 1884: 341
Cyrtothorax salvini : Scheerpeltz 1974: 183 (in key)

##### Type locality.

Zapote (=El Zapote, near Escuintla), Guatemala.

##### Type material.

*Cyrtothorax salvini* Sharp, 1884.

**Lectotype** (here designated, ♂ BMNH): “♂, Cyrtothorax salvini, ♂ Type D.S., Zapote, Guat.[=emala] Champion [written on card in Sharp’s hand with specimen]/ Zapote, Guatemala, Champion [printed label]/ B.C.A. Col. I.2., Cyrtothorax, salvini, Sharp. [printed label]/ Type [printed on disc with red border]/ Sharp Coll., 1905.-313. [printed label] / Lectotype ♂, *Cyrtothorax salvini* Sharp, 1884, des. A. Brunke 2013 [red printed label].

**Paralectotype** (1 ♀, BMNH): “♀, Cyrtothorax salvini, ♀ Type D.S., Zapote, Guat.[=emala] Champion [written on card in Sharp’s hand with specimen]/ Zapote, Guatemala, Champion [printed label]/ “G.” [handwritten]/ Type [printed on disc with red border]/ Sp. figured [printed]/ B.C.A. Col. I.2., Cyrtothorax, salvini, Sharp. [printed label] / Paralectotype *Cyrtothorax salvini* Sharp, 1884, det. A. Brunke 2013.

The male and female syntypes are identical in color and are easily separated from other species of the Buphthalmus Group based on male and female genitalia, though considerable variation exists within the present species concept. To provide nomenclatural stability for this name, the male syntype was selected and is here designated as the lectotype, as male sexual characters provide more reliable diagnostic characters than those of females in *Bolitogyrus*.

##### Other material.

**MEXICO:** Sallé, (ex. syntype series of *Bolitogyrus sallei*), 1 ♂ (DEI);

*Chiapas*: Parque Laguna Belgica, 19 km N. Ocozocoautla, 970m, 8.VI.1991, ex: flight intercept trap, J.S. Ashe Coll#75, SM0038002, 1 ♂ (SEMC), same except: 12.VI.1991, J.S. Ashe Coll#95, SM0038003; Playón de la Gloria, 16.14818°N, 90.89660°W, 180m, mature wet forest, malaise trap, 24.VI.2008, LLAMA08Ma-A-09-2-01, SM0833172, 1 ♀ (SEMC). **NICARAGUA:** *Jinotega*: Reserva Nationale Dantali El Diablo, 13.10367N, -85.86904W ± 10m, 1380m, cloud forest, ex. Malaise trap, 19 to 21.V.2011, LLAMA11Ma-D04-3-02, 1 ♀ (ZMUC, DNA extracted); Reserva Nationale Cerro Kilambé, 13.56980N, -85.69742 ± 10m, 1470m, cloud forest, ex. Malaise trap, 23 to 26.V.2011, LLAMA11Ma-D05-1-02, 1 ♀ (SEMC). *Matagalpa*: 6 km N Matagalpa, Selva Negra Hotel, 12°59.99'N, 85°54.53'W, 1350m, ex. sweeping vegetation, 21.V.2002, R. Brooks, Z. Falin, S. Chatzimanolis, SM0536468, 1 ♀ (SEMC).

##### Diagnosis.

**Figure 5.**
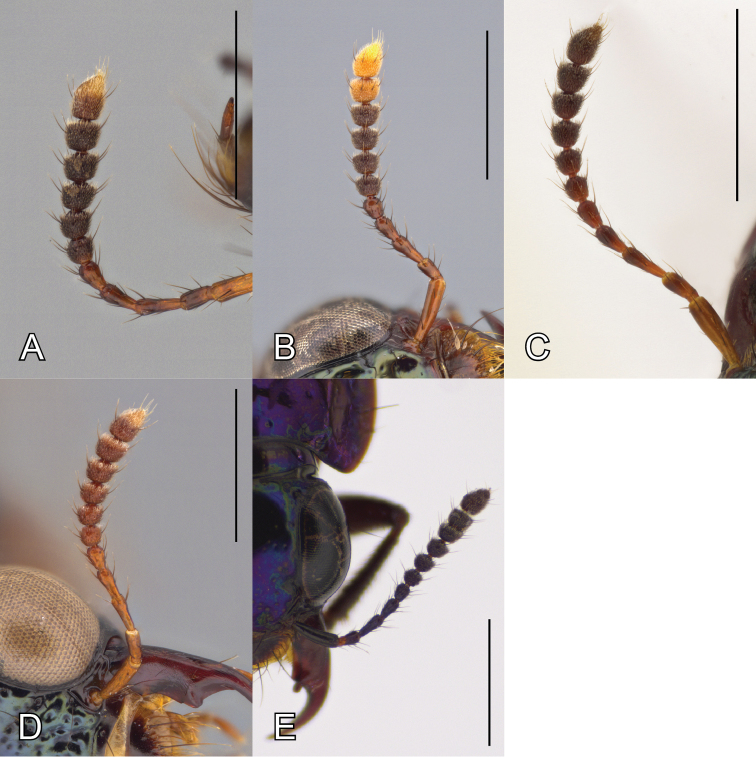
Antenna: rare specimen of *Bolitogyrus costaricensis* (Wendeler) with darkened apical segment (**A**); *Bolitogyrus fulgidus* (Sharp) (**B**); *Bolitogyrus marquezi* Brunke (**C**); *Bolitogyrus newtoni* Brunke (**D**); *Bolitogyrus pulchrus* Brunke (**E**). Scale bars = 1 mm.

Head without central protuberance; antenna distinctly bicolored (Fig. 5A); abdominal sternites with fine transverse microsculpture, interspaces about as wide as lines (Fig. 13D); median lobe in lateral view recurved dorsad (Fig. 15F); apical portion of median lobe in parameral view wide at constricted base (Fig. 16C); paramere with divergent arms (Fig. 16D–E); female tergite X narrow at base, raised area on disc contiguous with base and not strongly converging to apex (Fig. 26A–C); female gonocoxite not swollen at base (Fig. 27G).

##### Redescription.

Measurements ♂ (n=3): HW/HL 1.62–1.69; PW/PL 1.33–1.50; EW/EL 1.21–1.32; ESut/PL 0.71–0.74; PW/HW 1.09–1.14; forebody length 4.80–5.00 mm.

Measurements ♀ (n=4): HW/HL 1.43–1.50; PW/PL 1.35–1.50; EW/EL 1.25–1.32; ESut/PL 0.73–0.77; PW/HW 1.09–1.15; forebody length 5.0–5.1 mm.

Similar to *Bolitogyrus buphthalmus* and differing only in the following: head and pronotum metallic green to greenish bronze or black, without metallic reflection; elytra reddish or black with greenish to bronze metallic reflections, base of elytra concolorous with disc; abdominal segments III–V reddish or completely dark; abdominal segments VIII and genital segment yellow to yellow-orange, distinctly contrasting with previous segments, or entirely dark and not contrasting; legs distinctly bicolored (as in *Bolitogyrus buphthalmus*), with dark procoxa in females, or entirely reddish (the lectotype and paralectotype); median frontal impression weak to moderately impressed (morphotype 3); head impunctate in middle of disc or not (lectotype and paralectotype); disc of tergites III–V impunctate at middle, disc of VI punctate; median lobe in lateral view recurved dorsad; apical portion of median lobe constricted at base but weakly such that base is relatively wide; paramere with arms relatively flat, not curved around median lobe, arms divergent and peg setae as illustrated, varying from dense to sparse; male sternite IX similar to *Bolitogyrus buphthalmus* but median emargination deeper; female tergite X distinctive, narrow at base, raised area on disc contiguous with base and not strongly converging to apex; strongly pigmented area variable at apex.

##### Distribution.

Figure 30A. This species is known from southern Mexico, Guatemala and central Nicaragua (Matagalpa and Jinotega).

##### Bionomics.

All specimens were either swept from foliage or collected from Malaise/flight intercept traps during May and June. As in *Bolitogyrus fulgidus*, very few males are known and it is possible that this species normally occurs in a forest stratum higher than accessed by most collecting efforts. *Bolitogyrus salvini* has been collected from a wide range of forested habitats: from wet lowland forest (180 m) to cloud forest (1470 m).

##### Comments.

The relatively large amount of morphological variation observed in *Bolitogyrus salvini* indicates that a complex of species is likely to be involved but with the material currently available, a broad concept of *Bolitogyrus salvini* (wide median lobe in parameral view, elongate female tergite X) is preferred. There are three different morphotypes discernable from the material at hand that will need re-assessment in the future. The male and female types (Guatemala) represent morphotype 1: reddish elytra; pale apex of the abdomen; weak median frontal impression; without impunctate area on dorsal surface of the head. Morphotype 2 is represented by four females (southern Mexico, Nicaragua): dark elytra with green to greenish bronze metallic reflections; pale apex of abdomen; weak median frontal impression; with impunctate area on dorsal surface of head. Morphotype 3 is represented by two males and one female (Mexico, north of second morphotype): dark elytra with bronze metallic reflection; dark apex of abdomen; moderately deep median frontal impression; impunctate area on dorsal surface of head. The first and third morphotypes have males available and can be distinguished (at present) by the peg setae on the paramere: morphotype 1 with distinctly fewer peg setae (therefore sparser) than morphotype 3 (Fig. 16F versus 16G–H).

### Bullatus Lineage

**Figure 6.**
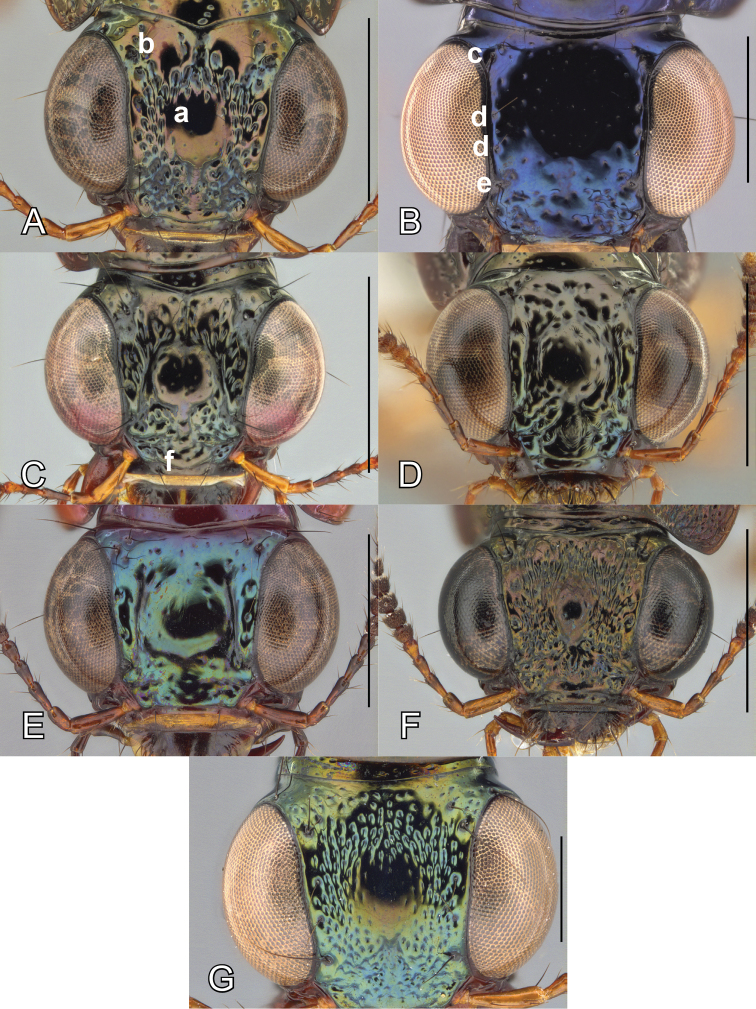
Head, dorsal view: *Bolitogyrus silex* Brunke (**A**); *Bolitogyrus costaricensis* (Wendeler) (**B**); *Bolitogyrus bullatus* (Sharp) (**C**); *Bolitogyrus apicofasciatus* Brunke (**D**); *Bolitogyrus ashei* Brunke (**E**); *Bolitogyrus strigifrons* (Wendeler) (**F**); *Bolitogyrus viridescens* Brunke (**G**). a – central protuberance, b – posterior protuberance, c – posterior ocular puncture, d – oculomarginal punctures, e – anterior ocular puncture, f – medial frontoclypeal puncture. Scale bars = 1 mm (**A–F**), 0.5 mm (**G**).

The species included in this lineage all share a raised, circular protuberance on the vertex, between the eyes (Fig. 6A), a feature not found in any other taxa in the tribe Staphylinini. This relatively large group of Neotropical species likely forms a monophyletic lineage of the genus and is a useful concept to contain other, smaller species groups. The Bullatus Lineage is distributed across the entire Neotropical range of *Bolitogyrus*, from Mexico to Ecuador.

#### Species *incertae sedis* within the Bullatus Lineage

##### Bolitogyrus bullatus

Taxon classificationAnimaliaColeopteraStaphylinidae

(Sharp, 1884)

Figs 1D , 6C , 10E , 11G , 14A , 18I–N , 23M , 26J , 30B (map)

Cyrtothorax bullatus Sharp, 1884: 340
Cyrtothorax bullatus : Scheerpeltz 1974: 182

###### Type locality.

PANAMA, Chiriquí, Boquete.

###### Type material.

**Holotype** ♂ (BMNH): *Cyrtothorax bullatus*, Type D.S., Boquete Panama, 3500 ft. Champion [hand written on card] / B.C.A. Col. I. 2., Cyrtothorax, bullatus, Sharp. [printed label] / Boquete, 3500 ft., Champion. [printed label] / Sharp Coll., 1905.-313. [printed label upside down] / Type [beige disc with orange border] / Holotype ♂, *Cyrtothorax bullatus* Sharp, 1884, det. A. Brunke 2013 [red printed label].

The single specimen located in the BMNH is interpreted as the holotype as Sharp (1884) stated that a single specimen was studied.

###### Other material.

**COSTA RICA:** *Alajuela*, Volcan Poas, 1700 m, cut over forest, trap 11, 20.VII to 3.VIII.1966, leg. S. Peck, 1 ♀ (FMNH). *Cartago*, 4 km NE Cañón, Genesis II, Cerro de la Muerte, 2350 m, ex. malaise trap, 1 to 30.V.1995, P. Hanson, CR1H93-95, SM0075668, 1 ♂ (SEMC), same except: 1 to 30.VIII.1995, CR1H93-95, SM0076840, 1 ♂ (SEMC); Genesis II Reserve, 9°42.57'N, 83°54.64'W, 2360 m, fungus covered logs, 13.VI.2004, J.S. Ashe, Z. Falin, I. Hinojosa, CR1AFH04 224, SM0607688, SM0607689, SM0607690, 3 ♂ (SEMC), same except: small gilled mushrooms on log, CR1AFH04 226, SM0607691, 1 ♀ (SEMC), same except: fleshy polypore CR1AFH04 230, SM0607693, 1 ♂ (SEMC); Genesis II Reserve, 9°42.57'N, 83°54.64'W, 2360 m, fungus covered logs, 14.VI.2004, J.S. Ashe, Z. Falin, I. Hinojosa, CR1AFH04 231, SM0607694, SM0607695, SM0607696, SM0607697, SM0607698, 4 ♂ 1 ♀ (SEMC); Genesis II Reserve, 9°42.57'N, 83°54.64'W, 2360 m, ex. orange gilled mushrooms on log, 14.VI.2004, J.S. Ashe, Z. Falin, I. Hinojosa, CR1AFH04 236, SM0607700, 1 ♀ (SEMC), same except: ex. small white gilled mushrooms, CR1AFH04 237, SM0607701, 1 ♂ (SEMC); Genesis II Reserve, 9°42.57'N, 83°54.64'W, 2360 m, ex. fungus covered logs, 15.VI.2004, J.S. Ashe, Z. Falin, I. Hinojosa, CR1AFH04 243, SM0607703, SM0607704, SM0607705, 3 ♂ (SEMC); La Congreja, 1950 m, IX–XII.1992, P. Hanson, SM0037998, 1 ♂ (SEMC; La Sierra, Finca Sinfonica, 1950 m, ex. fog rotten trunk, 20.III.2000, CR1083, 1 ♂ (PTC), same except: km 45 Pan Am. Hgwy, ex. fog rotten logs, CR-1084, 1 ♂ (PTC); La Sierra, Finca Sinfonica, 1970 m, ex. fog rotten logs, 23.X.2000, CR-1068, P.N. Thomas, 1 ♂ (PTC); Rio Macho Reserve, Macho Gaff, Cuenca Quebiri, Porton La Esperanza, 2460 m, ex. fog encino [=oak] logs, 24.III.2000, CR-1091, P.N. Thomas, 2 ♂ 2 ♀ (PTC); Tapanti National Park, 9°45'41"N, 83°47'5"W, 1050 m, 18.VII.2000, ex. fogging fungus covered log, J. Ashe, R. Brooks, Z. Falin, CR1ABF00 166, SM0203492, 1 ♂ (SEMC). *Heredia*, Baulio Corillo National Park, Vara Blanca, 2000 m, flight intercept trap, 13.II to 21.II.2002, ALAS-INBIO, 20/TN/12/006, 1 ♀ (PTC), same except: malaise, 10.III to 22.III.2002, M/07/047, 1 ♀ (PTC), same except: Berlese, 16.III.2002, 20/B/BM/056, 1 ♀ (PTC), same except: malaise, 22.III to 9.IV.2002, 20/M/01/061, 2 ♀ (PTC), same except: Malaise, 9.IV to 21.IV.2002, 20/M/13/093, 1 ♀ (PTC), same except: flight intercept, 10.IV to 21.IV.2002, 20/TN/17/030, 1 ♀ (PTC). *Puntarenas*, Altimira Biol. Sta., trail to Valle de Silenco, 9°01.76'N, 83°00.49'W, 1600-1700 m, ex. fungus covered logs, J.S. Ashe, Z. Falin, I. Hinojosa, CR1AFH04, SM0607671, 1 ♂ (SEMC); Área de Protección El Progresso, Sierra de Talamanca, selva alta subperenifolia [=high sub-evergreen forest], 8°55'296"N, 82°47'854"W, 1535 m, en troncos podridos [=in rotten logs], 25.II.2000, J.L. Navarrete col., 1 ♂ (MZFC); Hacienda La Amistad, 8°56.395'N, 82°47.465'W, 1500 m, premontane moist forest, fogging fungusy logs, 9.VI.2012, leg. Solodovnikov, Brunke Puliafico & Selvantharan, 3 ♂ 2 ♀ (ZMUC); Hacienda La Amistad, 8°58.102'N, 82°46.883'W, 1900 m, premontane to lower montane moist forest, fogging fungusy logs, 12.VI.2012, leg. Solodovnikov, Brunke Puliafico & Selvantharan, 3 ♂ 3 ♀ (ZMUC), same except: sifting rotting log litter, 1 ♂ (ZMUC), same as first except: sifting leaf litter near logs, 1 ♂ (ZMUC); Monte Verde, 1550-1570 m, ex. misc. mushrooms, 22.V.1989, J. Ashe, R. Brooks, R. Leschen, Costa Rica Exped. #370, SM0061182, 1 ♂ (SEMC); Monte Verde, Reserva Biologica de Monte Verde, nr. Quebrada Cuecha on Sendero Rio, 1580 m, ex. fungi, 13.V.1989, J. Ashe, R. Brooks, R. Leschen, Exped. #149, SM0061184, SM0061185, 2 ♂ (SEMC). *San Jose Prov.*, 2.4 km ENE San Gerado de Rivas, Cloudbridge Reserve, 9°28.47'N, 83°34.20'W, 1750 m, river trail, ex. fungus covered logs, 9.VI.2004, J.S. Ashe, Z. Falin, I. Hinojosa, CR1AFH04 158, SM0607551, SM0607552, SM0607553, SM0607554, 3 ♂ 1 ♀ (SEMC); 2.4 km ENE San Gerado de Rivas, Cloudbridge Reserve, 9°28.07'N, 83°33.84'W, ridge trail, 2000-2200 m, ex. fungus covered logs, 9.VI.2004, J.S. Ashe, Z. Falin, I. Hinojosa, CR1AFH04 162, SM0607549, SM0607550, 2 ♂ (SEMC), same except: 10.VI.2004, CR1AFH04 180, SM0607673, SM0607674, 1 ♂ 1 ♀ (SEMC); 2.4 km ENE San Gerado de Rivas, Cloudbridge Reserve, 9°28.68'N, 83°34.00'W, ridge above covered bridge, 1860 m, ex. fungus covered logs, 11.VI.2004, J.S. Ashe, Z. Falin, I. Hinojosa, CR1AFH04 200, SM0607678, 1 ♂ (SEMC); same except: 9°28.07'N, 83°33.84'W, flight intercept trap, 9 to 12.VI.2004, CR1AFH04 200, SM0607410, 1 ♂ (SEMC); 2.4 km ENE San Gerado de Rivas, Cloudbridge Reserve, 9°28.47'N, 83°34.20'W, river trail, 1750 m, flight intercept trap, 9.VI to 12.VI.2004, J.S. Ashe, Z. Falin, I. Hinojosa, CR1AFH04 202, SM0607435, 1 ♂ (SEMC); Division, Avalon Lodge, 2100 m, ex. ‘mushrooms log’, 29.IV.1998, CR-906, P.N. Thomas, 1 ♂ (PTC); Division, Sto. Eduviges, 0.4 ml SW of Avalon, ex. dry cliff humus, Berlese, 8.III.2004, CR-1576, P.N. Thomas, 1 ♂ (PTC); El Rosario Reserve, Cerro Caraigres, 2000 m, ex. tree moss, Berlese, CR-1492, 20.XI.2003, P.N. Thomas, 1 ♂ (PTC); Jardin, Quebradillas, Quinta Colibri, 2040 m, ex. ‘murta treefall’, Berlese, 21-XII-2007, CR-1587A, P.N. Thomas, 1 ♀ (PTC), same except: ex. leaf litter debris, Berlese, 22.III to 2.IV.2009, CR-1618, 1 ♀ (PTC); Jardin, Quebradillas, Quinta Colibri, 2040 m, ex. tree mosses, Berlese, 5.XII.2008, CR-1605, P.N. Thomas, 1 ♀ (PTC), same except: ex. fog oak log, 4.V.2008, CR-1602E, P.N. Thomas, 1 ♂ (PTC); Jardin de Empalme Dota, 2160 m, ex. fog encino [=oak] log, 17 to 18-III.2000, P. N. Thomas, 3 ♂ 2 ♀ (PTC); Jardin de Empalme, Finca H. Urena, 2070 m, 20.I.2002, ex. fog encino log, CR-1283, P.N. Thomas, 1 ♂ (PTC), same except: ex. flight intercept, 27.I to 3.II.2002, 1 ♀ (PTC); La Potentiana de Cerro Turrubares, Finca Mayo Corales, 1380 m, fog rotten logs, CR-1115, P.N. Thomas, 2 ♀ (PTC); San Francisco de Leon Cortez, 1780 m, ex. fog dry logs, 3.IV.2000, CR-1119, P.N. Thomas, 8 ♂ 2 ♀ (PTC); same except: 1860 m, ex. fog huge log in large ravine, 4.IV.2000, CR-1121 7 ♂ 2 ♀ (PTC); Jardin de Empalme, 2160 m, ex. dried mushroom and subbark on log, 5.V.1998, CR-936, P.N. Thomas, 1 ♀ (PTC); PanAmerican Hwy, 7 km SSW km 80.5, Cabinas de Quetzal, 9°33'53"N, 83°48'5"W, 2150 m, fogging fungus covered log, 22.VII.2000, J. Ashe, R. Brooks, Z. Falin, CR1ABF00 223, SM0200461, SM0200462, 1 ♂ 1 ♀ (SEMC); PanAmerican Hwy, 9.5 km SSW km 80.5, on San Gerardo Rd., catarata trail, 9°32'47"N, 83°48'40"W, 2020 m, fogging fungus covered log, 23.VII.2000, J. Ashe, R. Brooks, Z. Falin, CR1ABF00 229, SM0152909, 1 ♂ (SEMC); San Gerardo de Dota, La Catarata, 9°32.80'N, 83°48.68'W, 2225 m, ex. fungus covered logs, 12.VI.2004, J.S. Ashe, Z. Falin, I. Hinojosa, CR1AFH04 203, SM0607687, 1 ♀ (SEMC), same except: 26.V.2004, CR1AFH04 001, SM0607651, 1 ♀ (SEMC); San Juan, 14 km NE, Finca Zurqui, 10°2'57"N, 84°0'22"W, 1490 m, ex. fogging fungus covered log, 6.VII.2000, J. Ashe, R. Brooks, Z. Falin, CR1ABF00 065, SM0203233, SM0203234, SM0203235, 3 ♂ (SEMC); Zurquí de Moravia, 1600 m, ex. malaise trap, 1 to 30.III.1995, CR1H93-95 12, P. Hanson, SM0075493, 1 ♂ (SEMC), same except: 1 to 30.V.1995, CR1H93-95 13, SM0075387, 1 ♀ (SEMC). **PANAMA:** *Chiriquí*, 2.9 km S Cerro Pando, 8°53'18"N, 82°44'48"W, ex. on *Clistocybe*?, 18.VI.1996, J. Ashe, R. Brooks, PAN1AB96 188A, SM0042775, 1 ♀ (SEMC); 3.5 km E Escopeta, Cerro Bollo, 8°34'N, 81°50'W, 1856 m, cloud forest, Berlese moss, bark and subcortex, leg. J. Wagner, FM (HD)#80-48, 2 ♂ (FMNH); 5.4 km NE Boquete, 8°48'N, 82°26'W, 1520 m, ex. fogging fungusy log, 19.VI.1995, J. Ashe & R. Brooks, #247, SM0002098, SM0002120, SM0002471, SM0002490, SM0003167, 4 ♂ 1 ♀ (SEMC); 6.0 km NE Boquete, 8°48'0"N, 82°26'0"W, 1600 m, ex. fungusy log, 14.VI.1996, J. Ashe, R. Brooks, PAN1AB96 151, SM0042173, 1 ♂ (SEMC), same except: 1500 m, PAN1AB96 144, SM0050517, 1 ♂ (SEMC), same except: 1650 m, flight intercept trap, 14 to 19.VI.1996, PAN1AB96 180B, SM0044303, 1 ♂ (SEMC); same except: 1550 m, flight intercept trap, 14 to 19.VI.1996, PAN1AB96 179B, SM0042438, 1 ♂ (SEMC); 20 km N Gualaca, Finca La Suiza, 8°39'N, 82°12'W, 1350 m, ex. *Favolus hexagonalis*, 21.V.1995, J. & A. Ashe, #035, SM0006014, 1 ♂ (SEMC); same except: 22.V.1995, ex. fungusy log, #036, SM0004705, SM0004711, SM0007140, SM0005371, 2 ♂ 2 ♀ (SEMC); 20 km N Gualaca, Finca La Suiza, 8°39'N, 82°12'W, 1350 m, ex. flight intercept, 24.V to 9.VI.1995, J. Ashe & R. Brooks, #154, SM0007010, SM0007013, SM0007021, 3 ♂ (SEMC); same except 10 to 13.VI.1995, #196, SM0007028, 1 ♂ (SEMC); same except 10.VI.1995, fogging fungusy log, #167, SM0010456, SM0001900, SM0007030, 2 ♂ 1 ♀ (SEMC); same except: 1200 m, fogging fungusy log, 12.VI.1995, #190, SM0005622, SM0008015, 2 ♂ (SEMC); same except: 1450 m, fogging fungusy log, 12.VI.1995, #192, SM0000742, SM0000726, 1 ♂ 1 ♀ (SEMC); 24 km S Gualaca, Finca La Suiza, 8°34'0"N, 82°12'0"W, 1285 m, 31.V.2000, H. & A. Howden, CR1EH99 08, SM0235823, SM0235825, SM0235826, 2 ♂ 1 ♀ (SEMC); 27.7 km W Volcan, Hartmann’s Finca, 8°51'42"N, 82°44'48"W, 1650-1700 m, ex. fungusy log, 17.VI.1996, J. Ashe, R. Brooks, PAN1AB96 164, SM0049609, SM0049625, 2 ♀ (SEMC); 27.7 km W Volcan, Hartmann’s Finca, 8°51'48"N, 82°44'36"W, 1450 m, ex. fungusy log, 17.VI.1996, J. Ashe, R. Brooks, PAN1AB96 168, SM0050713, SM0050718, SM0050719, 2 ♂ 1 ♀ (SEMC); 27.7 km W Volcan, Hartmann’s Finca, 8°45'N, 82°48'W, 1450 m, ex. fogging fungusy log, 15.VI.1995, J. Ashe, R. Brooks, #223, SM0008003, SM0021124, 2 ♂ (SEMC), same except: 16.VI.1995, #226, SM0004880, SM0005684, 1 ♂ 1 ♀ (SEMC); Cerro Pando, 8°54'42"N, 82°43'18"W, 1875 m, ex. flight intercept trap, 17 to 18-VI-1996, J. Ashe, R. Brooks, PAN1AB96 185A, SM0016390, SM0038871, 2 ♂ (SEMC), same except 1850 m, PAN1AB96 184A, SM0016541, 1 ♂ (SEMC), same except: 1890 m, ex. fungusy log, 18.VI.1996, PAN1AB96 187A, SM0043548, 1 ♂ (SEMC); Hornito, Finca La Suiza, 8°39'N, 82°12'W, 1220 m, flight intercept trap, 31.V.2000, H. & A. Howden, SM0454113, 1 ♂ (SEMC), same except: 6.VI.2000, FMHD#2000-171, 1 ♀ (FMNH).

###### Diagnosis.

Within Bullatus Lineage: one setose puncture in dorsal row of pronotum; elytra dark, without bright metallic reflection; scutellum dark, not reddish; elytra without strigose patch; femur with apical dark band separated from the apex by a distance greater than half the length of the band (Fig. 11G).

###### Redescription.

Measurements ♂ (n=5): HW/HL 1.45–1.59; PW/PL 1.33–1.54; EW/EL 1.17–1.23; ESut/PL 0.81–0.96; PW/HW 1.03–1.09; forebody length 3.9–4.2 mm.

Measurements ♀ (n=5): HW/HL 1.46–1.57; PW/PL 1.28–1.42; EW/EL 1.11–1.23; ESut/PL 0.87–0.93; PW/HW 0.97–1.09; forebody length 4.4–5.3 mm.

Coloration: Body medium to dark brown, head and pronotum with weak bronze metallic reflection, frons occasionally with faint bluish-bronze metallic reflection, pronotum paler brown laterally, elytra dark brown, often with paler apex, entirely reddish-brown in some possibly teneral specimens, epipleuron reddish brown; abdominal tergites medium to dark brown on disc, their apex often paler, reddish-brown; paratergites variably paler, reddish brown; apex of tergite VII with triangular or semi-circular pale spot of variable size and width (one specimen seen lacking this spot). Ventral abdomen with paler areas variably developed: base of sternites III–VI with pair of lateral diffuse reddish-brown spots, or with lateral stripe along sternites III–VI, lighter areas sometimes extending from lateral stripe to apical margin; some specimens from the eastern Talamancas with sternites III to the base of sternite VII entirely reddish, apex of VII and entire VIII dark brown. Maxillary and labial palpi pale to dark brownish; antennomeres I–X grading from reddish-yellow (I) to brown or black (X), apical segment usually distinctly paler, varying from very light yellow to medium brown. Legs bicolored: forecoxa yellow, meso- and metacoxa dark yellow brown, femur yellow with dark brown band of variable length, situated apicad of middle (band on profemur sometimes incomplete), band on hind femur separated from apex by a distance greater than half its length; tibia brown to dark brown with medial face lighter, tarsus light brown.

Head with median frontal impression present as pair of parallel lines forming anterior margin of central protuberance; frons with coarse, irregular sculpture; with central protuberance, protuberance smooth, glossy, usually with a few asetose punctures and sometimes with fine median sulcus; base of head with posterior protuberances clearly separated medially with a channel, surface with asetose, deeply impressed punctures, impressions often forming deep sulci.

Pronotal disc with few to many asetose, fine punctures varying from barely to deeply impressed, lateral areas explanate, with well-impressed asetose punctures, spaced irregularly, often touching; protuberance distinct in lateral view; with one puncture in dorsal row (i.e. only marginal puncture); scutellum with several shallow to moderately impressed, confluent punctures; elytra weakly transverse, suture slightly shorter than pronotum at midline; surface without microsculpture, strongly uneven with protuberances; setae in rows relatively long and erect, distinct from overall surface sculpture in lateral view.

Abdominal tergites with sparse, long and gold pubescence, III–VI impressed at base; disc of tergites III–VI impunctate medially, this area becoming smaller on successive tergites; sternites III–V with basal transverse line sharply projected posteriad at middle, abdominal sternites with moderately coarse microsculpture, interspaces slightly larger than lines.

Median lobe in lateral view curved ventrad from midlength, outline variable in shape (many intermediates) as in Fig. 18K–L but always with indentation at midlength; in parameral view, narrowed to acute apex at apical third; paramere in parameral view not or only slightly divided apically but always with median suture, shape varying from nearly parallel to slightly expanded near midlength (Fig. 18I–J); paramere shorter to longer than median lobe (with intermediates); peg setae arranged in two lateral, single or variably doubled rows at apex of paramere (Fig. 18M–N). Male sternite VIII with transverse basal line broken at middle, with weakly emarginate apex, impressed and glabrous in small triangular area near emargination; male sternite IX sparsely setose, distinctly asymmetrical at base and with deep emargination at apex (Fig. 23M).

Female tergite shield-shaped, with acute, non-emarginate apex (Fig. 26J); female laterotergal sclerites slightly expanded at base and overlapping with tergite X (Fig. 26J).

###### Distribution.

Figure 30B. *Bolitogyrus bullatus* occurs along the Tilarán, Central and Talamanca Cordilleras (Costa Rica and western Panama).

###### Bionomics.

*Bolitogyrus bullatus* has been collected using a variety of passive collecting methods (Malaise, FIT), fogging fungusy logs and by sifting leaf litter and decaying wood debris, which were sometimes processed in Berlese funnels. Specimens were also hand collected directly from fungi including ‘small white gilled’ and ‘orange gilled’ mushrooms, ‘*Clistocybe*?’ and ‘*Favolus hexagonus*’. This species has been collected in mid-elevation forests at elevations ranging from 1050-2460 m, in all months of the year.

###### Comments.

Among species of similar distribution, *Bolitogyrus bullatus* is most similar in habitus to *Bolitogyrus falini* and the dark species from the Ashei Group. It can be easily distinguished by the single puncture in the dorsal row of the pronotum. Within its distribution, *Bolitogyrus bullatus* is the most commonly collected species of the Bullatus Lineage. Given the wide elevational occurrence of *Bolitogyrus bullatus*, the populations of the Tilarán, Central and Talamanca Cordilleras appear to be, at most, weakly isolated from each other. However, the specimens from west of the Central Cordillera are all females and males are needed to fully confirm the occurrence of *Bolitogyrus bullatus* there. This species shows a wide range of external morphological variation, none of which corresponds with the observed variation of the paramere and median lobe. Only color appears to be geographically correlated: an extreme color morph exists in some Panamanian populations where the abdomen is solid red ventrally except for the apex of sternite VII and entire VIII. However, intermediates between the typical red striped and solid individuals were observed from Panama and also Costa Rica.

##### Bolitogyrus apicofasciatus

Taxon classificationAnimaliaColeopteraStaphylinidae

Brunke sp. n.

http://zoobank.org/CD87F64C-3FF2-4E43-AA32-9F1197BC15C6

Figs 6D , 11H , 14B , 18F–H , 23O , 26L , 30B (map)

###### Type locality.

MEXICO, Chiapas, 2 km SE Custepec.

###### Type material.

**Holotype** ♂ (SEMC): MEXICO, Chiapas, 2 km SE Custepec, 15.72219°N, 92.94640°W, 1610 m, 19-V-2008, beating vegetation, cloud forest, LLAMA08 Go-A-02-3-03 [white printed label] / SM0822854 [white barcode label] / SUBJECT TO RETENTION AGREEMENT w/ ECOSUR, see KUNHM acc. # 2010-EN-089, to be removed with fulfilled [green printed label] / Holotype, *Bolitogyrus apicofasciatus* Brunke, sp. n. [red printed label].

**Paratypes** (3♀ SEMC): same as holotype except: 15.72236°N, 92.94585°W, 1620 m, mesophil forest, LLAMA08 Go-A-02-3-02, SM0839743 (SEMC). **GUATEMALA:** *Quetzaltenango*, 12.5 km SW Zunil, 1520 m, 21 June 1993, R. Anderson, #93-11, second-growth, SM0037995, 1 ♀ (SEMC) [missing head and pronotum], same except: 20 June 1993, J. Ashe, R. Brooks, #067, ex. *Auricularia* sp., SM0037984, 1 ♀ (SEMC).

###### Diagnosis.

Within Bullatus Lineage: one setose puncture in dorsal row of pronotum; elytra dark, without bright metallic reflection; scutellum dark, not reddish; elytra without strigose patch; femur with dark band separated from the apex by a distance distinctly shorter than half the length of the band (Fig. 11H).

###### Description.

Measurements ♂ (n=1): HW/HL 1.45; PW/PL 1.50; EW/EL 1.14; ESut/PL 1.00; PW/HW 1.03; forebody length 3.5 mm.

Measurements ♀ (n=2): HW/HL 1.41–1.43; PW/PL 1.36–1.45; EW/EL 1.18–1.19; ESut/PL 0.92–1.00; PW/HW 1.07–1.10; forebody length 4.2–4.5 mm.

Similar to *Bolitogyrus bullatus* and differing only in the following: pronotum dark brown throughout, abdominal segments (except VIII) broadly paler at apex, without paler lateral stripes, antennomeres I–X grading from reddish-yellow (I) to dark brown, apical segment not or barely paler than previous, brown to dark brown; procoxa light yellow, meso- and metacoxa yellowish-brown (male) or darker, brown (female); profemur without (male) or with short, nearly apical dark band, meso and metafemur with dark band close to apex, band on metafemur separated from the apex by a distance distinctly shorter than half the length of the band; median frontal impression present as in *Bolitogyrus bullatus* but sometimes obscured by irregular sculpture; central protuberance without fine median sulcus, base of head without distinct posterior protuberances, punctures of head impressed but these impressions not forming sulci, disc of pronotum markedly glossy, with very few punctures; protuberance of pronotum only weakly visible in lateral view; macrosetae of elytra relatively short, not distinct from overall surface sculpture in lateral view; abdominal sternites with relatively coarse microsculpture, interspaces wider than lines; median lobe in lateral view gently curved ventrad from base, apical portion more slender and more strongly curved ventrad near apex (Fig. 18G), similar to *Bolitogyrus bullatus* in parameral view (Fig. 18F), paramere in parameral view not divided apically but with median suture, distinctly extended to midlength (Fig. 18F), paramere slightly shorter than median lobe; paramere with peg setae arranged in a pair of lateral rows, which are wider (2–3 peg setae wide) near apex and base of row (Fig. 18H); male sternite VIII with transverse basal line complete; male sternite IX more densely setose, asymmetrical at base and with much shallower emargination at apex than *Bolitogyrus bullatus* (Fig. 23O); female tergite X strongly constricted at base, with broadly rounded apex (Fig. 26L); basal margin of laterotergal sclerites fused and thickened across base of tergite X (Fig. 26L); female laterotergal sclerites strongly expanded at base and overlapping with tergite X (Fig. 26L).

###### Distribution.

Figure 30B. Known from Chiapas, Mexico and Quetzaltenango, Guatemala.

###### Bionomics.

Specimens were collected in cloud and mesophil forests at 1520–1620 m, by beating vegetation and from an *Auricularia* (jelly) fungus, during May and June.

###### Etymology.

The specific epithet means ‘apical band’ and refers to the dark color band of the hind femur, which is situated distinctly closer to the apex than in *Bolitogyrus bullatus*.

###### Comments.

Within its distribution, *Bolitogyrus apicofasciatus* cannot be confused with any described species of *Bolitogyrus*.

#### Ashei Group

The species of the Ashei Group (*Bolitogyrus ashei*, *Bolitogyrus pseudotortifolius*, *Bolitogyrus tortifolius*) share an asymmetrical, somewhat leaf-shaped paramere (e.g., Fig. 19A, D) and a relatively short and truncate female tergite X that is unmodified at the base and is distinctly shorter than the laterotergal sclerites (about half the length) (Fig. 26K); both of these characters are unique to this group in the Neotropical *Bolitogyrus*. The Ashei Group is endemic to Costa Rica as far as known.

##### Bolitogyrus ashei

Taxon classificationAnimaliaColeopteraStaphylinidae

Brunke sp. n.

http://zoobank.org/ADACDCC2-FD1B-4D62-9526-C99E5AB03574

Figs 1E , 6E , 12B , 19A–C , 23N , 26K , 29C (map)

###### Type locality.

COSTA RICA: Cartago, Tapantí National Park

###### Type material.

**Holotype** ♂ (SEMC): Costa Rica, Cartago, P.N. Tapanti, 1150 m, 9°45'41"N, 83°47'5"W [sic!] [W], 18 JUL 2000, J. Ashe, R. Brooks, Z. Falin, CR1ABF00 165, ex: fogging fungus covered log [white printed label] / SM0203484 [white barcode label] / Holotype, *Bolitogyrus ashei* Brunke, sp. n. [red printed label].

**Material excluded from type series.** **COSTA RICA:** *Heredia*, 16 km SSE La Virgen, 1050–1150 m, ex. malaise, 21.II to 9.III.2001, ALAS-INBIO, 11/M/13/033, 1 ♀ (PTC).

###### Diagnosis.

Within the Bullatus Lineage: easily distinguished by the brightly colored, metallic purple to green elytra and completely orange-red abdomen (Fig. 1E).

###### Description.

Measurements ♂ (n=1): HW/HL 1.64; PW/PL 1.45; EW/EL 1.26; ESut/PL 0.83; PW/HW 1.17; forebody length 4.3 mm.

Measurements ♀ (n=1): HW/HL 1.55; PW/PL 1.48; EW/EL 1.16; ESut/PL 1.00; PW/HW 1.03; forebody length 4.8 mm.

Coloration: Head dark dorsally with metallic green reflection, with orange-red base, ventral surface dark reddish, or head completely orange-red; pronotum completely orange-red, scutellum orange-red contrasting with darker elytra that has a metallic bluish-green or purple reflection, ventral surface of pterothorax reddish, abdomen completely orange-red, maxillary and labial palpi pale reddish-brown, segment II of maxillary palpi darkened apically or not, segment III darkened, antennomeres I–VI pale reddish-brown, III–V darkened apically, VII–XI dark brown; procoxa entirely pale, orange and slightly darker than profemur (male) or yellow, concolorous with profemur (female), all femora darkened dorsally, pro- and mesofemur with dark apical band, metafemur with long apical dark band, completely reaching apex (male) or nearly so (female), tibia completely dark brown, contrasting with light yellow-brown tarsus.

Head with median frontal impression present as shallow, ‘X’ shaped impression contiguous with anterior margin of central protuberance; frons relatively smooth, glossy with several coarse, asetose punctures; disc with relatively weak central protuberance, poorly defined, especially posteriad, central protuberance smooth, glossy with several, sparsely distributed, fine asetose punctures; base of head with weakly pronounced posterior protuberances, surface with scattered, fine asetose punctures; microsculpture absent dorsally except as well-developed, fine lines on temples.

Pronotum distinctly transverse; disc impunctate, lateral areas explanate (female) to deeply explanate (male), with shallow, asetose punctures; protuberance distinct in lateral view, less pronounced in female, with one puncture in dorsal row (i.e. only marginal puncture); scutellum with several poorly to well-impressed, usually contiguous punctures; elytra weakly transverse, suture slightly shorter to equal in length to pronotum at midline; surface without microsculpture, uneven, with protuberances; setose punctures with setae relatively long and erect, distinct from overall surface sculpture in lateral view.

Abdominal tergites with sparse, long and black setae, III–VI impressed at base; disc of tergites III–VI impunctate medially, this area becoming smaller on successive tergites; sternites III–V with basal transverse line sharply projected posteriad at middle; abdominal sternites with fine, poorly defined microsculpture, interspaces about as wide as lines or narrower.

Median lobe in lateral view curved slightly ventrad, constricted in apical third, apical portion subparallel to broadly rounded apex (Fig. 19B); median lobe in parameral view subparallel, with strongly asymmetrical apical portion, minutely notched at broad, truncate apex (Fig. 19A); paramere in parameral view slightly divided apically and with median suture, strongly constricted at midlength and dilated in strongly asymmetrical, apical portion (Fig. 19A); paramere slightly longer than median lobe; peg setae arranged in a pair of lateral, elongate, but dissimilar groups, generally becoming wider at base and at apex (Fig. 19C). Male sternite VII shallowly but distinctly emarginate, with glabrous area apicomedially, this area not flattened; male sternite VIII with transverse basal line complete, with shallowly emarginate apex, impressed and glabrous in small triangular area near emargination; male sternite IX distinctly asymmetrical at base, with relatively shallow but distinct emargination at apex (Fig. 23N).

Female tergite broad with truncate, barely concave apex, short and about half as long as laterotergal sclerites (Fig. 26K); female laterotergal sclerites slightly expanded at base and overlapping with tergite X (Fig. 26K).

###### Distribution.

Figure 29C. Known from Cartago and Heredia state in Costa Rica.

###### Bionomics.

The male holotype was fogged from a fungus-covered log in July, at 1150 m and the female specimen was collected in a Malaise trap during February-March at a similar elevation.

###### Etymology.

It is our pleasure to dedicate one of the most spectacular species of *Bolitogyrus* to the late Dr. James Stephen (“Steve”) Ashe (1947–2005). The *Bolitogyrus* material from the University of Kansas (SEMC) insect collection has proven critical to the present revision, and Steve was the driving force behind and co-collector of the majority of these specimens. His passion for Staphylinidae, including *Bolitogyrus* is remembered in Lingafelter et al. (2006).

###### Comments.

*Bolitogyrus ashei* cannot be confused with any other member of the Bullatus Lineage. The female specimen was not included in the type series as it differs in coloration (completely orange head, purple elytra) and was collected on the Caribbean side of the continental divide in Costa Rica. As both specimens were collected at relatively low elevations (1050-1150 m), they may each represent allopatric species on either side of the continental divide. Additional specimens, especially males, are needed to re-evaluate this hypothesis.

##### Bolitogyrus tortifolius

Taxon classificationAnimaliaColeopteraStaphylinidae

Brunke sp. n.

http://zoobank.org/F4C2373C-AA8D-47B4-83B4-CB99E9BC544B

**Figure 7.**
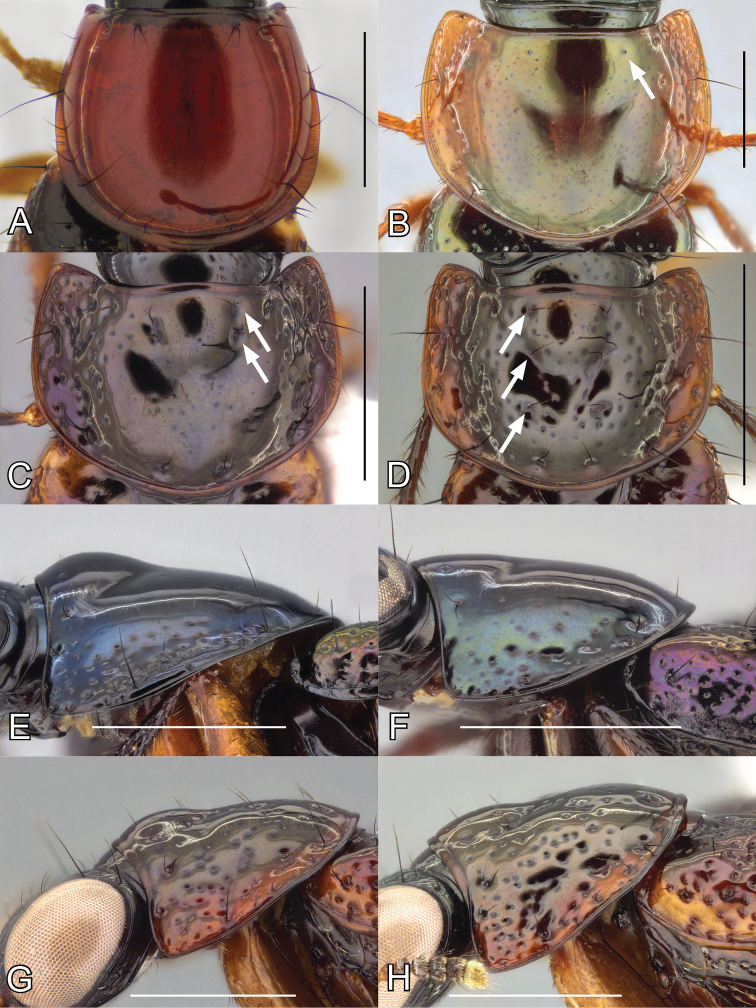
Pronotum, dorsal: *Bolitogyrus pictus* Smetana & Zheng (**A**); *Bolitogyrus fulgidus* (Sharp) (**B**); *Bolitogyrus tortifolius* Brunke (**C**); *Bolitogyrus divisus* Brunke (**D**). Pronotum, lateral: *Bolitogyrus costaricensis* (Wendeler), male (**E**), female (**F**); *Bolitogyrus divisus*, male (**G**), female (**H**). Scale bars = 1 mm.

Figs 1F , 7C , 10C , 19D–F , 24A , 27A , 30C (map)

###### Type locality.

Costa Rica, Puntarenas, Monteverde.

###### Type material.

**Holotype** ♂ (SEMC): COSTA RICA: Puntarenas, Monteverde, 29-31 June 1992, M.L. Jameson, ex. flight intercept trap [white printed label] / SM0038007 [white barcode label] / Holotype, *Bolitogyrus tortifolius* Brunke, sp. n. [red printed label].

**Paratypes** (5 ♂ 1 ♀, SEMC, ZMUC): COSTA RICA: *Puntarenas*, Monteverde, 1540 m, ‘ex. *Crepidotus* (white)’, 6.V.1989, J. Ashe, R. Leschen, R. Brooks, #107, SM0038004, 1 ♂ (SEMC); Monteverde, Cerro Amigos, 1520-1550 m, 6.V.1989, exp. #014, J. Ashe, R. Brooks, R. Leschen, SM0038010, 1 ♂ (SEMC); Monte Verde, 1520 m, exped. flight intercept trap, 21.V.1989, J. Ashe, R. Brooks, R. Leschen, exped. #316, SM0038009, 1 ♂ (SEMC); Monteverde, 1610 m, ex. flight intercept trap, 29.VI.1990, S.E. Roberts, SM0038012, 1 ♂ (SEMC); Monteverde, 1520 m, ex. flight intercept trap, 11.V.1989, J. Ashe, R. Brooks, R. Leschen, exped. #111, SM0038011, 1 ♀ (SEMC); Monteverde Biological Station, 10°19.672'N, 84°49.141'W, 1515 m, cloud forest, ex. flight intercept trap, 10 to 17.VI.2001, S. & J. Peck, 01-10, CR1P01002, SM0551690, 1 ♂ (ZMUC).

###### Diagnosis.

Within the Bullatus Lineage: two punctures in dorsal row of pronotum (Fig. 7C); dark abdominal segment VIII; humerus with pale marking distinctly narrower than half the distance between scutellum and humeral angle (Fig. 10C); median lobe in lateral view slightly recurved dorsad, usually with a sharp angle at the place of recurvature (Fig. 19E).

###### Description.

Measurements ♂ (n=5): HW/HL 1.70–1.83; PW/PL 1.56–1.67; EW/EL 1.19–1.27; ESut/PL 0.91–1.00; PW/HW 1.03–1.06; forebody length 3.6–3.8 mm.

Measurements ♀ (n=1): HW/HL 1.68; PW/PL 1.61; EW/EL 1.29; ESut/PL 1.00; PW/HW 1.00; forebody length 3.9 mm.

Coloration: Body dark brown, frons with faint green-blue metallic reflection, pronotum with faint bronze metallic reflection; pronotum with lateral portions broadly paler, reddish-brown; elytra with contrasting, pale yellow to orange areas, humerus with pale crescent-shaped marking on disc, epipleuron broadly paler, usually with dark area in apical two thirds, apex of elytra broadly paler; dorsal abdomen with paratergites usually paler, tergite VII with pale, reddish-orange, semi-circular marking at apex; abdominal sternites III–V or III–VI with a pale, lateral spot at base and at apex; antennomeres I–V yellow-orange, VI–X brown to dark brown, XI distinctly paler, yellow, in some specimens antennomeres I and II slightly paler (yellow) than III–V; procoxa and profemur entirely pale, yellow, meso- and metacoxa darkened, metacoxa dark brown at base, meso- and metafemur with dark subapical band, tibia pale, yellow with darkened lateral face, tarsus pale, yellow brown.

Head with median frontal impression present as a pair of subparallel lines forming the anterior margin of central protuberance; frons relatively smooth, glossy with several coarse, asetose punctures, rarely more heavily sculptured though never rugose; with central protuberance, protuberance smooth, glossy, with several sparsely distributed, asetose punctures; base of head with well-developed posterior protuberances, surface smooth, glossy, with a few coarse, asetose punctures; microsculpture absent dorsally except as broken lines on frons and well-developed, fine lines on temples.

Pronotum with disc smooth, glossy, without or with very few, finely impressed micropunctures; lateral areas with moderately impressed, irregular, asetose punctures, often contiguous; protuberance distinct to pronounced in lateral view, less developed but still distinct in female; with two punctures in dorsal row; scutellum with several coarse, contiguous, deeply impressed, asetose punctures; elytra weakly to slightly transverse, suture shorter to as long as pronotum at midlength; macrosetae of elytra relatively long and erect, distinct from overall surface sculpture in lateral view.

Abdominal tergites with sparse, long and golden setae, III–VI impressed at base; disc of tergites III–VI impunctate medially, this area becoming smaller on successive tergites; sternites III–V with basal transverse line sharply projected posteriad at middle; abdominal sternites with relatively coarse microsculpture, interspaces wider than lines.

Median lobe in lateral view recurved dorsad, sometimes with sharp angle at position of recurvature, apical portion narrowed at this position to acute apex (Fig. 19E); median lobe in parameral view constricted near base, subparallel for most of its length and strongly, asymmetrically constricted into acute apex (Fig. 19D); paramere longer than median lobe, not divided but with median suture; in parameral view, constricted at midlength and then broadly but asymmetrically dilated, narrowed at apex (Fig. 19D); peg setae arranged in a pair of lateral, asymmetrical, longitudinal fields, with one field slightly broader at base relative to the other, about 7 peg setae across at the widest part of each field (Fig. 19F). Male sternite VII shallowly but distinctly emarginate and with glabrous area apicomedially, this area not flattened; male sternite VIII with transverse basal line broken medially, with slightly emarginate apex, impressed and glabrous in triangular area near emargination; male sternite IX distinctly asymmetrical at base, with moderately deep and broad emargination at apex (Fig. 24A).

Female tergite X broad, broadly rounded at obtuse apex, short, about half as long as laterotergal sclerites (Fig. 27A); female laterotergal sclerites not expanded at base and not overlapping with tergite X (c.f. Fig. 26K).

###### Distribution.

Figure 30C. Known only from Monteverde National Park but may occur elsewhere in the Tilarán Cordillera of Costa Rica.

###### Bionomics.

*Bolitogyrus tortifolius* has been collected in May and June, by FITs and on a white *Crepidotus* sp. fungus. Specimens have been found at elevations of 1515–1610 m.

###### Etymology.

The species epithet refers to the paramere of the aedeagus, which is similar to a curled leaf.

###### Comments.

Among species of overlapping distribution, *Bolitogyrus tortifolius* is most similar in habitus to *Bolitogyrus divisus* and *Bolitogyrus bullatus*. It can be easily distinguished by the pronotum with two punctures in the dorsal row. At present, the only male-associated female of *Bolitogyrus tortifolius* can be distinguished from females of *Bolitogyrus pseudotortifolius* by the shape of female tergite X, which is wider and more truncate at the apex than in the previous species.

##### Bolitogyrus pseudotortifolius

Taxon classificationAnimaliaColeopteraStaphylinidae

Brunke sp. n.

http://zoobank.org/062460A1-51D8-4697-A1E9-8B35DD0A1391

Figs 19G–J , 24B , 27B , 30C (map)

###### Type locality.

Panama, Chiriquí, La Fortuna.

###### Type material.

**Holotype** ♂ (SEMC): PANAMA: Chiriquí, La Fortuna, ‘Cont. Divide Trail’, 08°46'N, 82°12'W, 1150 m, 23 V- 9 VI 1995, J. Ashe, R. Brooks, #155, ex. flight intercept trap [white printed label] / SM0007041 [white barcode label] / Holotype, *Bolitogyrus pseudotortifolius* Brunke, sp. n. [red printed label].

**Paratypes** (18 ♂ 3 ♀ SEMC, PTC, ZMUC): same data as holotype, SM0007042, 1 ♂ (SEMC). **COSTA RICA:** *Cartago*, Santa Cruz, Guayabo, 1340 m, ex. fog logs in remnant, 24.IX.2000, P.N. Thomas, CR-1137, 1 ♂ (PTC); *Heredia*, 16 km SSE La Virgen, 1050-1150 m, ex. flight intercept trap, 10 to 24.IV.2001, 11/TN/18/028, ALAS-INBIO, 1 ♂ (PTC); same except: 21.III.2001, 11/TN/09/014, 3 ♂ (PTC); same except: 21.IV.2001, 11/TN/08/023, 1 ♂ (PTC); same except: 21.III.2001, 11/M/13/053, 1 ♀ (PTC); same except: 21.III.2001, 11/TN/18/018, 1 ♂ (PTC); same except: 21.III.2001, 11/TN/07/012, 1 ♂ (PTC); same except: 21.III.2001, 11/TN/06/001, 1 ♂ (PTC); same except: 11/TN/06/021, 21.IV.2001, 1 ♂ (PTC); same except: Malaise, 9.III to 21.IV.2001, 11/M/08/048, 1 ♂ (PTC); same except: Malaise, 21.III. to 9.IV.2001, 11/M/05/065, 1 ♂ (PTC). **PANAMA:** *Chiriquí*, La Fortuna, ‘Hydro trail’, 08°42'N, 82°14'W, 1150 m, ex. flight intercept trap, 23.V to 9.VI.1995, J. Ashe, R. Brooks, #156, SM0003733, SM0003738, SM0003741, SM0003743, 3 ♂ 1 ♀ (SEMC, ZMUC); same except: 21 to 23.V.1995, J. & A. Ashe, #051, SM0006090 SM0006093 SM0006095, 2♂ 1♀ (SEMC, ZMUC); La Fortuna, Continental Divide Trail, 8°46'0"N, 82°12'0"W, [1200 m, from SEMC database], ex. Berlese forest litter, 9.VI.1995, R. Anderson, PAN2A95 10E, SM0032118, 1 ♂ (SEMC).

###### Diagnosis.

Within the Bullatus Lineage: two punctures in the dorsal row of the pronotum (Fig. 7C); dark abdominal segment VIII; humerus with pale marking distinctly narrower than half the distance between scutellum and humeral angle (Fig. 10C); median lobe in lateral view slender, not recurved dorsad, with subapical tooth (Fig. 19H–I).

###### Description.

Measurements ♂ (n=5): HW/HL 1.68–1.78; PW/PL 1.59–1.71; EW/EL 1.16–1.28; ESut/PL 0.90–0.95; PW/HW 1.06–1.09; forebody length 3.5–3.7 mm.

Measurements ♀ (n=3): HW/HL 1.65–1.68; PW/PL 1.50–1.61; EW/EL 1.16–1.23; ESut/PL 0.95–0.96; PW/HW 1.00–1.03; forebody length 3.6–3.9 mm.

Extremely similar to *Bolitogyrus tortifolius* and differing only in the following: median lobe in lateral view slender, with subapical tooth and gradually narrowing to acute apex, which is produced ventrad, some specimens with weak flange on median lobe at midlength (Fig. 19H–I); median lobe in parameral view similar to *Bolitogyrus tortifolius* but asymmetry less pronounced (Fig. 19G); paramere similar to *Bolitogyrus tortifolius* but more slender, less asymmetrical; peg setae similar to *Bolitogyrus tortifolius* but fields narrower and more similar to each other, about 6 peg setae wide at the widest part of each field (Fig. 19J); male sternite IX with apex shallower and narrower (Fig. 24B); female tergite X shield-shaped, apex narrower and more truncate than in *Bolitogyrus tortifolius* (Fig. 27B).

###### Distribution.

Figure 30C. *Bolitogyrus pseudotortifolius* occurs along the Central and Talamanca Cordilleras in Costa Rica and Panama, on the Caribbean side of the continental divide.

###### Bionomics.

*Bolitogyrus pseudotortifolius* has been collected in March-June, at 1050-1200 m (once at 1340 m), by fogging logs and using passive traps (FIT, Malaise). The specimen from Cartago, Costa Rica is not included in the distribution map as it was collected from cut logs that appeared to have originated from elsewhere (P.N. Thomas, *pers. comm.*). One relatively pale specimen may be teneral and was collected by processing forest litter in a Berlese funnel. This specimen may indicate the litter layer as a site for pupation or even a larval habitat.

###### Etymology.

The specific epithet refers to the similarity of *Bolitogyrus pseudotortifolius* with *Bolitogyrus tortifolius*.

###### Comments.

Among species of overlapping distribution, *Bolitogyrus pseudotortifolius* is most similar in habitus to *Bolitogyrus falini* and *Bolitogyrus bullatus*. It can be easily distinguished by the pronotum with two punctures in the dorsal row. The available specimen data on *Bolitogyrus pseudotortifolius* and *Bolitogyrus tortifolius* suggest that these two species are allopatric and that, the former may be broadly distributed along the Caribbean foothills of the continental divide in Costa Rica and Panama.

#### Cornutus Group

**Figure 8.**
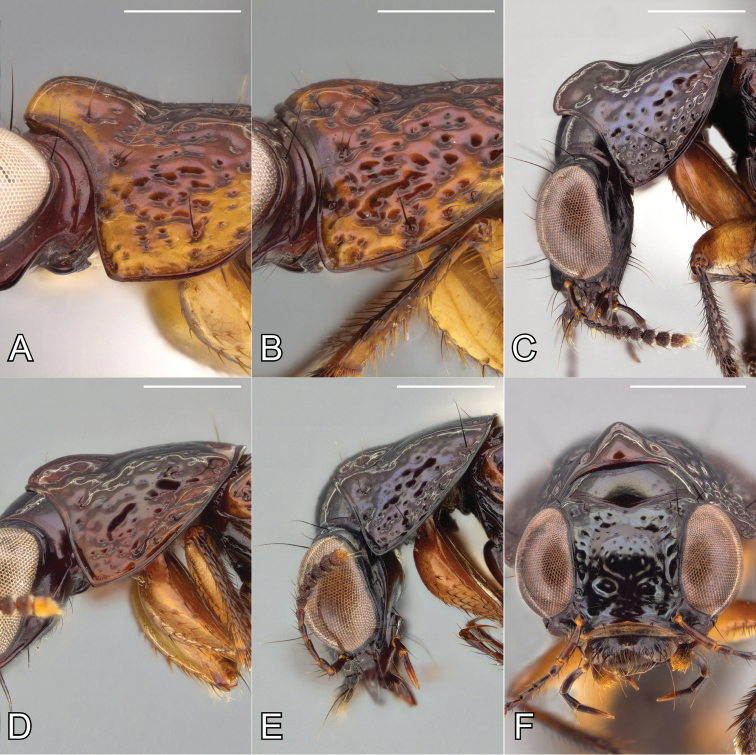
Pronotum, lateral: *Bolitogyrus bufo* Brunke, male (**A**), female (**B**); *Bolitogyrus cornutus* Brunke, male (**C**), ‘pseudofemale’ male (**D**), female (**E**). Head, frontal: *Bolitogyrus cornutus*, male (**F**). Scale bars = 0.5 mm (**A–B**), 1 mm (**C–F**).

The species of this group exhibit a marked sexual dimorphism of the pronotum in which the male pronotal protuberance is developed into a rounded or blunt ‘horn’ (Fig. 8A–F). Most females of species in the Cornutus Group can be identified as such by the pronotum with four punctures in the dorsal row and with lateral margins strongly converging anteriad. *Bolitogyrus cornutus* has only two punctures in the dorsal row but females can be identified to species by external characters given in the key and in the corresponding diagnosis below. The Cornutus Group is the most speciose group within the Bullatus Lineage and the only one to occur in South America. This species group is distributed from Costa Rica to Ecuador.

##### Bolitogyrus cornutus

Taxon classificationAnimaliaColeopteraStaphylinidae

Brunke sp. n.

http://zoobank.org/A1A53325-82DE-4E5D-A634-1101A9CD00A2

Figs 2B , 8C–F , 21A–D , 24E , 27D , 31A (map)

###### Type locality.

Ecuador, Pichincha, Mindo.

###### Type material.

**Holotype** ♂ (SEMC): ECUADOR: Pichincha, Mindo, 10.6 km, W. Mindo Road, 0°4'23"S, 78°45'14"W, 1375 m, 28 Mar 1999, R. Brooks, ECU1B99 061, ex. fungus covered log [white printed label] / SM0153248 [white barcode label] / Holotype, *Bolitogyrus cornutus* Brunke, sp. n. [red printed label].

**Paratypes** (5 ♂ 5 ♀, SEMC, ZMUC): same data as holotype, SM0153249, SM0153250, SM0153252, SM0153253, 3 ♂ 1 ♀ (SEMC, ZMUC). **COLOMBIA:** *Valle de Cauca*, PNN Farallones de Cali Anchicaya, 3°26'N, 76°48'W, 730 m, Malaise, 27.II to 27.III.2001, S. Sarria leg., M.1538, SM0548927, SM0548929, 1♂ 1♀ (SEMC); same locality except: 1.VIII to 10.X.2000, M.1104, SM0548928, 1 ♀ (SEMC); same locality except: 9.V to 18.VII.2000, M.1099, SM0548926, 1 ♀ (SEMC); same locality except: 8.V to 19.V.2001, M.1893, SM0548930, 1 ♂ (SEMC); same locality except: 900 m, 19.VI to 3.VII.2001, M.1891, SM0650434, 1 ♀ (SEMC).

###### Diagnosis.

Within the Bullatus Lineage: pronotum with two punctures in dorsal row (Fig. 7C); abdominal segment VIII pale yellow, distinctly paler than previous segments (Fig. 2B). Most male specimens can be recognized immediately by the protuberance of the pronotum produced into a truncate ‘horn’ (Fig. 8C).

###### Description.

Measurements ♂ (n=5): HW/HL 1.98–2.10 (‘pseudofemale’ with 1.90); PW/PL 1.66–1.85; EW/EL 1.26–1.32; ESut/PL 0.79–0.82; PW/HW 1.12–1.17; forebody length 3.8–4.3 mm.

Measurements ♀ (n=5): HW/HL 1.76–1.86; PW/PL 1.49–1.70; EW/EL 1.23–1.29; ESut/PL 0.86–0.96; PW/HW 1.04–1.07; forebody length 4.0–4.4 mm.

Coloration: body dark brown; frons sometimes with faint, green-blue metallic reflection; pronotum with faint bronze metallic reflection; pronotum with base narrowly, and lateral portions broadly and distinctly paler, orange-brown, pronotal protuberance paler, pale lateral areas sometimes joining with that on pronotal protuberance; elytra paler, reddish brown to yellowish-brown with epipleuron distinctly paler, yellowish, suture and outline of scutellum dark brown, apex of elytra indistinctly to noticeably paler; dorsal abdomen with tergites III–V reddish brown to red, bordered laterally and apically with contrasting dark brown in specimens from Ecuador, tergites more diffusely colored in Columbian specimens; tergites VI–VII darker, dark brown, apex of tergite VII with pale, orange semi-circular marking, apex of sternite VII pale, yellow; segment VIII and genital segment of both sexes entirely pale, yellow; antennomeres I–V pale, yellowish brown, segments I–II sometimes darker brown, antennomeres VI–X dark brown, apical antennomere slightly paler than previous, light-brown to brownish-yellow; maxillary and labial palpi yellow-brown, segments I–III darker in specimens from Ecuador; legs pale, yellow to yellowish brown, profemur without distinct dark subapical band, meso- and metafemur with dark subapical band nearly or entirely reaching apex, mid and hind coxa darkened, brownish, tibia with darkened lateral face, tarsus dark brown.

Head markedly more transverse in males than in females; with median frontal impression present and always distinct; frons coarsely sculptured but strongly glossy; base of head with well-developed posterior protuberances, surface smooth, glossy, with several coarse, sparsely distributed, asetose punctures; microsculpture absent dorsally except as broken lines on frons in some specimens and well-developed, fine lines on temples.

Pronotum markedly more transverse in males than in females; with disc smooth, glossy, with scattered fine to coarse micropunctures on disc, microsculpture absent except as broken lines on anterior angles; lateral areas with moderately impressed, irregularly spaced, asetose punctures, often contiguous; pronotal protuberance in lateral view distinct in both sexes but weakly to moderately developed in females (Fig. 8E), and strongly developed and truncate apically in males (Fig. 8C) (one male seen without strongly developed pronotal protuberance); lateral margins of pronotum strongly convergent anteriad in females and more weakly convergent anteriad in males but of a shape distinct from species of the Divisus Group (Fig. 2B); with two punctures in dorsal row; scutellum with 1-5 separated to contiguous, weakly impressed, asetose punctures; elytra weakly transverse, suture shorter than to nearly equal to pronotum at midlength; macrosetae of elytra relatively long and erect, distinct from overall surface sculpture in lateral view.

Median lobe entire, with more heavily sclerotized areas indicating two lateral lobes with subapical teeth (Fig. 21C); in lateral view, median lobe constricted at midlength, produced ventrad and then recurved dorsad (Fig. 21C); paramere reaching level of constriction of median lobe, in lateral view; in parameral view, median lobe slightly dilated in apical third, apex weakly to distinctly notched (Fig. 21A); paramere truncate to notched apically, narrowed at midlength and apical third dilated, without peg setae, apex with a row of fine setae (Fig. 21A–B, D). Male sternite VII without emargination, with only narrow glabrous area at middle; male sternite VIII with transverse basal line broken at middle, with slightly emarginate apex, impressed and glabrous in short triangular area near emargination; male sternite IX distinctly asymmetrical at base, with deep emargination at apex (Fig. 24E).

Female tergite X strongly constricted in basal half, base fused with accessory sclerite, with U-shaped emargination at apex (Fig. 27D); female laterotergal sclerites expanded at base and overlapping with tergite X (Fig. 27D).

###### Distribution.

Figure 31A. Known from the foothills of the Andes in Columbia and Ecuador. *Bolitogyrus cornutus* is the only species of the genus known to occur in South America and may occur farther north in Venezuela.

###### Bionomics.

Specimens of *Bolitogyrus cornutus* have been collected from elevations ranging from 700-1375 m, from fungus-covered logs and from Malaise traps during February to March and May to October.

###### Etymology.

The species epithet refers to the strongly developed pronotal protuberance of male *Bolitogyrus cornutus*. This species possesses the most strongly modified pronotum in the genus *Bolitogyrus*.

###### Comments.

*Bolitogyrus cornutus* is currently the only species of the genus known from South America and thus, can be recognized by distribution alone. One unassociated female (BMNH) from the vicinity of the type locality may belong to this species but, as it was collected at a substantially higher elevation (1700 m), remains unidentified.

The sexual dimorphism of the pronotum observed in this species is striking and the most pronounced in the entire genus (Fig. 8C–F). However, one male specimen from Columbia was observed with some female-associated characters including a pronotum with a small and rounded protuberance (Fig. 8D) and a less transverse head (1.90 versus 1.98–2.10 in other males). It is also smaller than the other males but size was not found to be sexually dimorphic in this species (see measurements). Its aedeagus is identical to that of other males from the same collecting event, suggesting that males of *Bolitogyrus cornutus* may be polymorphic for secondary sexual traits and possibly any behavior related to them.

##### Bolitogyrus brevistellus

Taxon classificationAnimaliaColeopteraStaphylinidae

Brunke sp. n.

http://zoobank.org/E66ADB39-8173-4E7B-BDAD-6F2F18D14331

Figs 21E–G , 24F , 30D (map)

###### Type locality.

Panama, Veraguas, Cerro Tute

###### Type material.

**Holotype** ♂ (SEMC): PANAMA: Veraguas, 6.1 km N of Sante Fé, Cerro Tute, 1210 m, 08°29'42"N, 81°7'0"W, 13 Jun 1996, J. Ashe, R. Brooks, PAN1AB96 133, ex. fungusy log [white printed label] / SM0043171 [white barcode label] / Holotype, *Bolitogyrus brevistellus* Brunke, sp. n. [red printed label].

**Paratype** (1 ♂, SEMC): **PANAMA:** Veraguas, 6.1 km N of Sante Fé, Cerro Tute, 1110 m, 8°30'30"N, 81°7'0"W, ex. fungusy log, 13.VI.1996, J. Ashe, R. Brooks, PAN1AB96 127, SM0043110, 1 ♂ (SEMC).

###### Diagnosis.

Within the Bullatus Lineage: pronotum with three punctures in dorsal row (c.f. Fig. 7D); lateral margins of pronotum strongly converging anteriad (c.f. Fig. 7C); male sternite VII only slightly emarginate; median lobe entire and distinctly narrowed in apical third (Fig. 21E); fields of peg setae on paramere restricted to apex (Fig. 21G).

###### Description.

Measurements ♂ (n=2): HW/HL 1.89–2.03; PW/PL 1.63–1.85; EW/EL 1.19–1.29; ESut/PL 0.88–0.92; PW/HW 1.11–1.19; forebody length 3.8–4.1 mm.

Similar to *Bolitogyrus cornutus* and differing only in the following: body paler, dark reddish brown, dorsal abdomen with tergites reddish brown, with (paratype) or without (holotype) dark brown area medially; abdominal segment VIII and genital segment of both sexes pale but tergite VIII with some apical darkening; apical antennomere not distinctly paler than previous; metafemur with dark subapical band far from reaching apex; frons slightly more densely sculptured; base of head with posterior protuberances less pronounced, flattened; male pronotal protuberance strongly developed, produced into a ‘horn’, but smaller than in males of *Bolitogyrus cornutus*, apex rounded not truncate; lateral margins of pronotum strongly convergent; with three punctures in dorsal row of pronotum; median lobe entire but apical portion notched and with median suture; in lateral view, median lobe slightly produced ventrad, narrowed at apical third, slightly expanded subapically, apex acute (Fig. 21F); in parameral view, median lobe strongly narrowed at apical third, converging slightly to narrow apex (Fig. 21E); paramere nearly reaching apex of median lobe; in lateral view, paramere slightly sinuate; paramere entire but with median suture and apex distinctly notched triangularly; in parameral view, paramere strongly narrowed at midlength and then dilated gradually toward notched apex (Fig. 21E); peg setae fields restricted to apex of paramere, present as a pair of short lateral clusters, 3-5 peg setae wide (Fig. 21G); male sternite VII with slight emargination and small, short, not flattened, glabrous area; male sternite VIII with transverse basal line not broken at middle, with slightly emarginate apex, impressed and glabrous in elongate, triangular area near emargination; male sternite IX with base more strongly asymmetrical, apex as narrow but not as deep (Fig. 24F). Female unknown.

###### Distribution.

Figure 30D. Known only from the type locality in Veraguas province, Panama.

###### Bionomics.

*Bolitogyrus brevistellus* has been collected from a fungusy log in June at an elevational range of 1110–1210 m.

###### Etymology.

The species epithet means ‘short constellation’ and refers to the relatively short fields of peg setae (like a constellation of stars) on the paramere, a character that separates it from the similar *Bolitogyrus longistellus*.

###### Comment.

As far as currently known, *Bolitogyrus brevistellus* is not sympatric with any species of the genus but is extremely similar to *Bolitogyrus longistellus* from Panama province, Panama. The forebody of *Bolitogyrus brevistellus* is distinctly paler than the holotype of *Bolitogyrus longistellus* but identifications should be based on dissected males until more specimens are available.

##### Bolitogyrus bufo

Taxon classificationAnimaliaColeopteraStaphylinidae

Brunke sp. n.

http://zoobank.org/AFAEAEDB-2AC2-4DF1-991D-11F6353E075F

Figs 2C , 8A–B , 14E , 21H–K , 24G , 27E , 30D (map)

###### Type locality.

COSTA RICA, Cartago, Tapantí National Park.

###### Type material.

**Holotype** ♂ (SEMC): COSTA RICA, Cartago, P.N. Tapanti, 1150 m, 9°45'41"N, 83°47'5"W [should be W], 18.VII.2000, J. Ashe, R. Brooks, Z. Falin, CR1AF00 166, ex. fogging fungus covered log [white printed label] / SM0203502 [white barcode label] / Holotype, *Bolitogyrus bufo* Brunke, sp. n. [red printed label].

**Paratypes** (37 ♂ 15 ♀, PTC, SEMC, ZMUC): same data as holotype, SM0203485, SM0203486, SM0203487, SM0203489, SM0203490, SM0203491, SM0203493, SM0203494, SM0203495, SM0203497, SM0203498, SM0203499, SM0203500, SM0203503, SM0203504, SM0207124 (11 ♂ 5 ♀ SEMC, ZMUC); **COSTA RICA:** *Cartago*, La Cangreja, Finca L. Brenes, Pan-Am. Hgwy km 29, 1590 m, ex. rotten logs, 30.III.1999, P.N. Thomas, CR # 1057, 5 ♂ 5 ♀ (PTC), same except: 1580 m, ex. fogging rotten logs, 23.X.1999, CR-1064, 1 ♂ 2 ♀ (PTC), same except: 1590 m, 20.III.2000, CR-1087, ex. fog rotten logs, 1 ♂ 2 ♀ (PTC); P.N. Tapantí, 9°43'12"N, 83°46'36"W, 1480 m, ex. fogging fungus covered log, 17.VII.2000, J. Ashe, R. Brooks, Z. Falin, CR1ABF00 163, SM0152900, SM0152901, 2 ♂ (SEMC, ZMUC); P.N. Tapantí, 9°45'41"N, 83°47'5"W, 1100 m, ex. fogging fungus covered log, 19.VII.2000, J. Ashe, R. Brooks, Z. Falin, CR1ABF00 176, SM0203306, 1 ♂ (SEMC); P.N. Tapantí, 1150 m, Malaise, III.1994, G. Mora, L N 194000_559800, #2864, SM0037997, 1 ♂ (SEMC); Refugio Nacional de Fauna Silvestre Tapantí, Sendero La Pava, 9°44.232'N, 83°46.820'W, 1400 m, ex. fungus covered log, 30.X.2001, R. Brooks, CR1B01 01, SM0474485, SM0474487, 2 ♂ (SEMC); Santa Cruz, Guayabo, Rio Lajitas, 1370 m, ex. fog log at falls, 23.XI.2000, P.N. Thomas, CR-1134, 3 ♂ 1 ♀ (PTC), same except: 1360 m, ex. fog logs, CR-1135, 1 ♂ (PTC); Santa Cruz, Guayabo, 1520 m, ex. fog log in remnant, 24.XI.2000, P.N. Thomas, CR-1137, 5 ♂ (PTC); *San Jose Province*, San Juan, 14 km NE, Finca Zurqui, 10°2'57"N, 84°0'22"W, 1490 m, ex. fogging fungus covered log, 6.VII.2000, J. Ashe, R. Brooks, Z. Falin, CR1ABF00 065, SM0203232, 1 ♂ (SEMC); Zurquí de Moravia, 1600 m, ex. malaise trap, 1 to 30.VI.1995, P. Hanson, CR1H93-95 11, SM0075350, 1 ♂ (SEMC), same except: 1 to 30.V.1995, CR1H93-95 12, SM0074231, 1 ♂ (SEMC), same except: 10°3'0"N, 84°1'0"W, 1 to 30.X.1995, CR1H95-96 02, SM106872, 1 ♂ (SEMC), same except: V.1994, SM0037989, 1 ♂ (SEMC).

###### Diagnosis.

Within the Bullatus Lineage: pronotum with three punctures in dorsal row (c.f. Fig. 7D); lateral margins of pronotum strongly converging anteriad (c.f. Fig. 7C); male sternite VII strongly emarginate but much wider than deep (Fig. 14E); median lobe entire and evenly converging to apex (Fig. 21H).

###### Description.

Measurements ♂ (n=5): HW/HL 1.75–2.10; PW/PL 1.56–1.84; EW/EL 1.25–1.35; ESut/PL 0.73–0.95; PW/HW 1.12–1.19; forebody length 3.6–4.3 mm.

Measurements ♀ (n=5): HW/HL 1.74–1.86; PW/PL 1.53–1.67; EW/EL 1.23–1.28; ESut/PL 0.85–0.94; PW/HW 1.04–1.14; forebody length 4.1–4.3 mm.

Similar to *Bolitogyrus cornutus* and differing only in the following: body much paler, pale to medium brown-orange to brown-yellow, without metallic reflections; paler portions of pronotum yellowish brown, pronotal protuberance only slightly paler in some specimens; elytra paler than in *Bolitogyrus cornutus*, reddish-brown to pale brown-yellow, epipleuron paler, yellow but less strongly contrasting with disc; dorsal abdomen with tergites III–V slightly paler than VI–VII, apex of tergite VII with linear, semi-circular or more triangular pale marking; tergite VIII pale, yellowish-brown but some specimens with lateral darkening; antennomeres I–V pale, yellowish brown, apical antennomere distinctly paler than and contrasting with previous segments, yellow to light yellow; profemur with faint to distinct, dark subapical band, meso- and metafemur with dark subapical band far from apex; frons finely and densely sculptured, less glossy than in *Bolitogyrus cornutus*; base of head with posterior protuberances less pronounced, flattened, their surface with coarse, asetose and often rugose punctures; microsculpture absent dorsally except as broken lines in depressions and near punctures and well-developed fine lines on temples; pronotal disc with moderately dense, coarse, asetose punctures and micropunctures, microsculpture absent except as broken lines in depressions and near punctures; lateral areas with denser punctation; pronotal protuberance well developed in lateral view and produced into a horn in males, smaller than that of *Bolitogyrus cornutus*, apex rounded not truncate, in females protuberance not produced into a horn but distinct; lateral margins of pronotum strongly convergent anteriad in both sexes; with three punctures in dorsal row; median lobe entire but apical portion with median suture and minutely notched in some specimens; in lateral view, median lobe slightly produced ventrad, narrowed at apical third, slightly expanded subapically, apex acute (Fig. 21I–J); in lateral view, median lobe with pair of flanges near apical third (Fig. 21I–J); paramere nearly reaching apex of median lobe; in lateral view, paramere slightly sinuate and following contour of median lobe; in parameral view, median lobe gradually narrowing from apical third to apex, lateral portions more strongly sclerotized and indicating potential traces of lateral lobes (Fig. 21H); paramere entire but with median suture, apical portion in parameral view with triangular notch and varying from entire to very narrowly divided at middle by suture (Fig. 21H); in parameral view, paramere narrow at middle, with lateral expansion at apical third, entire lateral portion of paramere strongly deflexed, such that base and middle portion on different plane (Fig. 21H); peg setae fields present as a pair of lateral rows extending from lateral extension of paramere to apex, broader at base and apex (Fig. K); male sternite VII with distinct but shallow emargination, much broader than deep, with broad and elongate, flattened, glabrous area (Fig. 14E); male sternite VIII with transverse basal line not broken at middle, with apex slightly more emarginate than in *Bolitogyrus cornutus*, impressed and glabrous in elongate, triangular area near emargination; male sternite IX with base more strongly asymmetrical, apex as narrow but not as deep (Fig. 24G); female tergite X strongly constricted in basal half but slightly narrower in shape than in *Bolitogyrus cornutus*, base fused with accessory sclerite, with widely concave apex (Fig. 27E); female laterotergal sclerites more greatly expanded at base and overlapping with tergite X (Fig. 27E).

###### Distribution.

Figure 30D. This species is known only from Costa Rica, from Cartago and San Jose provinces in the Central Cordillera, and along the northern slopes of the Talamanca Cordillera facing the Central Cordillera.

###### Bionomics.

*Bolitogyrus bufo* has been collected at elevations ranging from 1150-1600 m, using flight traps and by fogging fungus covered logs. Collections are known from March, May-July and October-November.

###### Etymology.

The species epithet is the Latin word for toad. It refers to the similarity between the roughly sculptured, light brown cuticle, typical of *Bolitogyrus bufo* and the rough, warty skin of a true toad (Bufonidae). It is to be used as a noun in apposition.

###### Comments.

Within its distribution, *Bolitogyrus bufo* is not easily confused with any other species due to the pale coloration and coarse sculpture. It is extremely similar to *Bolitogyrus thomasi* from the Caribbean side of the continental divide in Heredia, Costa Rica but can be distinguished by the differently shaped emargination of male sternite VII and the differently shaped paramere.

##### Bolitogyrus cheungi

Taxon classificationAnimaliaColeopteraStaphylinidae

Brunke sp. n.

http://zoobank.org/184EB671-F4C1-472C-A743-0B5FCA6481ED

Figs 21L–N , 24H , 27F , 31A (map)

###### Type locality.

Panama, Darién, Cana Biological Station.

###### Type material.

**Holotype** ♂ (SEMC): PANAMA: Darién, Cana Biological Station, Serranía de Pirre, 1250 m, 7°45'18"N, 77°41'6"W, 05 Jun 1996, J. Ashe, R. Brooks, PAN1AB96 042, ex. fungusy log, [white printed label] / SM0013883 [white barcode label] / Holotype, *Bolitogyrus cheungi* Brunke, sp. n. [red printed label].

**Paratypes** (1 ♂ 1 ♀, SEMC): same as holotype except: 1200 m, PAN1AB96 039, SM0013882, 1 ♂ (SEMC), same except: 1350 m, 7.VI.1996, PAN1AB96 102, SM0043435, 1 ♀ (SEMC).

###### Diagnosis.

Within the Bullatus Lineage: pronotum with three punctures in dorsal row (c.f. Fig. 7D); lateral margins of pronotum strongly converging anteriad (c.f. Fig. 7C); male sternite VII not distinctly emarginate; median lobe divided into a pair of ventrally curved lateral lobes, these lobes without teeth (Fig. 21L).

###### Description.

Measurements ♂ (n=2): HW/HL 1.89–2.00; PW/PL 1.58–1.69; EW/EL 1.25–1.32; ESut/PL 0.80–0.84; PW/HW 1.13–1.23; forebody length 3.8–3.9 mm.

Measurements ♀ (n=1): HW/HL 1.78; PW/PL 1.57; EW/EL 1.32; ESut/PL 0.87; PW/HW 1.04; forebody length 3.7 mm.

Similar to *Bolitogyrus cornutus* and differing only in the following: body without metallic reflections; dorsal abdomen reddish brown with darker brown paratergites, tergites VI–VII slightly darker; segment VIII pale, yellow but dark brown laterally; genital segment of both sexes dark, brownish; all femora with dark subapical band far from apex; frons finely and densely sculptured, less glossy than in *Bolitogyrus cornutus*; base of head with posterior protuberances less pronounced, flattened, their surface with coarse, asetose and often rugose punctures; microsculpture absent dorsally except as broken lines in depressions and near punctures and well-developed fine lines on temples; pronotal disc with moderately dense, coarse, asetose punctures and micropunctures, microsculpture absent except as broken lines in depressions and near punctures; lateral areas with denser punctation; pronotal protuberance well developed and produced into a horn in males, smaller than that of *Bolitogyrus cornutus*, apex rounded not truncate, one male with protuberance smaller than that of holotype male but still larger than that of female paratype, female protuberance not produced into a horn but distinct; lateral margins of pronotum strongly convergent anteriad in both sexes; with three punctures in dorsal row; median lobe divided apically into two lateral lobes without subapical teeth (Fig. 21L); in lateral view, median lobe gradually narrowed to subapex of lateral lobe, lateral lobes produced ventrad and with expanded, truncate apex (Fig. 21M); paramere slightly sinuate and reaching subapex of lateral lobe; in parameral view, median lobe subparallel until division into lateral lobes, lateral lobes slightly divergent and curving inward at apex (Fig. 21L); lateral lobes in parameral view separated by a broad, rounded emargination (Fig. 21L); paramere in parameral view dilated from midlength to subapex, then sharply narrowed, the following portion gradually narrowed to acute apex (Fig. 21L); paramere with pair of broad, lateral peg setae fields following apical and subapical margins (Fig. 21N); male sternite IX more strongly asymmetrical at base than in *Bolitogyrus cornutus*, with shallower emargination at apex (Fig. 24H); female tergite X strongly reduced, ‘anchor-shaped’, with slender base and broadly rounded apex, without accessory sclerite but basal margin of laterotergal sclerites fused across base of tergite X (Fig. 27F); female laterotergal sclerites expanded at base, fused together at middle and entirely covering most of tergite X (Fig. 27F).

###### Distribution.

Figure 31A. Known only from the type locality in Darién province, Panama.

###### Bionomics.

*Bolitogyrus cheungi* has been collected from fungusy logs at an elevational range of 1200–1350 m in June.

###### Etymology.

The first author dedicates this species to his partner, David K.-B. Cheung. His support and understanding during this project, while away from home and family, were greatly appreciated.

###### Comments.

The relatively small pronotal protuberance of the male paratype compared to that of the holotype may indicate some interesting polymorphism in this trait, similar to that of *Bolitogyrus cornutus* (see discussion under ‘Natural History’) but too few specimens are known to confirm this. *Bolitogyrus cheungi* is not sympatric with any other known species of *Bolitogyrus* but is most similar to *Bolitogyrus longistellus*. At present, it can only be reliably separated by the distinctively bilobed aedeagus. The lateral lobes of the aedeagus in *Bolitogyrus cheungi* differ in shape from those of the Divisus Group and it is uncertain whether they are homologous to those of the latter. Phylogenetic analyses using a variety of morphological character systems may resolve this in the future and ascertain whether the Divisus and Cornutus Groups are natural lineages.

##### Bolitogyrus gracilis

Taxon classificationAnimaliaColeopteraStaphylinidae

Brunke sp. n.

http://zoobank.org/4CDF1A6D-9EDA-4267-B50C-ED2D425CDC61

Figs 22A–D , 24I , 30D (map)

###### Type locality.

Panama, Chiriquí, La Fortuna.

###### Type material.

**Holotype** ♂ (SEMC): PANAMA: Chiriquí, La Fortuna, “Hydro Trail”, 08°42'N, 82°14'W, 1150 m, 9.VI.1995, J. Ashe, R. Brooks, #164, ex. fungusy log [white printed label] / SM0002814 [white barcode label] / Holotype, *Bolitogyrus gracilis* Brunke, sp. n. [red printed label].

**Paratypes** (11 ♂ 1 ♀, SEMC, ZMUC): **PANAMA:** Chiriquí, La Fortuna, “Rio Hornito Trail”, 8°42'N, 82°14'W, 1000 m, ex. fogging fungusy log, 11.VI.1995, J. Ashe & R. Brooks, #175, SM0009068, 1♂ (SEMC), same except: “Quebrada Al. Trail”, 1250 m, #180, SM0015976, SM0004548, 2 ♂ (SEMC); Chiriquí, La Fortuna, “Cont. Divide Trail”, 08°46'N, 82°12'W, 1100 m, ex. fungusy log, 23.V.1995, J. & A. Ashe, #046, SM0000357, SM0000364, 2 ♂ (SEMC), same except: 1150 m, ex. fungusy log, 9.VI.1995, J. Ashe & R. Brooks, #150, SM0001353, SM0001357, SM0002394, SM0002396, SM0002397, SM0003164, SM0002398, 6♂ 1♀ (SEMC, ZMUC).

###### Diagnosis.

Within the Bullatus Lineage: pronotum with three punctures in dorsal row (c.f. Fig. 7D); lateral margins of pronotum strongly converging anteriad (c.f. Fig. 7C); male sternite VII not distinctly emarginate; median lobe entire and evenly converging to apex (Fig. 22A).

###### Description.

Measurements ♂ (n=5): HW/HL 1.86–2.03; PW/PL 1.64–1.77; EW/EL 1.12–1.25; ESut/PL 0.77–0.93; PW/HW 1.10–1.14; forebody length 3.4–3.9 mm.

Measurements ♀ (n=2): HW/HL 1.78–1.79; PW/PL 1.59–1.62; EW/EL 1.28–1.32; ESut/PL 0.94–0.96; PW/HW 1.00–1.01; forebody length 3.78–4.03 mm.

Similar to *Bolitogyrus cornutus* and differing only in the following: body much paler, pale to medium brown-orange to brown-yellow, without metallic reflections; paler portions of pronotum yellowish brown, pronotal protuberance only slightly paler in some specimens; elytra paler than in *Bolitogyrus cornutus*, reddish-brown to pale brown-yellow, epipleuron paler, yellow but less strongly contrasting with disc; dorsal abdomen with tergites III–V slightly paler than VI–VII, apex of tergite VII with linear, semi-circular or more triangular pale marking; tergite VIII pale, yellowish-brown but some specimens with lateral darkening; antennomeres I–V pale, yellowish brown; profemur with dark subapical band, meso- and metafemur with dark subapical band far from apex; frons generally more finely and densely sculptured; base of head with posterior protuberances less pronounced, flattened, their surface with coarse, asetose, often rugose punctures; microsculpture absent dorsally except as broken lines in depressions and near punctures, and well-developed fine lines on temples; pronotal disc with moderately dense, coarse, asetose punctures and micropunctures, microsculpture absent except as broken lines in depressions and near punctures; lateral areas with denser punctation; pronotal protuberance well developed and produced into a horn in males, smaller than that of *Bolitogyrus cornutus*, apex rounded not truncate, in females not produced into a horn but distinct; lateral margins of pronotum strongly convergent anteriad in both sexes; with three punctures in dorsal row; median lobe entire but apical portion with median suture and minutely notched in some specimens; in lateral view, median lobe slightly produced ventrad, narrowed at apical third and more gradually to acute apex (Fig. 22C); in lateral view, paramere nearly reaching apex of median lobe, slightly sinuate and following contour of median lobe; in parameral view, median lobe gradually narrowing from apical third to apex, lateral portions more strongly sclerotized and indicating potential traces of lateral lobes (Fig. 22A); paramere entire but with median suture; in parameral view, paramere narrow at middle, with lateral expansion at apical third (Fig. 22A–B); peg setae fields present as a pair of thin (3-5 peg setae) lateral rows extending from maximum lateral extension of paramere to apex, broader at apex (Fig. 22D); male sternite VII with distinct triangular glabrous area; male sternite VIII with transverse basal line not broken at middle, with apex slightly more emarginate than in *Bolitogyrus cornutus*, impressed and glabrous in deeper triangular area near emargination; male sternite IX with base more strongly asymmetrical, apex as narrow but not as deep (Fig. 24I); female tergite X strongly constricted in basal half but slightly narrower in shape than in *Bolitogyrus cornutus*, base fused with accessory sclerite, with U-shaped emargination at apex (c.f. Fig. 27E); female laterotergal sclerites more greatly expanded at base and overlapping with tergite X (Fig. 27E).

###### Distribution.

Figure 30D. Known only from the type locality in Chiriquí province, Panama but probably occurs in adjacent Costa Rica.

###### Bionomics.

*Bolitogyrus gracilis* has been collected from fungusy logs at an elevational range of 1000-1250 m in May and June.

###### Etymology.

The species epithet means ‘slender’ and refers to the slender habitus created by the relatively long and thin abdomen, especially of male specimens.

###### Comments.

*Bolitogyrus gracilis* is not sympatric with any species that could be confused with it due to the pale coloration and coarse sculpture but is most similar in habitus to *Bolitogyrus bufo*, *Bolitogyrus thomasi*, *Bolitogyrus brevistellus* and *Bolitogyrus cheungi*. Without distributional information, males of *Bolitogyrus gracilis* should be dissected to confirm their identity.

##### Bolitogyrus longistellus

Taxon classificationAnimaliaColeopteraStaphylinidae

Brunke sp. n.

http://zoobank.org/B156BBFC-29FC-44A7-B573-43B59EB648DD

Figs 22E–F , 24J , 30D (map)

###### Type locality.

Panama, Panamá, km 7.5 El Llano-Cartí Road.

###### Type material.

**Holotype** ♂ (SEMC): PANAMA: Panamá, km 7.5 El Llano-Cartí Road, 9°13'N, 79°05'W [in error, correct is 9.27, -78.96], 14 June 1996, 400 m [370 m in SEMC database], A. Gillogly [white printed label] / SM0016620 [white barcode label] / Holotype, *Bolitogyrus longistellus* Brunke, sp. n. [red printed label].

###### Diagnosis.

Within the Bullatus Lineage: pronotum with three punctures in dorsal row (c.f. Fig. 7D); lateral margins of pronotum strongly converging anteriad (c.f. Fig. 7C); male sternite VII not distinctly emarginate; median lobe entire and distinctly narrowed in apical third (Fig. 22E); peg setae of paramere in elongate fields (Fig. 22F).

###### Description.

Measurements ♂ (n=1): HW/HL 2.14; PW/PL 1.67; EW/EL 1.32; ESut/PL 0.76; PW/HW 1.16; forebody length 3.9 mm.

Similar to *Bolitogyrus cornutus* and differing only in the following: dorsal abdomen with tergites dark brown, paler apically; abdominal segment VIII and genital segment pale but tergite VIII with some basal darkening; metafemur with dark subapical band far from reaching apex; frons slightly more densely sculptured; base of head with posterior protuberances less pronounced, flattened; male pronotal protuberance strongly developed, produced into a ‘horn’, but smaller than in males of *Bolitogyrus cornutus*, apex rounded not truncate; lateral margins of pronotum strongly convergent; with three punctures in dorsal row of pronotum; median lobe entire but apical portion notched and very narrowly divided by median suture; in lateral view, median lobe slightly produced ventrad, narrowed to acute apex; paramere nearly reaching apex of median lobe; in lateral view, paramere slightly sinuate; in parameral view, median lobe strongly narrowed at apical third, converging slightly to narrow apex (Fig. 22E); paramere entire but with median suture, apex distinctly notched triangularly; in parameral view, paramere narrowed at midlength, dilated gradually toward to subapex, then gradually narrowed to notched apex (Fig. 22E); peg setae fields present as a pair of lateral rows 3-5 peg setae wide, widest basally (Fig. 22F); male sternite VII with small, very short, not flattened, glabrous area; male sternite VIII with transverse basal line not broken at middle, with slightly emarginate apex, impressed and glabrous in elongate, triangular area near emargination; male sternite IX with base more strongly asymmetrical, apex as narrow but not as deep (Fig. 24J). Female unknown.

###### Distribution.

Figure 30D. Known only from the type locality in Panamá province, Panama.

###### Bionomics.

The holotype was collected in tropical rainforest at 370 m in June.

###### Etymology.

The species epithet means ‘long constellation’ and refers to the relatively long fields of peg setae (like a constellation of stars) on the paramere, a character that separates it from the similar *Bolitogyrus brevistellus*.

###### Comments.

As far as known, *Bolitogyrus longistellus* is not sympatric with any other species of *Bolitogyrus* but is most similar in habitus to other members of the Cornutus Group. Without distributional information, it can be reliably separated only by the characteristic paramere.

##### Bolitogyrus thomasi

Taxon classificationAnimaliaColeopteraStaphylinidae

Brunke sp. n.

http://zoobank.org/24ABD9CD-8BD3-481F-98C2-B08CE1F1A676

Figs 14F , 22G–I , 24K , 30D (map)

###### Type locality.

COSTA RICA, Heredia, 16 km SSE of La Virgen.

###### Type material.

**Holotype** ♂ (PTC): COSTA RICA, Heredia, 16 SSE La Virgen, 1050-1150 MTS. [white printed label] / ex. flight intercept, ‘11/TN/06/001’, 21/Mar/01, ALAS-INBIO [white printed label] / Holotype, *Bolitogyrus thomasi* Brunke, sp. n. [red printed label].

###### Diagnosis.

Within the Bullatus Lineage: pronotum with three punctures in dorsal row (c.f. Fig. 7D); lateral margins of pronotum strongly converging anteriad (c.f. Fig. 7C); male sternite VII strongly emarginate and about as deep as wide; median lobe entire and evenly converging to apex (Fig. 22G).

###### Description.

Measurements ♂ (n=1): HW/HL 2.05; PW/PL 1.63; EW/EL 1.27; ESut/PL 0.76; PW/HW 1.13; forebody length 3.9 mm.

Similar to *Bolitogyrus cornutus* and differing only in the following: body much paler, pale to medium brown-orange, without metallic reflections; paler portions of pronotum yellowish brown, pronotal protuberance only slightly paler; elytra paler than in *Bolitogyrus cornutus*, reddish-brown, epipleuron paler, yellow but less strongly contrasting with disc; dorsal abdomen with tergites III–V slightly paler than VI–VII, apex of tergite VII with nearly linear, pale marking; tergite VIII pale, yellowish-brown but with lateral darkening; antennomeres I–V pale, yellowish brown, apical antennomere distinctly paler than and contrasting with previous segments, yellow to light yellow; profemur with distinct, dark subapical band, meso- and metafemur with dark subapical band far from apex; frons finely and densely sculptured, less glossy than in *Bolitogyrus cornutus*; base of head with posterior protuberances less pronounced, flattened, their surface with coarse, asetose and often rugose punctures; microsculpture absent dorsally except as broken lines in depressions and near punctures and well-developed fine lines on temples; pronotal disc with moderately dense, coarse, asetose punctures and micropunctures, microsculpture absent except as broken lines in depressions and near punctures; lateral areas with denser punctation; pronotal protuberance well developed and produced into a horn in males, smaller than that of *Bolitogyrus cornutus*, apex rounded not truncate; lateral margins of pronotum strongly convergent anteriad; with three punctures in dorsal row; median lobe entire but apical portion with median suture and minutely notched; in lateral view, median lobe slightly produced ventrad, narrowed to acute apex, apical portion with slight flange (Fig. 22H); paramere nearly reaching apex of median lobe; in parameral view, median lobe gradually narrowing from apical third to apex, lateral portions more strongly sclerotized and indicating potential traces of lateral lobes (Fig. 22G); paramere entire but with median suture, apical portion in parameral view with triangular notch and very narrowly divided at middle by suture for a short distance; in parameral view, paramere subparallel distal from base, gradually dilated to apex in apical fourth (Fig. 22G); peg setae fields present as a pair of lateral rows of nearly uniform width, extending from apical fourth to apex (Fig. 22I); male sternite VII with distinct and deep emargination, about as deep as broad, with broad and elongate, flattened, glabrous area (Fig. 14F); male sternite VIII with transverse basal line broken at middle, with apex slightly more emarginate than in *Bolitogyrus cornutus*, impressed and glabrous in elongate, triangular area near emargination; male sternite IX with base more strongly asymmetrical, apex as narrow but not as deep (Fig. 24K). Female unknown.

###### Distribution.

Figure 30D. Known only from the type locality on the Caribbean foothills of Barva Volcano in the Central Cordillera, Costa Rica.

###### Bionomics.

The holotype was captured by a flight intercept trap at 1050-1150 m in March.

###### Etymology.

The first author would like to dedicate this species to his colleague Paul N. Thomas (Chicago, Illinois, U.S.A.), whose collecting expeditions in Costa Rica resulted in a significant and dominant portion of the material studied for the present revision.

###### Comments.

*Bolitogyrus thomasi* is most similar to other species of the Cornutus Group, especially *Bolitogyrus bufo*. However, none of these species are sympatric with it. Without distributional information, males may be identified by the deep emargination on sternite VII, which is about as deep as wide.

#### Divisus Group

The three members of this group (*Bolitogyrus divisus*, *Bolitogyrus falini* and *Bolitogyrus inexspectatus*) share the following characters: lateral margins of pronotum only weakly convergent anteriad, margins nearly straight in anterior half (Fig. 7D); median lobe divided into two slender arms each bearing a subapical tooth (e.g., Fig. 20A). The median lobe of *Bolitogyrus cornutus* (Cornutus Group), while entire, does show an indication of the two arms with subapical teeth, traced by faint lines. However, this species was placed with other species of the Cornutus Group due to characters on the pronotum (see Cornutus Group). The Divisus Group is endemic to Costa Rica as far as known.

##### Bolitogyrus divisus

Taxon classificationAnimaliaColeopteraStaphylinidae

Brunke sp. n.

http://zoobank.org/611E7432-A1EC-4D98-ABEB-0BC89C0CD436

Figs 2A , 7D, G, H , 14C , 20A–G , 24C , 27C , 32A , 31B (map)

###### Type locality.

COSTA RICA, Alajuela, 27 km N & 8 km W San Ramon.

###### Type material.

**Holotype** ♂ (SEMC): COSTA RICA, Alajuela, E.B. San Ramon, R.B. San Ramon, 27 km N & 8 km W San Ramon, 810 m, 10°13'4"N, 84°35'46"W, 8 JUL 2000, J. Ashe, R. Brooks, Z. Falin, CR1ABF00 076, ex. fogging fungus covered log [white printed label] / SM0203230 [white barcode label] / Holotype, *Bolitogyrus divisus* Brunke, sp. n. [red printed label].

**Paratypes** (45 ♂ 19 ♀, INBIO, PTC, SEMC, ZMUC): same data as holotype, SM0203231, 1 ♂ (SEMC), same except: 7.VII.2000, SM0203271, SM0203272, SM0203273, SM0203276, SM0203277, 4 ♂ 1 ♀ (SEMC). **COSTA RICA:** *Alajuela*, Peñas Blancas, 870 m, ex. Ascomycete, 19.V.1989, J. Ashe, R. Brooks, R. Leschen, SM0037919, 1 ♂ (SEMC), same except 970 m, ex. flight intercept trap, 20.V.1989, SM0037918, 1 ♂ (SEMC); Rincon Rainforest, Bioforestal Bustos, 860 m, ex. FIT-5, 2 to 5.IV.2002, P.N. Thomas, CR-1272, 1 ♀ (PTC), same except FIT-4, 820 m, 2 to 5.IV. 2001, CR-1271, 2 ♂ (PTC); Upala District, P. N. Volcan Tenorio, Cerro La Carmela, 1026 m, Malaise, 18.VII to 18.VIII.2010, J. A. Azofeifa, L_N_298828_427338 #99727, INB0004253815, 1 ♀ (INBIO); Rio Sn. Lorencito, Res. For. Sn. Ramon, 5 km N Col. Palmarena, 900 m, III.1990, ‘Curso Carabidae’, 244500-470700, CR1000202352, 1 ♂ (ZMUC); *Cartago*, Guayabo National Park, 1060 m, secondary forest, ex. fog logs, 23.XI.2000, CR-1133, P.N. Thomas, 4 ♂ 2 ♀ (PTC). *Guanacaste*, Cacao Station, SW side Volcan Cacao, 1000-1400 m, X.1989, R. Blanco & C. Chavez, URCG, 323300, 375700, CR1000097076, 1 ♂ (INBIO), same except: VII.1989 to III.1990, CR1000248460, 1 ♂ (INBIO), same except: Malaise, VII.1989 to III.1990, L-N-375700, CR1000248491, 1 ♂ (INBIO); Guanacaste, Pitilla Biological Station, 10°59'22"N, 85°25'33"W, 900 m, ex. fogging fungus covered log, 13.VII.2000, J. Ashe, R. Brooks, Z. Falin, CR1ABF00 122, SM0198817, 1 ♂ (SEMC), same except: 580 m, 14.VII.2000, CR1ABF00 133, SM023302, 1 ♀ (SEMC), same except: 610 m, 14.VII.2000, SM0203303, 1 ♂ (SEMC); Guanacaste National Park, Pitilla Station, 9 km S Sta. Cecilia, 700 m, Malaise, 1991, L-N 330200, 380200, CR1000853086, 1 ♀ (INBIO); Guanacaste National Park, Pitilla Station, 690 m, ex. fogged logs, 21.III.2001, CR-1233, P.N. Thomas, 2 ♂ 3 ♀ (PTC), same except: Memos trail, 580-690 m, 22.I.2001, CR-1175, 10 ♂ 1 ♀ (PTC), same except: 720 m, ex. fogged tree, 21.III.2001, CR-1234, 2 ♂ 2 ♀ (PTC), same except: Montecele trail, 660 m, 23.III.2001, CR-1250, 1 ♂ 1 ♀ (PTC); Rincon de la Vieja, Pailas Crater trail, 1180 m, ex. fog wet logs, 1.II.2001, P.N. Thomas, CR-1210, 2 ♂ 1 ♀ (PTC); San Cristobal Station, Danta trail, 870 m, ex. fogged log, 4.IV.2001, P.N. Thomas, CR-1275, 8 ♂ (PTC), same except: 840 m, ex. flight intercept, CR-1262, 31.III to 4.IV.2001, 1 ♂ (PTC). *Puntarenas*, Monteverde Biological Reserve, Est. G. Brenes, 1300 m, VI.1991, E. Belio, L-N-249750, 450075, CR1000567249, CR1000567250, 1 ♂ 1 ♀ (INBIO); Monteverde, ex. flight intercept trap, 29 to 31.VI.1992, M.L. Jameson, SM0038005, 1 ♂ (SEMC); Monteverde, 1450 m, ex. polypore fungi, 15.V.1989, J. Ashe, R. Brooks, R. Leschen, SM0038013, 1 ♀ (SEMC), same except: 1400 m, ex. under logs and on fungi, 5.V.1989, #024, SM0038006, 1 ♀ (SEMC); San Luis, Monteverde, A.C. Arenal, 1040 m, Malaise, set 1993, Z. Fuentes, L N 250850_449250 #2429, CRI002523060, 1 ♀ (INBIO).

###### Diagnosis.

Within the Bullatus Lineage: three punctures in the dorsal row of the pronotum (Fig. 7D); lateral margins of pronotum only weakly convergent anteriad, sides nearly straight in anterior half (Fig. 7D); genital segment of both sexes bright yellow to orange, contrasting with previous segments (Fig. 14C).

###### Description.

Measurements ♂ (n=5): HW/HL 1.68–1.82; PW/PL 1.49–1.55; EW/EL 1.22–1.28; ESut/PL 0.79–0.88; PW/HW 1.04–1.07; forebody length 4.0–4.3 mm.

Measurements ♀ (n=5): HW/HL 1.58–1.67; PW/PL 1.44–1.47; EW/EL 1.22–1.36; ESut/PL 0.72–0.86; PW/HW 1.01–1.06; forebody length 4.2–4.6 mm.

Coloration: Body dark brown, portions of frons occasionally with faint green-blue metallic reflection, pronotum with faint bronze metallic reflection; pronotum with base narrowly, and lateral portions broadly and distinctly paler, dark orange, pronotal protuberance often paler, pale lateral areas sometimes joining with that on pronotal protuberance; elytra reddish brown with epipleuron distinctly paler, yellowish, apex of elytra indistinctly paler; dorsal abdomen with segments III–V reddish brown, VI–VII darker, dark brown, tergite VII with variably shaped, apical, orange marking, tergite VIII usually entirely pale, yellow, sometimes yellow at base and dark at apex; genital segment of both sexes pale, yellow; antennomeres I–V pale, yellowish brown, some segments sometimes slightly darker, antennomeres VI–X dark brown, apical antennomere distinctly paler than previous, yellow to pale yellowish-white; maxillary and labial palpi yellow-brown; legs yellow to yellowish brown, femur with dark subapical band, profemur with dark band weakly formed, nearly absent on some specimens, tibia with darkened lateral face.

Head with median frontal impression present as a pair of subparallel lines forming the anterior margin of central protuberance, impression often obscured by sculpture; frons relatively coarsely sculptured, slightly glossy; base of head with well-developed posterior protuberances, surface smooth, glossy, with several coarse, sparsely distributed, asetose punctures; microsculpture absent dorsally except as broken lines on frons and well-developed, fine lines on temples.

Pronotum with disc smooth, glossy, with scattered fine to coarse micropunctures on disc; lateral areas with moderately impressed, irregularly spaced, asetose punctures, often contiguous; pronotal protuberance distinct in lateral view but slightly less pronounced in females; lateral margins of pronotum only weakly convergent anteriad, sides nearly straight in anterior half; with three punctures in dorsal row; scutellum with a few separated, moderately impressed, asetose punctures; elytra slightly to weakly transverse, suture shorter than pronotum at midlength; macrosetae of elytra relatively long and erect, distinct from overall surface sculpture in lateral view.

Median lobe divided apically into two lateral lobes; in lateral view, lateral lobes produced ventrad from their base, with subapical tooth on dorsal face, tooth conical and not flattened, apex of lateral lobe not flanged, evenly cylindrical (Fig. 17E; 32A); lateral lobes in parameral view convergent apically, touching or not, forming acute to broadly rounded emargination at their base (Fig. 17A); paramere distinctly shorter than median lobe, at most, slightly divided apically but always with median suture (Fig. 17A); in parameral view, paramere constricted at midlength, strongly dilated and then constricted subapicad, narrowed very slightly to apex (Fig. 17C–D); apical portion of paramere with sides subparallel or divergent, apex blunt; with peg setae in a pair of lateral fields, strongly broadened at base, narrowed for the majority of their length and slightly widened at apex (Fig. 17F–G). Male sternite VII shallowly but distinctly emarginate and with glabrous area apicomedially, this area slightly to strongly flattened; male sternite VIII with transverse basal line complete medially, with slightly emarginate apex, impressed and glabrous in elongate triangular area near emargination; male sternite IX distinctly asymmetrical at base, with moderately deep emargination at apex (Fig. 24C).

Female tergite X strongly constricted in basal half, evenly rounded at moderately narrow apex, base of female tergite X fused with accessory sclerite formed from the expanded basal margin of the laterotergal sclerites (Fig. 27C); female laterotergal sclerites strongly expanded at base and overlapping with tergite X (Fig. 27C).

###### Distribution.

Figure 31B. This species occurs along the Guanacaste, Tilarán and Central Cordilleras.

###### Bionomics.

This species has been collected at elevations ranging from 810-1450 m in flight traps, on fungi and by fogging fungus covered logs. Specimens have been collected in January-August and in October.

###### Etymology.

The species epithet refers to the division of the median lobe into a pair of lateral lobes bearing subapical teeth, a character unique to the Divisus Group of *Bolitogyrus* within the genus and the entire tribe Staphylinini.

###### Comments.

Among species with an overlapping distribution, *Bolitogyrus divisus* is most similar to *Bolitogyrus bullatus* and *Bolitogyrus tortifolius* but is easily distinguished by the pronotum with three punctures in the dorsal row. *Bolitogyrus divisus* is extremely similar to *Bolitogyrus falini* and except for the color of the genital segment, cannot be distinguished from it without the dissection of male specimens. The shape of the paramere differs between the two species but is subject to considerable variation. In general, the sides of the apical portion are subparallel or even slightly divergent, and the apex is rather blunt in *Bolitogyrus divisus*. In *Bolitogyrus falini*, the sides are convergent and the apex is narrower. The study of the lateral lobes of the aedeagus at unconventional angles, at high magnification and resolution using rSEM animations was critical in the development of the species concept used herein. rSEM animations revealed differences in the subapical teeth and the apices of the lateral lobe that perfectly coincided with the groups created using the color of the genital segment (see descriptions of *Bolitogyrus divisus* and *Bolitogyrus falini*). All other variation observed in the aedeagi was overlapping between species when most specimens had been dissected. Although the shape of the subapical tooth and of the lobe apex are difficult to observe at lower magnifications, the color of the genital segment should allow the identification of nearly all specimens without dissection.

##### Bolitogyrus falini

Taxon classificationAnimaliaColeopteraStaphylinidae

Brunke sp. n.

http://zoobank.org/420068FB-254B-4D4B-BC3F-A7B5EC1596CE

Figs 14D , 20H–L , 32B , 31B (map)

###### Type locality.

COSTA RICA, *Puntarenas*, Las Alturas Biological Station.

###### Type material.

**Holotype** ♂ (SEMC): COSTA RICA, Puntarenas Prov., Las Alturas Biol. Sta., 1600 m, 08°56.17'N, 82°50.01'W, 31.V.2004, J.S. Ashe, Z. Falin, I. Hinojosa, Ex. fungus covered logs, CR1AFH04 066 [white printed label] / SM0607578 [white barcode label] / Holotype, *Bolitogyrus falini* Brunke, sp. n. [red printed label].

**Paratypes** (31 ♂ 12 ♀, INBIO, PTC, SEMC, ZMUC): same as holotype, SM0607515, 1 ♂ (SEMC). **COSTA RICA:** *Puntarenas*, Altimira biological station, 9°01.76'N, 83°00.49'W, 1510-1600 m, ex. fungus covered logs, 5.VI.2004, CR1AFH04 109, SM0607622, SM0607623, SM0607624, 2 ♂ 1 ♀ (SEMC), same except: 6.VI.2004, ex. pyrethrum fogging hollow tree, CR1AFH04 133, SM0607659, 1 ♂ (SEMC), same except: 4 to 7.VI.2004, flight intercept trap, CR1AFH04 144, SM0606693, SM0606698, 2 ♂ (SEMC); Alturas de Coton biological station, 1490 m, ex. fog logs, 27.III.2000, CR-1099, P.N. Thomas, 1 ♂ (PTC); Coto Brus, Est. Pittier, 1500 m, 5 to 6.IX.2000, R. González Luz, L_S_330030_578645 #63373, INB0003336210, 1 ♂ (INBIO); Durika Biological Reserve, 9°15.966 N, 83°14.201 W, 1460 m, premontane moist forest, creek valley site, FIT, 15-18.VI.2012, leg. Solodovnikov, Brunke, Puliafico & Selvantharan, 1 ♂ (ZMUC), same except: fogging fungusy logs, 15.VI.2012, 1 ♂ (ZMUC); Hacienda La Amistad, 8°56.395'N, 82°47.465'W, 1500 m, premontane moist forest, fogging fungusy logs, 9.VI.2012, leg. Solodovnikov, Brunke Puliafico & Selvantharan, 3 ♂ 2 ♀ (ZMUC), same except: FIT, 9-13.VI.2012, 1 ♀ (ZMUC); La Amistad National Park, Est. Las Mellizas, Finca Cafrosa, 1300 m, III.1990, M. Ramirez & G. Mora, 316100-596100, CR1000162659, CR1000162719, CR1000163702, 1 ♂ 2 ♀ (INBIO, ZMUC); Las Cruces biological station, 8°47.17'N, 82°50.01'W, 1330 m, ex. fungus covered logs, 29.V.2004, J.S. Ashe, Z. Falin, I. Hinojosa, CR1AFH04 023, SM0607636, SM0607637, 1 ♂ 1 ♀ (SEMC); Las Alturas biological station, 8°56.17'N, 82°50.01'W, 1660 m, ex. pyrethrum fogging tree covered with fleshy white polypores, 31.V.2004, J.S. Ashe, Z. Falin, I. Hinojosa, CR1AFH04 065, SM0607632, 1 ♂ (SEMC), same locality except: ex. fungus covered logs, 1.VI.2004, CR1AFH04 069, SM0607565, SM0607566, SM0607567, SM0607568, 3 ♂ 1 ♀ (SEMC), same locality except: fungus covered logs, 31.V.2004, CR1AFH04 067, SM0607579, 1 ♀ (SEMC), same locality except: fungus covered logs, 2.VI.2004, CR1AFH04 084, SM0607514, 1 ♀ (SEMC), same locality except: fungus covered logs, 3.VI.2004, CR1AFH04 093, SM0607516, SM0607517, 1 ♂ 1 ♀ (SEMC). **PANAMA:** *Chiriquí*, 27.7 km W Volcan, Hartmann’s Finca, 8°51'48"N, 82°44'36"W, 1450 m, ex. fungusy log, 17.VI.1996, J. Ashe, R. Brooks, PAN1AB96 168, SM0050727, SM0050720, SM0039648, 3 ♂ 1 ♀ (SEMC), same locality except: 8°45'N, 82°48'W, 1450 m, fogging fungusy log, 14.VI.1995, #213, SM0005196, 1 ♂ (SEMC), same except: 15.VI.1995, #223, SM0021106, SM0021117, 2 ♂ (SEMC), 16.VI.1995, #226, SM0000554, SM0000849, SM0000890, SM0005582, SM0021210, 5 ♂ (SEMC).

###### Diagnosis.

Within the Bullatus Lineage: pronotum with three punctures in the dorsal row (Fig. 7D); lateral margins of pronotum only weakly convergent anteriad, sides nearly straight in anterior half (Fig. 7D); genital segment of both sexes dark, not contrasting with previous segments (Fig. 14D).

###### Description.

Measurements ♂ (n=5): HW/HL 1.62–1.71; PW/PL 1.44–1.60; EW/EL 1.21–1.29; ESut/PL 0.81–0.92; PW/HW 1.03–1.06; forebody length 3.8–4.2 mm.

Measurements ♀ (n=5): HW/HL 1.57–1.65; PW/PL 1.52–1.56; EW/EL 1.19–1.32; ESut/PL 0.85–0.92; PW/HW 1.03–1.06; forebody length 4.1–4.3 mm.

Similar to *Bolitogyrus divisus* and differing only in the following: pale areas of pronotum usually darker, orange-brown; tergite VIII always at least partially darkened apically or laterally; genital segment of both sexes dark, dark brown (Fig. 14D); lateral lobes of aedeagus with subapical tooth on dorsal face, tooth flattened, apex of lateral lobe flanged, not evenly cylindrical (Fig. 32B); some specimens with less-dilated paramere and with fields of peg setae less expanded basally and apically; paramere generally with sides of apical portion convergent and apex narrower.

###### Distribution.

Figure 31B. This species is only known to occur along the eastern portion of the Talamanca Cordillera in both Costa Rica and Panama.

###### Bionomics.

*Bolitogyrus falini* has been collected in March, May and June, and November, at elevations ranging from 1300-1660 m, from fungusy wood and using an FIT.

###### Etymology.

This species is dedicated to our colleague Zack Falin (SEMC), who collected many specimens of *Bolitogyrus* during extensive fieldwork in Central America.

###### Comments.

*Bolitogyrus falini* is sympatric with and similar to *Bolitogyrus bullatus* and *Bolitogyrus pseudotortifolius* but can be distinguished by the pronotum with three punctures in the dorsal row. *Bolitogyrus falini* and *Bolitogyrus divisus* appear to be sister species and are widely allopatric in Costa Rica. For details on their separation, see under *Bolitogyrus divisus*.

##### Bolitogyrus inexspectatus

Taxon classificationAnimaliaColeopteraStaphylinidae

Brunke sp. n.

http://zoobank.org/9D04643D-278A-41C5-9A29-224323FB1153

Figs 10D , 19K–M , 24D , 31B (map)

###### Type locality.

Costa Rica, San Jose, Jigueral.

###### Type material.

**Holotype** ♂ (PTC): San Jose, Jigueral, 850 mts., Finca J. Chavez [white printed label] / ex. pore mushrooms on log, ‘25/Feb/1998’, CR#803, P.N. Thomas [white printed label] / ILLUSTRATED [pink printed label] / Holotype, *Bolitogyrus inexspectatus* Brunke, sp. n. [red printed label].

###### Diagnosis.

Within the Bullatus Lineage: pronotum with two punctures in the dorsal row (Fig. 7C); dark abdominal segment VIII (except for narrowly pale base); humerus with pale marking wider than half the distance between scutellum and humeral angle (Fig. 10D).

###### Description.

Measurements ♂ (n=1): HW/HL 1.67; PW/PL 1.50; EW/EL 1.21; ESut/PL 0.85; forebody length 4.0 mm.

Similar to *Bolitogyrus divisus* and differing only the following: head with faint metallic bronze reflection; pronotal protuberance not paler than rest of disc; elytra with apical margin more distinctly pale, humeral area with broad, semi-circular pale spot reaching beyond half the distance from humerus to scutellum, epipleuron with small, pale spot about midlength; abdominal paratergites paler at base and apex, abdominal sternites with apicolateral pale spots; abdominal segments not distinctly darker than one another; tergite VIII dark with narrowly pale base; genital segment dark; pro- and mesofemur entirely pale, metafemur with small, subapical dark band; head with frontal impression not obscured by sculpture, frons relatively smooth, sculpture weakly formed; posterior protuberances of head entirely impunctate or with one puncture; pronotum without micropunctures on disc; pronotal protuberance weakly developed in lateral view; with two punctures in dorsal row of pronotum; scutellum with several coarse, contiguous and shallow, asetose punctures; lateral lobes of aedeagus with subapical tooth projecting at right angle to dorsoventral plane, tooth completely flattened and rounded at apex (Fig. 19K–L); lateral lobes in parameral view broadly separated at base by a U-shaped emargination, becoming narrowly separated and parallel in apical half, together forming an arrowhead shape (Fig. 19K); paramere in lateral view flexed ventrad and contacting median lobe; paramere bifurcate, in parameral view, lobes separated by very broadly rounded emargination (Fig. 19K); paramere with only one, large peg seta on one lobe, other lobe with peg setae (Fig. 19L). Female unknown.

###### Distribution.

Figure 31B. Known only from the type locality in Costa Rica.

###### Bionomics.

The holotype was collected in February, at a relatively low elevation from a log overhanging a creek (‘Rio Chavez’) running through a ravine (P.N. Thomas *pers. comm.*).

###### Etymology.

*Bolitogyrus inexspectatus* externally resembles *Bolitogyrus tortifolius* and *Bolitogyrus pseudotortifolius* and it was initially thought that these species could be closely related. The species epithet refers to the ‘unexpected’ discovery of the distinct aedeagal characters of the Divisus Group in this species.

###### Comments.

Of the species sympatric with *Bolitogyrus inexspectatus*, *Bolitogyrus bullatus* is the only similar species. It can be readily distinguished from it by the pronotum with two punctures in the dorsal row.

#### Strigifrons Group

**Figure 9.**
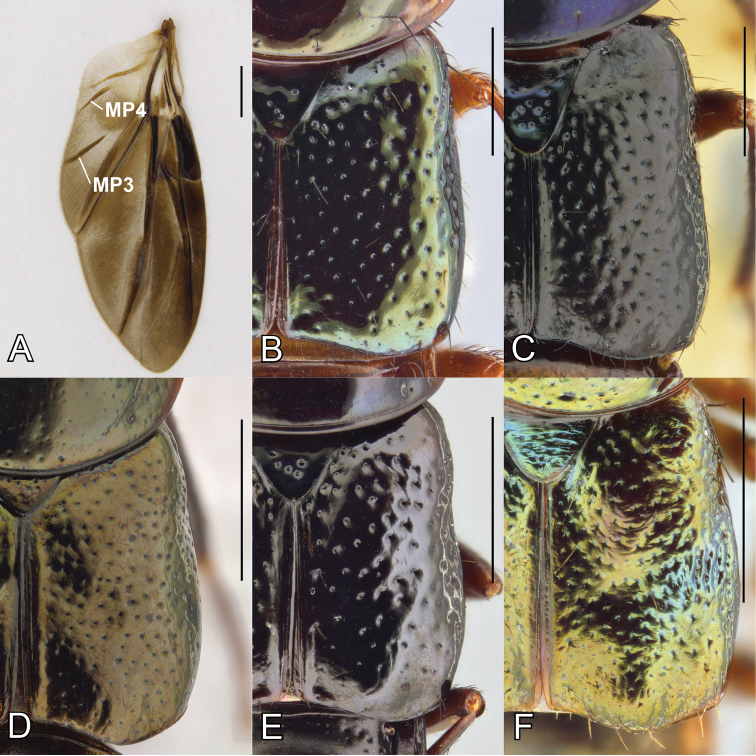
Hindwing: *Bolitogyrus bechyneorum* (Scheerpeltz) (**A**). Elytra, dorsal: *Bolitogyrus fulgidus* (Sharp) (**B**); *Bolitogyrus bechyneorum* (**C**); *Bolitogyrus marquezi* Brunke (**D**); *Bolitogyrus newtoni* Brunke (**E**); *Bolitogyrus viridescens* (**F**). Scale bars = 1 mm.

**Figure 10.**
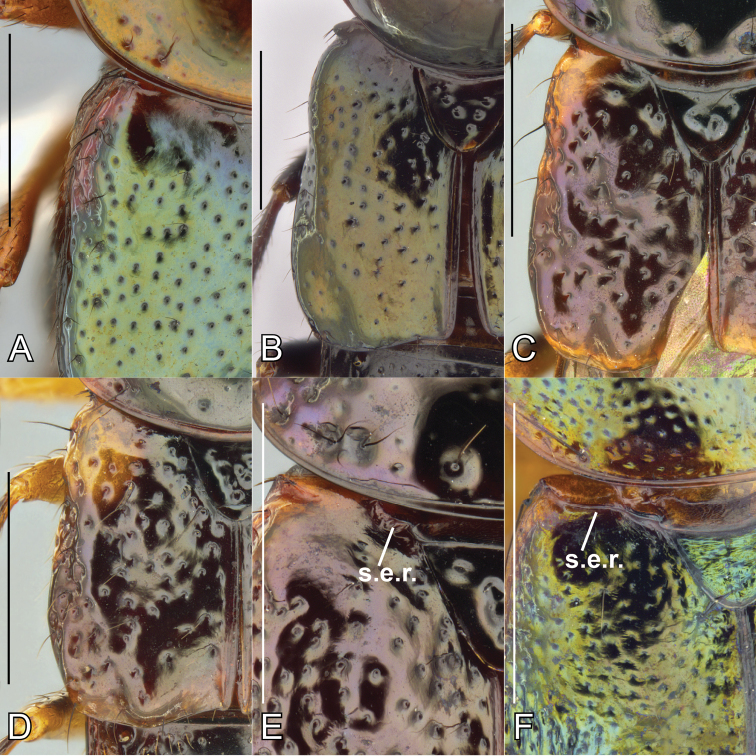
Elytra, humeral area: *Bolitogyrus fulgidus* (Sharp) (**A**); *Bolitogyrus salvini* (Sharp) (**B**); *Bolitogyrus tortifolius* Brunke (**C**); *Bolitogyrus inexspectatus* Brunke (**D**); *Bolitogyrus bullatus* (Sharp) (**E**); *Bolitogyrus viridescens* Brunke (**F**). s.e.r – subbasal elytral ridge. Scale bars = 1 mm.

**Figure 11.**
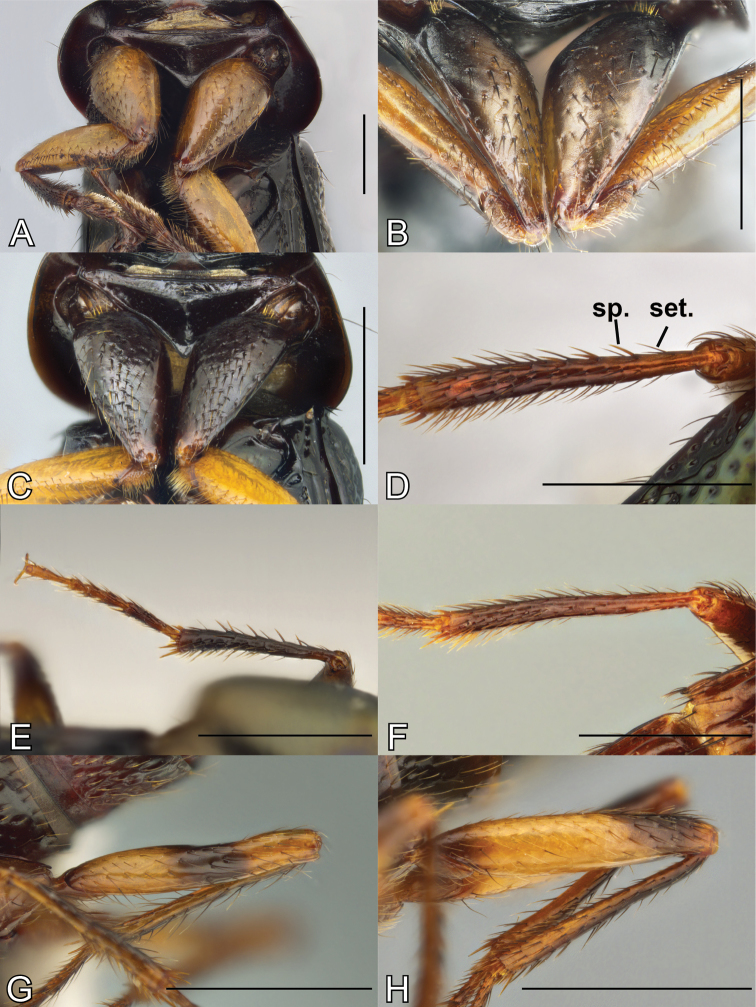
Ventral prothorax: *Bolitogyrus costaricensis* (Wendeler), male (**A**); *Bolitogyrus sallei* (Kraatz), male (close-up of procoxae) (**B**); *Bolitogyrus newtoni* Brunke, male (**C**). Mesotibia: *Bolitogyrus fulgidus* (Sharp) (**D**); *Bolitogyrus marquezi* Brunke (**E**). Metatibia: *Bolitogyrus fulgidus* (Sharp) (**F**). Metafemur: *Bolitogyrus bullatus* (Sharp) (**G**); *Bolitogyrus apicofasciatus* Brunke (**H**). sp. – spine, set. – seta. Scale bars = 1 mm.

This group is composed of three species (*Bolitogyrus strigifrons*, *Bolitogyrus silex* and *Bolitogyrus viridescens*), which share a strigulose patch on the elytra (Fig. 9F), dense yellow abdominal setae (Fig. 13E–F) and the basal elytral ridge reconnected to the humerus (Fig. 10F). The strigulose patch on the elytra and the dense, yellow abdominal setae are unique within *Bolitogyrus*. This group is currently known from Mexico and Guatemala.

##### Bolitogyrus strigifrons

Taxon classificationAnimaliaColeopteraStaphylinidae

(Wendeler, 1928)

Figs 6F , 13E , 17G–K , 23J , 31C (map)

Cyrtothorax strigifrons Wendeler, 1928: 34
Cyrtothorax strigifrons : Scheerpeltz 1974: 183

###### Type locality.

MEXICO: Veracruz, Jalapa [=Xalapa].

###### Type material.

**Holotype** ♂ (ZMHB): Jalapa, Mexico [printed label] / Cyrtothorax strigifrons n. sp., Wendeler det. [written name, rest printed] / Holotypus [red printed label] / strigifrons, Wdlr [large green label with written name] / Bolitogyrus strigifrons, Det. Chatzimanolis 2010 [written label] / Holotype ♂, *Cyrtothorax strigifrons* Wendeler 1927, det. A. Brunke 2013 [red printed label].

The single specimen located in the ZMHB is interpreted as the holotype as Wendeler (1928) stated that the ‘Type was in his collection’.

###### Other material.

**MEXICO:** *Hidalgo*: Zacualtipán, Camino a Sto. Domingo [trail to Santo Domingo], 20°38'00.7"N, 98°34'00.5"W, 1830 m, Bosque mixto? o mesofilo? [=mixed? or cloud forest?] pert. [=disturbed], en troncos podridos [in rotten logs], 16.VIII.2003, J. Asiain y J. Márquez, CC-UAEH ST-0194, 1 ♂ [tip of paramere missing] (UAEH).

###### Diagnosis.

Within the Bullatus Lineage: elytra with strigulose microsculpture (Fig. 9F); head with strigulose sculpture (Fig. 6F); posterior protuberances not creating expansive impunctate areas (Fig. 6F).

###### Redescription.

Measurements ♂ (n=2): HW/HL 1.38–1.60; PW/PL 1.48–1.59; EW/EL 1.22–1.30; ESut/PL 0.90–0.91; PW/HW 1.07–1.09; forebody length 3.5 mm.

Coloration: Body dark brown, head, pronotum and elytra with bronze metallic reflection; elytral epipleuron paler, brownish-orange; abdominal segments often with apical margin paler; maxillary and labial palpi pale to dark reddish brown; antennomeres I–V reddish-orange, with some apical darkening, VI–X dark brown, contrasting with previous, apical segment distinctly paler, yellow to orange; legs bicolored: forecoxa yellow, meso- and metacoxa dark yellow-brown, femur yellow with dark brown bands at base and apex, tibia brown to dark brown, with or without medial face lighter, tarsus brown.

Head with median frontal impression present but weakly delimited; with central protuberance, smooth and glossy; base of head with small paired protuberances not creating expansive impunctate areas; dorsal surface of head densely, coarsely punctate, punctures longitudinally compressed to form long, smooth ridges; dorsally, microsculpture absent except as broken lines on frons and as well-developed lines on temples.

Pronotum with scattered micropunctures, with one puncture in dorsal row (i.e. only marginal puncture), lateral portion with deep, strigulose and coarse micropunctures; with protuberance distinct in lateral view; scutellum with several transversely rugose punctures; subbasal ridge horizontal and connected to humerus; elytra weakly transverse, suture barely shorter than pronotum at midline; surface without microsculpture, strongly uneven; surface with coarse, dense and asetose punctation, becoming strigulose over nearly entire disc and epipleuron (holotype) or mostly mediolaterally; macrosetae on disc of elytra short, erect, not distinct from overall surface sculpture in lateral view

Abdominal tergites IV–VIII with moderately dense and long, gold pubescence basally and laterally, III–V impressed at base; disc of tergites III–VI or III–VII impunctate medially, this impunctate area becoming smaller on successive tergites; sternites III–V with basal transverse line bluntly projected posteriad at middle, punctures becoming denser, larger and more deeply impressed toward base of segment; abdominal sternites with coarse microsculpture, interspaces wider than lines.

Median lobe in lateral view curved ventrad; in parameral view, median lobe in apical two-thirds narrowed to blunt apex (Fig. 17G); paramere not divided apically but with median suture, shape as in Fig. 17G; peg setae in a single row along each lateral edge of parameral apex (Fig. 17K); internal sac with small, central, tear-drop shaped sclerite (Fig. 17H–I). Male sternite VIII with transverse basal line broken at middle, with distinct U-shaped emargination, impressed and glabrous in small triangular area near emargination; male sternite IX sparsely setose, distinctly asymmetrical at base and moderately emarginate at apex (Fig. 23J).

Female unknown.

###### Distribution.

Figure 31C. Known only from Hidalgo and Veracruz states in Mexico.

###### Bionomics.

One specimen was found in a rotten log during August at 1830 m.

###### Comments.

Among potentially sympatric species, *Bolitogyrus strigifrons* is similar to *Bolitogyrus viridescens* but is easily distinguished by the strigulose sculpture on the head. The non-type male was received pre-dissected but, unfortunately, the tip of the paramere was missing. Despite this, I am reasonably confident that this specimen belongs to *Bolitogyrus strigifrons* based on the sculpture of the head and the general shape of the median lobe.

##### Bolitogyrus silex

Taxon classificationAnimaliaColeopteraStaphylinidae

Brunke sp. n.

http://zoobank.org/197A20D8-BF58-4AE1-A92B-49CCEE6941A0

Figs 6A , 17L–O , 23K , 26H , 31C (map)

###### Type locality.

GUATEMALA, Zacapa, 3.5 km SE La Union.

###### Type material.

**Holotype** ♂ (SEMC): Guatemala, Zacapa, 3.5 km SE La Union, 1500 m, 23–25 June 1993, J. Ashe & R. Brooks, #102, ex: flight intercept trap [white printed label] / SM0038000 [white barcode label] / Holotype, *Bolitogyrus silex* Brunke, sp. n. [red printed label].

**Paratypes** (2♂, 3 ♀, SEMC, UTCI, ZMUC): **GUATEMALA:** *Zacapa*, 3.5 km SE La Union, 1500 m, 25-VI.1003, J. Ashe and R. Brooks, #115, ex. crustose mushrooms, SM0037985, 1 ♂ (SEMC), same except: 1600 m, R. Anderson, #93-20, ex. cloud forest, SM0037988 1 ♀ (SEMC); 2 km SE La Union, 14.94661, -89.27612 ± 21 m, 1555m, 12 to 15.V.2009, Malaise trap, secondary cloud forest, LLAMA#Ma-B-03-1-02, DNA Voucher (S. Chatzimanolis), extraction SC-180, extracted i.23.2010, 1 ♀ (UTCI), same except: 14.34707, -89.27606 ± 7 m, 1560 m, secondary cloud forest, Malaise trap, LLAMA Ma-B-03-1-01, 1 ♂ (SEMC), same except: 14.95390, -89.27638 ± 200 m, 1430 m, cloud forest, sifted leaf litter, LLAMA Wa-B-03-2-all, 1 ♀ (ZMUC, DNA extracted).

###### Diagnosis.

Within Bullatus Lineage: elytra with strigulose patch (Fig. 9F); posterior protuberances large and glossy, creating expansive impunctate areas (Fig. 6A).

###### Description.

Measurements ♂ (n=2): HW/HL 1.39–1.50; PW/PL 1.30–1.36; EW/EL 1.08–1.12; ESut/PL 0.85–0.96; PW/HW 1.06; forebody length 4.2 mm.

Measurements ♀ (n=2): HW/HL 1.36–1.42; PW/PL 1.25–1.28; EW/EL 1.17–1.19; ESut/PL 0.88–0.89; PW/HW 1.03–1.07; forebody length 4.0–4.4 mm.

Similar to *Bolitogyrus strigifrons* and differing only in the following: forebody distinctly longer; body dark brown, head and elytra with bronze to bluish-green metallic reflection due to sculpture, pronotum with bronze metallic reflection; abdomen with apical margins paler, sometimes with pale stripe on lateral border of sternites; antennomeres I–V reddish-orange, VI–XI gradually darkening to dark brown apex; legs bicolored: procoxa entirely yellowish (male) or with basal and apical darkening (female), profemur yellow with basal (male) or basal and apical darkening (female), tibia brown to dark brown with medial face lighter, meso- and metacoxa yellow with dark areas (male) or dark brownish yellow (female); median frontal impression indistinct, in most specimens, obliterated by punctures; base of head with posterior protuberances creating expansive impunctate areas; dorsal surface of head with dense, coarse and often rugose to strigulose punctures, punctures rarely forming short longitudinal ridges; pronotum slightly less transverse; lateral areas of pronotum with deep, coarse, often confluent and rugose micropunctures; pronotum slightly less transverse; elytra slightly less transverse, strigulose sculpture limited to small area mediolaterally; impunctate areas on abdominal tergites III–VI subequal; median lobe cylindrical, in lateral view slightly sinuate, narrowed to a short, broad and triangular apical portion, further narrowed to a thin, ventrally produced apex (Fig. 17N); in parameral view, median lobe narrowed at midlength and then dilated to subapex, apical portion short, broad, apex obtuse (Fig. 17L); shape of paramere as in Fig. 17L; peg setae along lateral edges of paramere in apical half, a pair of clusters at apex (Fig. 17O); internal sac with a pair of large, heavily sclerotized, talon-shaped sclerites (Fig. 17M); male sternite VIII with emargination slightly shallower but still distinct; sternite IX with base only weakly asymmetrical, apical emargination slightly deeper (Fig. 23K); female tergite X broad, apex obtuse and with shallow, narrow emargination (Fig. 26H); basal margin of laterotergal sclerites fused and thickened across base of tergite X, female laterotergal sclerites expanded and overlapping with tergite X (Fig. 26H).

###### Distribution.

Figure 31C. Known only from Zacapa state in Guatemala.

###### Bionomics.

Specimens were collected in cloud forests at elevations ranging from 1430–1600 m, in flight traps, by sifting litter and on ‘crustose mushrooms’, during May and June.

###### Etymology.

The species epithet is a Latin noun meaning ‘stone’ or ‘flint’ and refers to the pair of heavily sclerotized sclerites of the internal sac that appear weighty, like stones. It is to be used as a noun in apposition.

###### Comments.

Among sympatric species, *Bolitogyrus silex* not easily confused with any other species and is easily recognized by the strigulose patch on the elytra.

##### Bolitogyrus viridescens

Taxon classificationAnimaliaColeopteraStaphylinidae

Brunke sp. n.

http://zoobank.org/438E36F1-20E8-4350-A8F4-E5202D9277D4

Figs 2D , 6G , 9F , 10F , 13F , 18A–E , 23L , 26I , 31C (map)

###### Type locality.

MEXICO, Puebla, 9.66 km east of Teziutlán.

###### Type material.

**Holotype** ♂ (CNC): 6mi. East, Tezuitlan, Pueb. MEX., VIII 4-6 [19]60, H.F. Howden [white printed label] / Holotype, *Bolitogyrus viridescens* Brunke, sp. n. [red printed label].

**Paratypes** (2 ♂, 1 ♀, CNC): MEXICO: same data as holotype.

###### Diagnosis.

Within Bullatus Lineage: elytra with strigulose patch (Fig. 9F); head without distinct posterior protuberances, without large glossy, impunctate areas (Fig. 6G); head without strigulose sculpture (Fig. 6G).

###### Description.

Measurements ♂ (n=3): HW/HL 1.38–1.48; PW/PL 1.35–1.43; EW/EL 1.03–1.17; ESut/PL 1.00; PW/HW 1.00; forebody length 3.6–3.7 mm.

Measurements ♀ (n=1): HW/HL 1.38; PW/PL 1.42; EW/EL 1.09; ESut/PL 0.96; PW/HW 1.03; forebody length 4.1 mm.

Similar to *Bolitogyrus strigifrons* and differing only in the following: head, lateral part of pronotum, scutellum and patches of strigulose sculpture on elytra with brilliant greenish-blue metallic reflection, remaining areas on pronotum and elytra with bronze metallic reflections; maxillary and labial palpi yellow; antennomeres I–V yellow-orange, VI–XI gradually darkening to brown apex; forecoxa yellow, meso- and metacoxa yellow (male) or yellow and dark yellow-brown (female), femur yellow with dark brown band near apex (male) or base and apex (female), tibia brown to dark brown, with or without medial face lighter, tarsus brown; forebody longer; median frontal impression indistinct, in most specimens, obliterated by punctures; base of head without distinct posterior protuberances; dorsal surface of head densely, coarsely and rugosely punctate, punctures not becoming strigulose; pronotum slightly less transverse; entire surface with many micropunctures, lateral portion with deep, rugose and asetose punctures; pronotal protuberance indistinct in lateral view; elytra slightly less transverse; elytral suture more or less as long as pronotum at middle; elytra with punctation becoming strigulose only lateromedially; tergites III–V with impunctate areas medioapically, approximately equal in size; abdominal sternites with dense microsculpture, interspaces about as wide as lines; median lobe in lateral view broad, narrowed at midlength, widened in apical third and abruptly narrowed to minutely produced, acute apex (Fig. 18D); in parameral view, median lobe subparallel, near apex, narrowed to obtuse, blunt apex (Fig. 18A); paramere not divided apically but with median suture, shape as in Fig. 18A; peg setae densely distributed on entire apical portion of paramere (Fig. 18E); internal sac with well-sclerotized, horseshoe-shaped sclerite with a broadly expanded base (Fig. 18B–C); male sternite VIII with transverse basal line broken at middle, with broadly, shallowly emarginate apex, impressed and glabrous in small triangular area near emargination; male sternite IX sparsely setose, moderately asymmetrical at base and emarginate at apex (Fig. 23L); female tergite X elongate, with broadly rounded to nearly truncate apex (Fig. 26I); basal margin of laterotergal sclerites fused and thickened across base of tergite X, female laterotergal sclerites greatly expanded and overlapping with tergite X (Fig. 26I).

###### Distribution.

Figure 31C. *Bolitogyrus viridescens* is known only from Teziutlán in Puebla, Mexico.

###### Bionomics.

The type series was collected in early August.

###### Etymology.

The species epithet refers to the brilliant green metallic reflection of the forebody.

###### Comments.

Among potentially sympatric species, *Bolitogyrus viridescens* is similar to *Bolitogyrus strigifrons* but is easily distinguished by the non-strigulose sculpture on the head.

#### Species *incertae sedis* in *Bolitogyrus*

##### Bolitogyrus bechyneorum

Taxon classificationAnimaliaColeopteraStaphylinidae

(Scheerpeltz, 1974)

Figs 2E , 9A, C , 13B , 16J–L , 23G , 25G , 29D (map)

Cyrtothorax bechyneorum Scheerpeltz, 1974: 189.

###### Type locality.

El Salvador, La Paz, in vicinity of Volcan San Vicente.

###### Type material.

*Cyrtothorax bechyneorum* Scheerpeltz, 1974. **Holotype** (♀, NMW): ‘♂’ [male symbol on small card] / v. San Vicente, Finca La Paz, 5. 8. 1959 [hand printed label] / Rep. San Salvador, leg. Dr. J. Bechyné [typed label] / Cyrtothorax bechyneorum Scheerp. [hand printed label] / Holotypus [red, hand printed label] / TYPUS, Cyrtothorax, Bechyneorum, O. Scheerpeltz [red label, printed and typed] / Bechyneorum, Scheerp. [green, hand printed label] / ex. coll., Scheerpeltz [blue, printed label].

Although stated to be a male by Scheerpeltz (1974), the holotype is actually a female. The Scheerpeltz collection (NMW) contains only female specimens but males were available from other localities. The holotype was easily associated with the males and females collected in Mexico based on the distinctive habitus of this species, elytral morphology and the general shape of female tergite 10.

###### Other material.

**MEXICO:** *Chiapas*: 7.0 km W Rizo de Oro, 460m, ex. leaf litter, 26.VI.1979, J.S. Ashe, SM0038001, 1 ♀ (SEMC). *Jalisco*: Casimiro Castillo, El Parotal, cam. El Parotal, BTC, 700m, 1 to 2.VII.1995, J.L. Navarrete, 1 ♀ (CZUJ); San Sebastián del Oeste, camino hacia [=road to] La Bufa, BMM, 20°45'19.8"N, 104°50'15.6"W, 1535m, ex. tronco [=log], 28.VI.2003, J. Cortès-Aguilar, 1 ♂ (CZUJ); Tequila, Volcán de Tequila, BMM, 2350m, ex. hojarasca [=litter], 30.IV.1994, H. Fierros, 1 ♀ (CZUJ). *Morelos*: Sta. Catarina, SBC y CT, ex tronco caído [=fallen tree], 25.VI.1996, J. Márquez, 1 ♂ (ZMUC). *Oaxaca*: 5 km NE of Comala [Comila], 334m, selva mediana [=tropical forest], en troncos con hongos [=in logs with mushrooms], 10.VII.2002, J. Asiain y Márquez, 1 ♂ (UAEH). **EL SALVADOR:** *Ahuachapan*: Apaneca, 14.VII.1959, Dr. J. Bechyné, 1 ♀ (NMW). *La Paz*: Vulcan San Vicente, 3.VIII.1959, 1 ♀ (NMW). **NICARAGUA:** *Esteli*: vicinity, Cerro ‘Estensuela’ [=Estenzuela], 1400 m, pine forest, II.1997, E. v. d. Berghe, 1 ♀ (CMNH).

###### Diagnosis.

Head without central protuberance; antenna bicolored (Fig. 5A); genital and abdominal segment VIII dark, not distinctly paler than previous segments; head and pronotum with blue to bluish-green metallic reflections in combination with dull silver metallic reflections on elytra (Fig. 2E); abdominal sternites with fine transverse microsculpture, interspaces about as wide as lines (Fig. 13D); disc of elytra without distinct protuberances, punctation relatively dense and regularly spaced (Fig. 9C).

###### Redescription.

Measurements ♂ (n=3): HW/HL 1.44–1.50; PW/PL 1.29–1.36; EW/EL 1.18–1.28; ESut/PL 0.69–0.74; PW/HW 1.04–1.14; forebody length 5.2–5.7 mm.

Measurements ♀ (n=5): HW/HL 1.39–1.45; PW/PL 1.25–1.31; EW/EL 1.19–1.24; ESut/PL 0.71–0.73; PW/HW 1.07–1.12; forebody length 5.3–5.5 mm.

Similar to *Bolitogyrus buphthalmus* and differing only in the following: head and pronotum with blue to bluish-green metallic reflections, elytra with dark silver metallic reflections; antenna bicolored but apical antennomere not distinctly paler than previous segments; legs bicolored: pro- and mesocoxa pale (males) or slightly darker than pro- and mesofemur (females); metacoxa darker than metafemur; pro- and mesofemur pale yellow (males) or diffusely tinged with dark brown (female), without distinct dark apical band; apex of hind femur with crisp dark band (males) or diffuse darkening (female); tibia dark brown; punctures on head of variable diameter and spacing but distinctly denser and more deeply impressed than in *Bolitogyrus buphthalmus*; pronotum more convex in both sexes, with pair of depressions but without distinct protuberance in lateral view; disc of elytra without distinct protuberances, causing punctation to appear more evenly distributed; sternites III–V with basal transverse line sharply projected posteriad at middle, one female specimen with only weak projections; median lobe flattened and convex, with acute apex, in lateral view sinuate and projecting ventrad (Fig. 16K), in parameral view, not constricted at base (Fig. 16J); paramere deeply divided into two divergent lobes (Fig. 16K), peg setae developed as in Fig. 16L; male sternites VI–VIII with medioapical asetose, flattened, triangular areas, increasing in size from VI to VIII (Fig. 13B); male sternite IX with emargination shallower and wider than in *Bolitogyrus buphthalmus*, base more strongly asymmetrical (Fig. 23G); female tergite X elongate, raised discal area contiguous with base and not strongly converging apicad, apex evenly rounded, not produced (Fig. 25G).

###### Distribution.

Figure 29D. Known from northern Jalisco state in Mexico to Matagalpa province in Nicaragua.

###### Bionomics.

*Bolitogyrus bechyneorum* has been collected at a wide range of elevations (334-2350m) from leaf litter, dead wood and within a log with mushrooms. Most specimens were collected during June to August but one specimen each was taken in February and April.

###### Comments.

Among sympatric species, *Bolitogyrus bechyneorum* is most similar to *Bolitogyrus marquezi* but can be easily distinguished by the metallic blue forebody and the coarse punctation of the elytra. The holotype of *Bolitogyrus bechyneorum* was collected from ‘Finca La Paz’ near the double-coned volcano San Vicente, which has one cone in San Vicente state and one in La Paz. ‘Finca La Paz’ could not be located but it is assumed that the holotype was collected somewhere near a coffee plantation on the western cone, which is located in La Paz state.

##### Bolitogyrus marquezi

Taxon classificationAnimaliaColeopteraStaphylinidae

Brunke sp. n.

http://zoobank.org/CC2124F1-DB35-44AB-AAF7-931633FA7B6B

Figs 2F , 5C , 9D , 11E , 12F , 17D–F , 23I , 26G , 31D (map)

###### Type locality.

MEXICO, Morelos, Derrame del Chichinautzin.

###### Type material.

**Holotype** ♂ (MZFC): MÉXICO: Morelos, Derrame del Chichinautzin (La Pera). Bosque de pino encino [=pine-oak forest]. 2450 m. En troncos caídos [=on fallen logs]. 22.III.2001. JML Col. [white printed label] / *Bolitogyrus* sp. 1, MZFC [white printed label] / Holotype, *Bolitogyrus marquezi* Brunke, sp. n. [red printed label].

**Paratypes** (1 ♂ 1 ♀, SEMC, ZMUC): same data as holotype, 1 ♀ (ZMUC). **MEXICO:** Guerrero, 71 km NE Atoyac de Alvarez, 1700 m, ex: treefall litter, 25 July 1992, J.S. Ashe, #118, SM0037999, 1 ♂ (SEMC).

###### Diagnosis.

**Figure 12.**
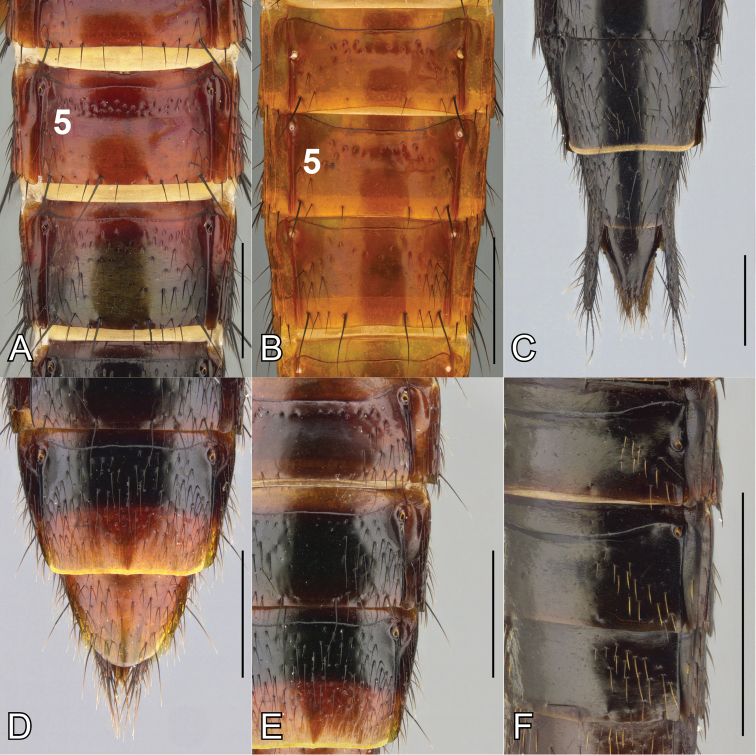
Abdomen, dorsal: *Bolitogyrus pictus* Smetana & Zheng (**A**); *Bolitogyrus ashei* Brunke (**B**); *Bolitogyrus costaricensis* (Wendeler) (**C**); *Bolitogyrus fulgidus* (Sharp) (**D–E**); *Bolitogyrus marquezi* Brunke (**F**). Scale bars = 1 mm.

Head without central protuberance; genital and abdominal segments VIII not distinctly paler than previous segments; elytra with small, poorly impressed punctures (Fig. 9D); abdominal tergites III–VII with lateral patches of short, appressed setae (Fig. 12F).

###### Description.

Measurements ♂ (n=2): HW/HL 1.36–1.40; PW/PL 1.27–1.28; EW/EL 1.16–1.24; ESut/PL 0.83–0.84; PW/HW 1.07–1.09; forebody length 3.9–4.4 mm.

Measurements ♀ (n=1): HW/HL 1.37; PW/PL 1.37; EW/EL 1.21; ESut/PL 0.90; PW/HW 1.11; forebody length 4.8 mm.

Similar to *Bolitogyrus buphthalmus* and differing only in the following: head, pronotum and elytra black with bronze metallic reflection; apical antennomere distinctly paler (Guerrero) or not (Morelos); legs nearly entirely yellowish, except for darkened apices of femora and tibiae, and entire to part of hind coxa (male), or legs nearly entirely dark brownish, except paler apices of femora and tibiae; head slightly less transverse than *Bolitogyrus buphthalmus*; punctures on head of variable diameter and spacing but distinctly denser than in *Bolitogyrus buphthalmus*, punctures on disc varying from strongly impressed (Morelos) to barely perceivable (Guerrero); median frontal impression distinct but shallow; entire surface of pronotum with scattered micropunctures; pronotum with pair of depressions but without distinct protuberance in lateral view; elytra with basal elytral ridge re-connected to humerus; elytra with punctures in much smaller depressions compared to those of *Bolitogyrus buphthalmus*, especially on disc near suture (Fig. 9D); sternites III–V with basal transverse line weakly and bluntly projected posteriad; abdominal sternites with coarse transverse microsculpture in apical half, interspaces wider than lines; microsculpture of dorsal abdomen with small connections between transverse lines (Morelos) or not (Guerrero); mesofemur with distinct spines on lateral face, not obscured by setae; median lobe cylindrical, in lateral view sinuate, with a short, broad and triangular apical portion (Fig. 17E); median lobe in parameral view subparallel and broadly rounded at apex (Fig. 17D); paramere weakly divided near apex or with only median seam, with a pair of short, longitudinal fields of peg setae laterally near apex (Fig. 17F); internal sac with large, horseshoe-shaped sclerite (Fig. 17D); male sternite VIII with transverse basal line distinctly interrupted at middle; male tergite IX with deeper, narrower emargination and base ending more bluntly (Fig. 23I); female tergite X broad, truncate apically, with base fused to accessory sclerite formed from expanded basal margin; female laterotergal sclerites expanded mediad to overlap with tergite X (Fig. 26G).

###### Distribution.

Figure 31D. The locality of one paratype could not be georeferenced due to vague label data (‘71 km NE Atoyac de Alvarez’). *Bolitogyrus marquezi* is known from Morelos and Guerrero states in Mexico.

###### Bionomics.

Specimens of this species were collected at 1700–2450 m, in March and July, in treefall litter and in an oak-pine forest on fallen logs.

###### Etymology.

The first author would like to dedicate this species to his colleague Dr. Juan Márquez Luna (MZFC), the collector of the holotype and female paratype, and someone who shares an interest in this genus.

###### Comments.

*Bolitogyrus marquezi* is most similar to and sympatric with *Bolitogyrus bechyneorum* but can easily be distinguished by the greenish-bronze forebody and finer punctation of the elytra. The holotype and paratype from Morelos state differ markedly from the male paratype from Guerrero in the abdominal microsculpture, punctation of the forebody and the coloration of the apical antennomere. However, the aedeagi of the males are very similar and differ only slightly in the shape of the parameral apex. Further study may reveal *Bolitogyrus marquezi* to be a complex of species but more male specimens are needed to readdress this.

##### Bolitogyrus newtoni

Taxon classificationAnimaliaColeopteraStaphylinidae

Brunke sp. n.

http://zoobank.org/027AF9EA-2A19-49D7-8766-C9A5FD6359C7

Figs 5D , 9E , 11C , 13C , 17A–C , 23H , 25H , 31D (map)

###### Type locality.

MEXICO, Chiapas, Jitotol.

###### Type material.

**Holotype** ♂ (FMNH): MEXICO: *Chiapas*: Jitotol (6 mi N, = 21 mi N Bochil), 5500 ft., 17°08'N, 92°53'W, 18-24.VIII.1971 [white printed label] / oak-pine-Liquidamber forest, dung trap (human), A. Newton, 358Dh, FMHD #71-563 [white printed label], FIELD MUS. NAT. HIST. / Bolitogyrus det. Newton 1994 [white printed and written label] / Holotype, *Bolitogyrus newtoni* Brunke sp. n. [red printed label].

**Paratypes** (1 ♂, 1 ♀, SEMC, FMNH): MEXICO: Oaxaca-Chiapas border, Montana Manantial, W of Rodulfo Figerosa [Rodolfo Figueroa], 1550 m, ex. yellow-brown spored mushroom clusters on log, 25.VI.1979, FMHD #79-294, J. S. Ashe, 1 ♀ (FMNH). GUATEMALA: *Suchitepéquez*: 4 k S Vol. Atitlán, 14.54915, -91.19055 ± 200 m, 1625 m, cloud forest, ex. sifted litter, 15.VI.2009, LLAMA09 W a-B-09-1-all, SEMC0885823, 1 ♂ (SEMC).

###### Diagnosis.

**Figure 13.**
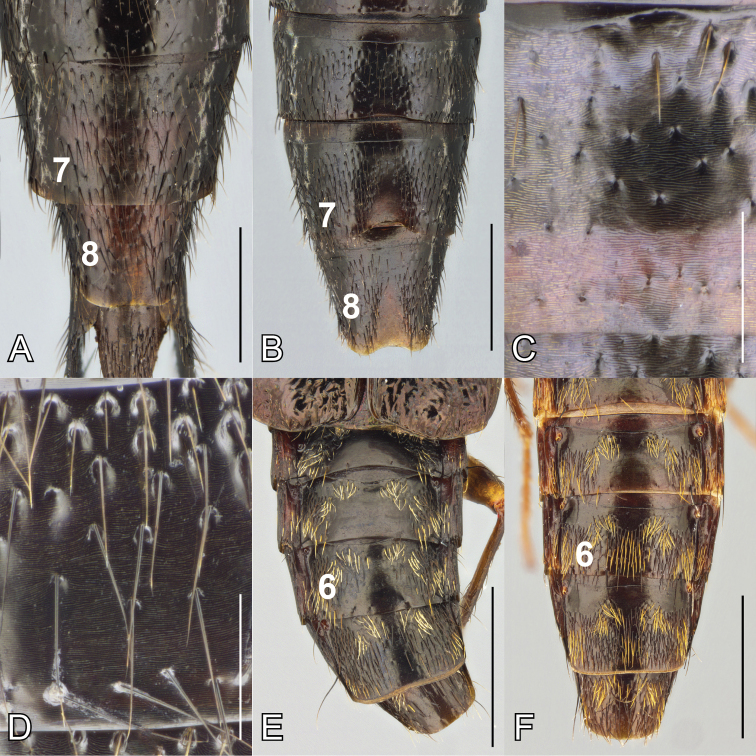
Abdomen, ventral: *Bolitogyrus costaricensis* (Wendeler) (**A**); *Bolitogyrus bechyneorum* (Scheerpeltz) (**B**). Abdomen, ventral, microsculpture: *Bolitogyrus newtoni* Brunke (**C**); *Bolitogyrus costaricensis* (**D**). Abdomen, dorsal: *Bolitogyrus strigifrons* (Wendeler) (**E**); *Bolitogyrus viridescens* Brunke (**F**). Scale bars = 1 mm (**A–B, E–F**), 0.5 mm (**C–D**).

**Figure 14.**
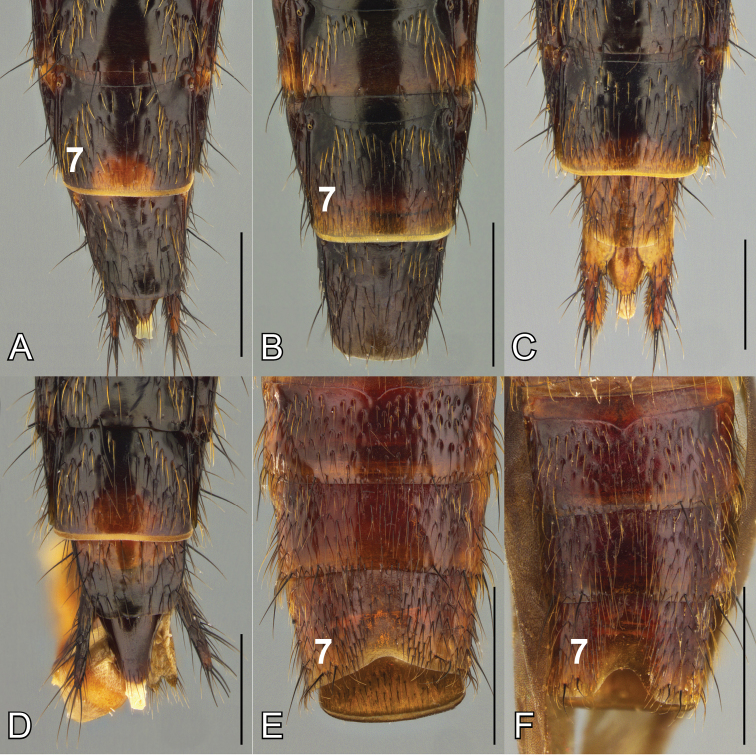
Abdomen, dorsal: *Bolitogyrus bullatus* (Sharp) (**A**); *Bolitogyrus apicofasciatus* Brunke (**B**); *Bolitogyrus divisus* Brunke (**C**); *Bolitogyrus falini* Brunke (**D**). Abdomen ventral: *Bolitogyrus bufo* Brunke (**E**); *Bolitogyrus thomasi* (**F**). Scale bars = 1 mm.

**Figure 15.**
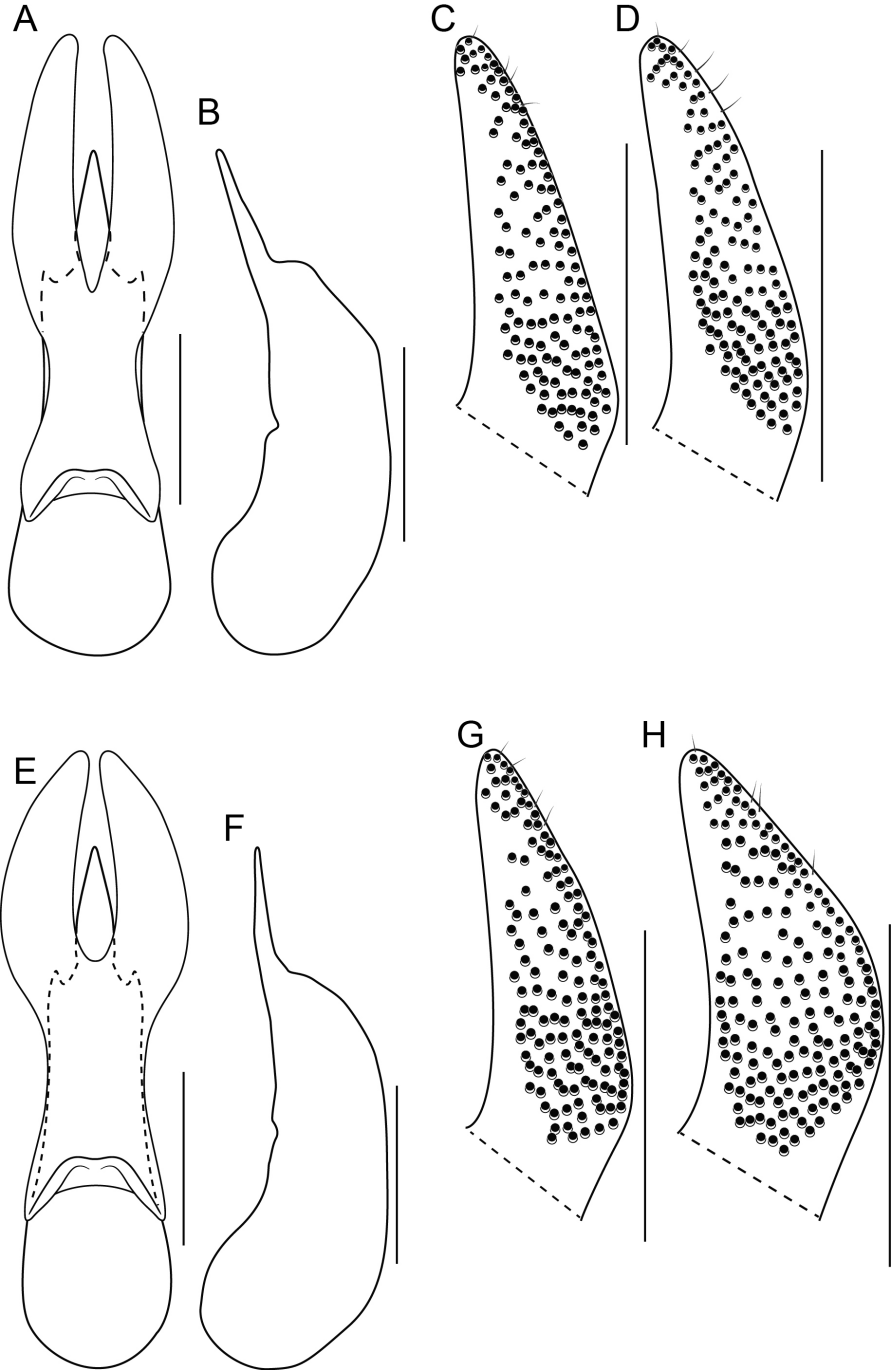
Aedeagus, parameral view (**A, E**). Aedeagus, lateral view (**B, F**). Underside of parameral lobe, with peg setae (**C, D, G, H**). *Bolitogyrus buphthalmus* (Erichson) (**A–D**); *Bolitogyrus costaricensis* (Wendeler) (**E–H**), (median lobe in lateral view similar in *Bolitogyrus prolixus*, *Bolitogyrus sallei* and *Bolitogyrus salvini*). Scale bar = 0.5 mm.

**Figure 16.**
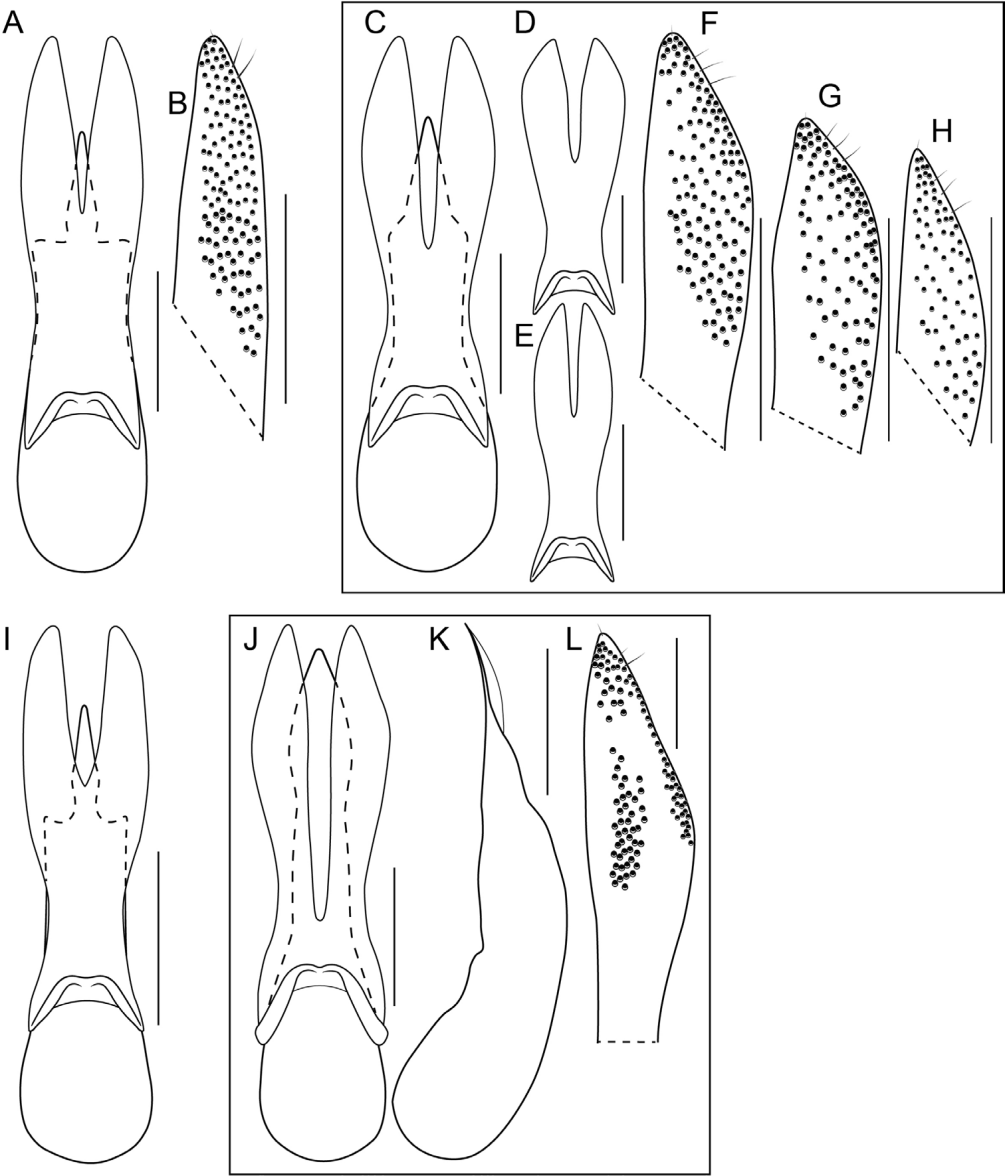
Aedeagus, parameral view (**A, C, I, J**). Aedeagus, lateral view (**K**). Paramere, parameral view (**D, E**). Underside of parameral lobe, with peg setae (**B, F, G, H, L**). *Bolitogyrus pulchrus* Brunke (**A, B**); *Bolitogyrus salvini* (Sharp) (**C–H**), morphotype I (**C, F**), morphotype III (**D, E, G, H**); *Bolitogyrus sallei* (Kraatz) (**I**); *Bolitogyrus bechyneorum* (Scheerpeltz) (**J–L**). Scale bar = 0.5 mm (**A–K**), 0.1 mm (**L**).

Head without central protuberance; genital and abdominal segments not distinctly paler than previous segments; apical half of abdominal sternites with relatively coarse transverse microsculpture, interspaces wider than lines (Fig. 13C); apical antennomere not paler than previous subapical segments (Fig. 5D); disc of elytra uneven, punctures irregularly spaced (Fig. 9E) abdominal tergites III–VII with typical lateral setae not short and appressed (c.f. Fig. 12E).

###### Description.

Measurements ♂ (n=2): HW/HL 1.41–1.45; PW/PL 1.36–1.40; EW/EL 1.15–1.16; ESut/PL 0.84; PW/HW 1.09–1.10; forebody length 4.0 mm.

Measurements ♀ (n=1): HW/HL 1.38; PW/PL 1.39; EW/EL 1.14; ESut/PL 0.86; PW/HW 1.08; forebody length 4.5 mm.

**Figure 17.**
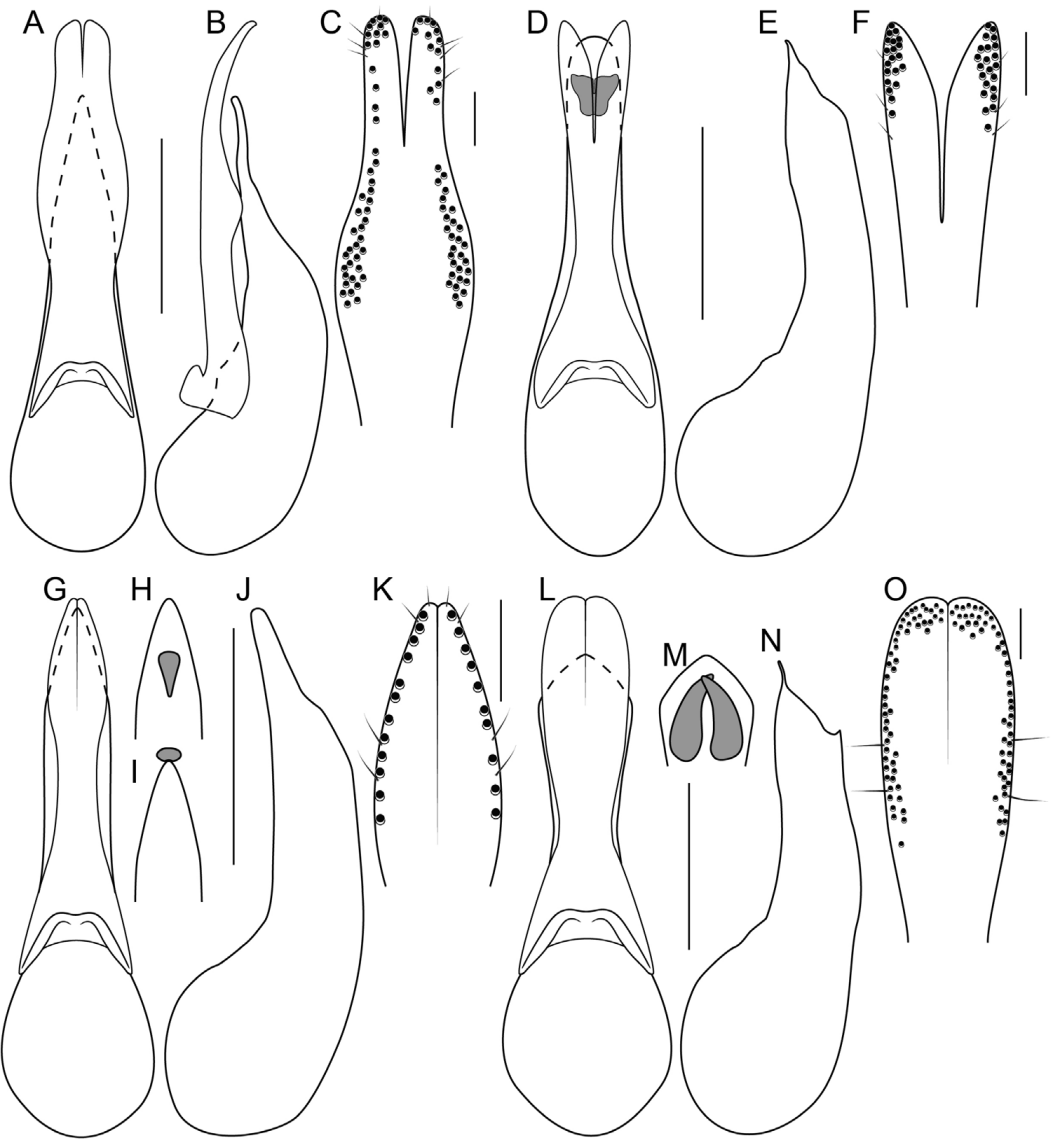
Aedeagus, parameral view (**A, D, G, L**), apex with internal sclerites (**H, I** (everted), **M**). Aedeagus, lateral view (**B, E, J, N**). Underside of parameral lobe, with peg setae (**C, F, K, O**). *Bolitogyrus newtoni* Brunke (**A–C**); *Bolitogyrus marquezi* Brunke (**D–F**); *Bolitogyrus strigifrons* (Wendeler) (**G–K**); *Bolitogyrus silex* Brunke (**L–O**). Scale bar = 0.5 mm (**A, C, D, E, G–J, L–N**), 0.1 mm (**C, F, K, O**).

**Figure 18.**
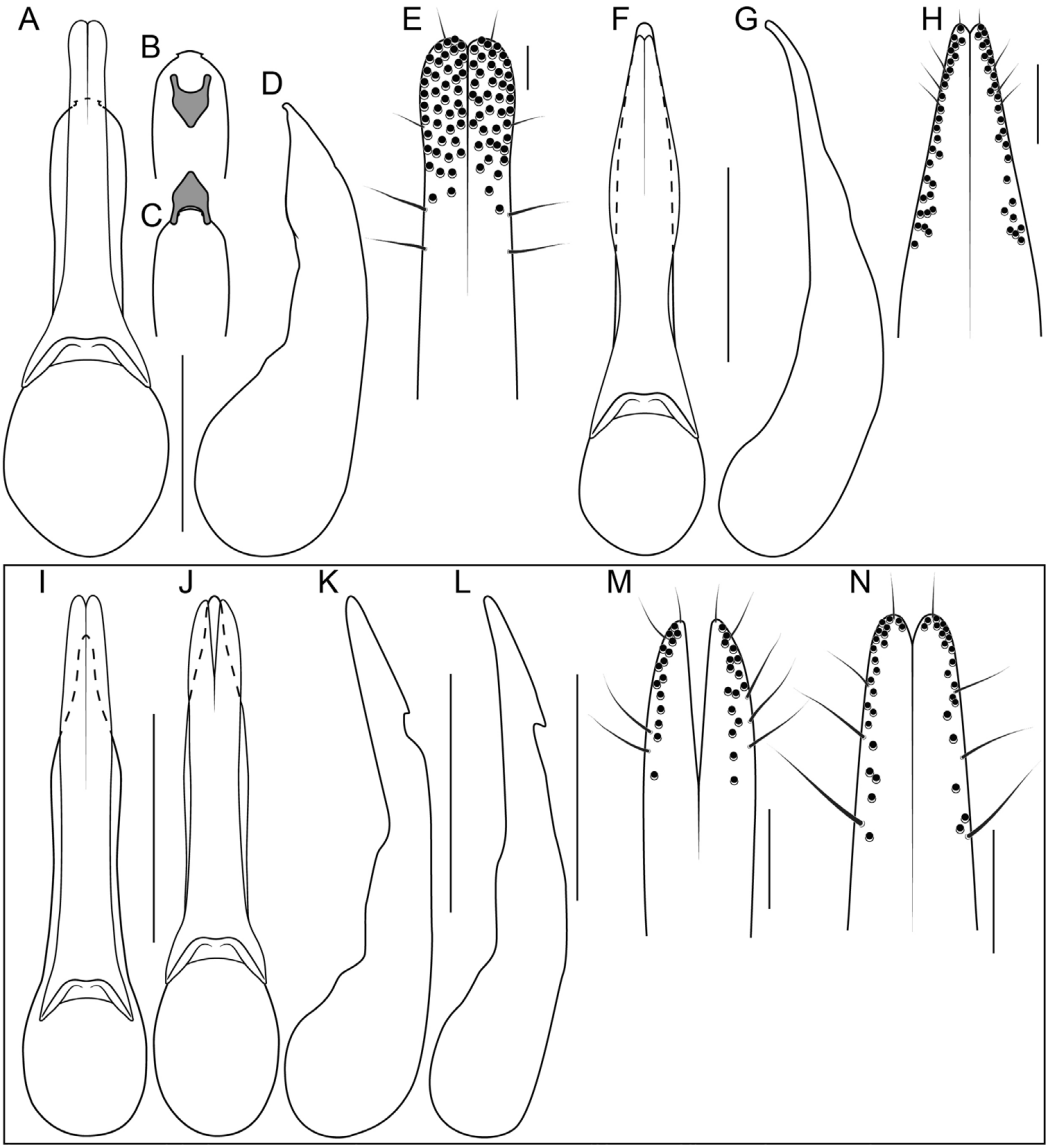
Aedeagus, parameral view (**A, F, I, J**), apex with internal sclerites (**B, C**). Aedeagus, lateral view (**D, G, K, L**). Underside of parameral lobe, with peg setae (**E, H, M, N**). *Bolitogyrus viridescens* Brunke (**A–E**); *Bolitogyrus apicofasciatus* Brunke (**F–H**); *Bolitogyrus bullatus* (**I–N**). Scale bar = 0.5 mm (**A–D, F, G, I–L**), 0.1 mm (**E, H, M, N**).

**Figure 19.**
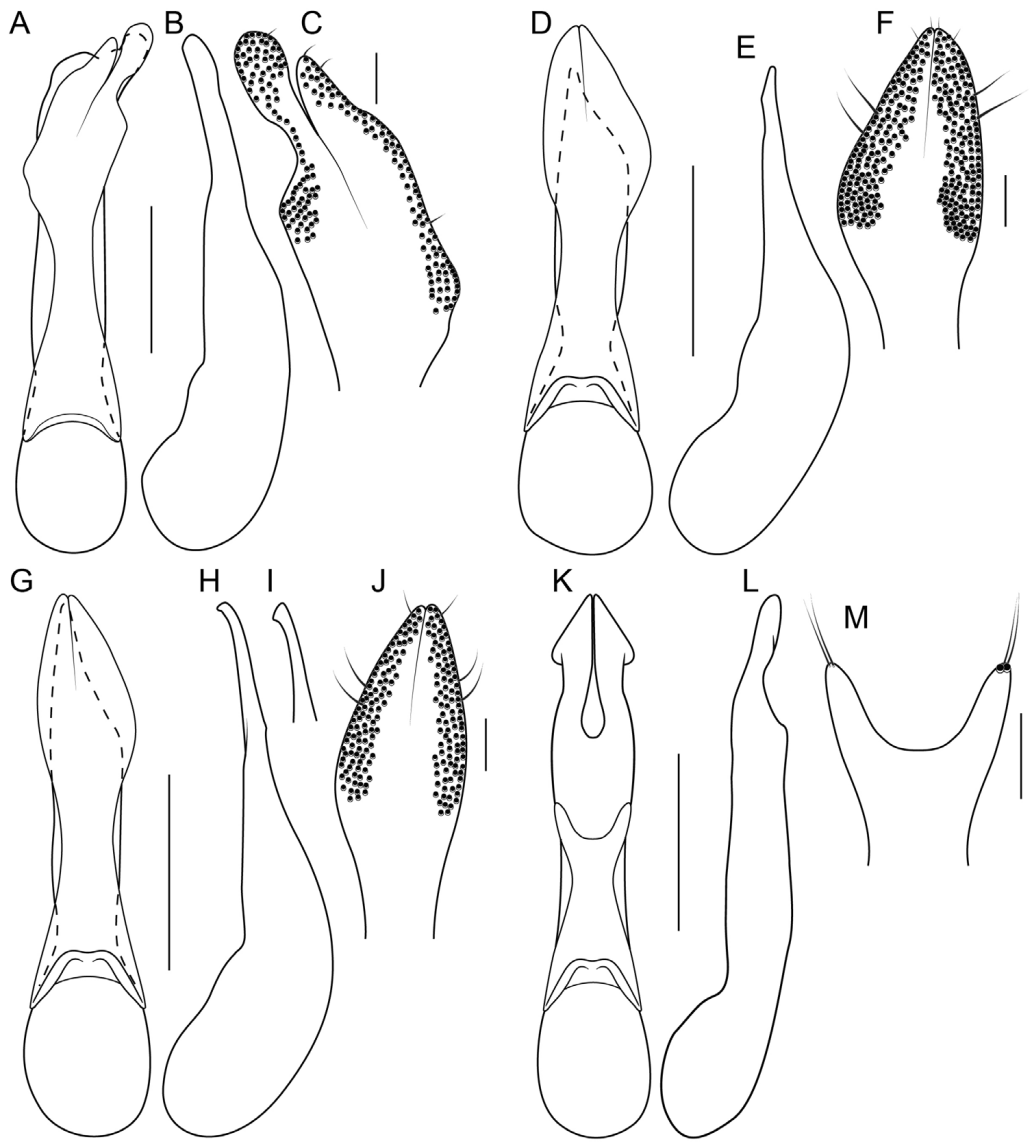
Aedeagus, parameral view (**A, D, G, K**). Aedeagus, lateral view (**B, E, H, I, L**). Underside of parameral lobe, with peg setae (**C, F, J, M**). *Bolitogyrus ashei* Brunke (**A–C**); *Bolitogyrus tortifolius* Brunke (**D–F**); *Bolitogyrus pseudotortifolius* (**G–J**); *Bolitogyrus inexspectatus* Brunke (**K–M**). Scale bar = 0.5 mm (**A, B, D, E, G, H, I, K, L**), 0.1 mm (**C, F, J, M**).

**Figure 20.**
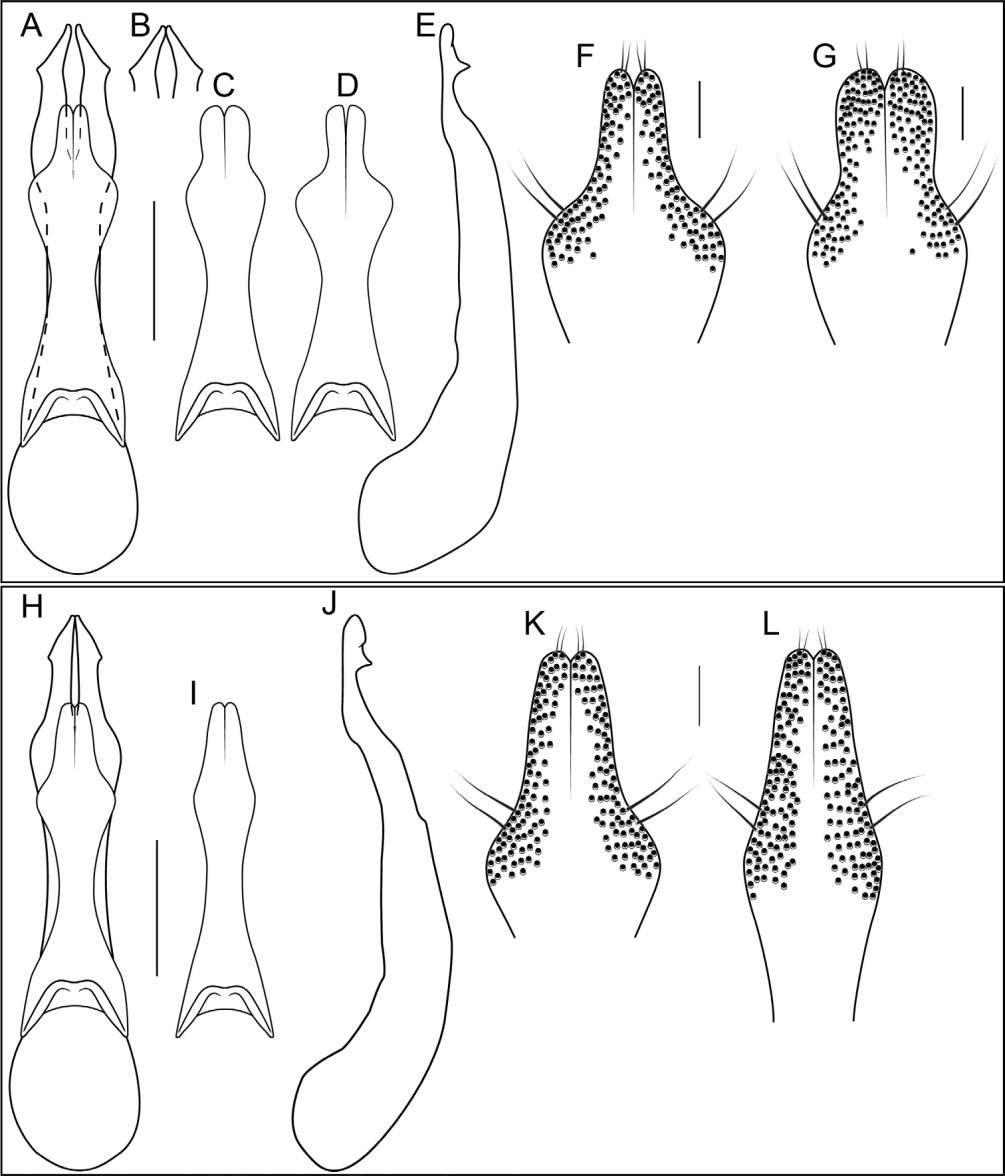
Aedeagus, parameral view (**A, H**), apex (**B**). Aedeagus, lateral view (**E, J**). Paramere, parameral view (**C, D, I**). Underside of parameral lobe, with peg setae (**F, G, K, L**). *Bolitogyrus divisus* Brunke (**A–G**); *Bolitogyrus falini* Brunke (**H–L**). Scale bar = 0.5 mm (**A–E, H–J**), 0.1 mm (**F, G, K, L**).

**Figure 21.**
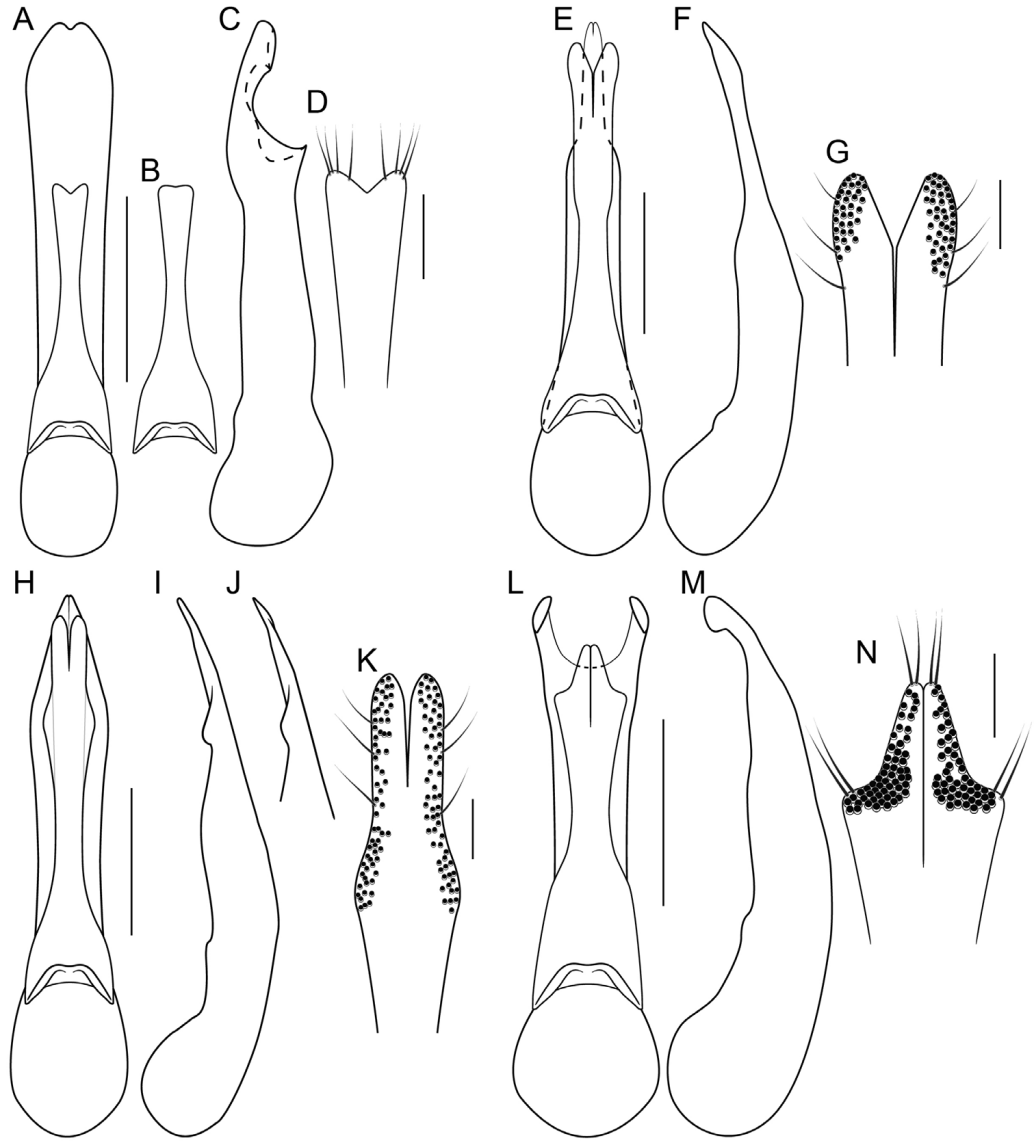
Aedeagus, parameral view (**A, E, H, L**). Aedeagus, lateral view (**C, F, I, J, M**). Paramere, parameral view (**B**). Underside of parameral lobe, with peg setae (**D, G, K, N**). *Bolitogyrus cornutus* Brunke (**A–D**); *Bolitogyrus brevistellus* Brunke (**E–G**); *Bolitogyrus bufo* Brunke (**H–K**); *Bolitogyrus cheungi* Brunke (**L–N**). Scale bar = 0.5 mm (**A–C, E, F, H–J, L, M**), 0.1 mm (**D, G, K, N**).

**Figure 22.**
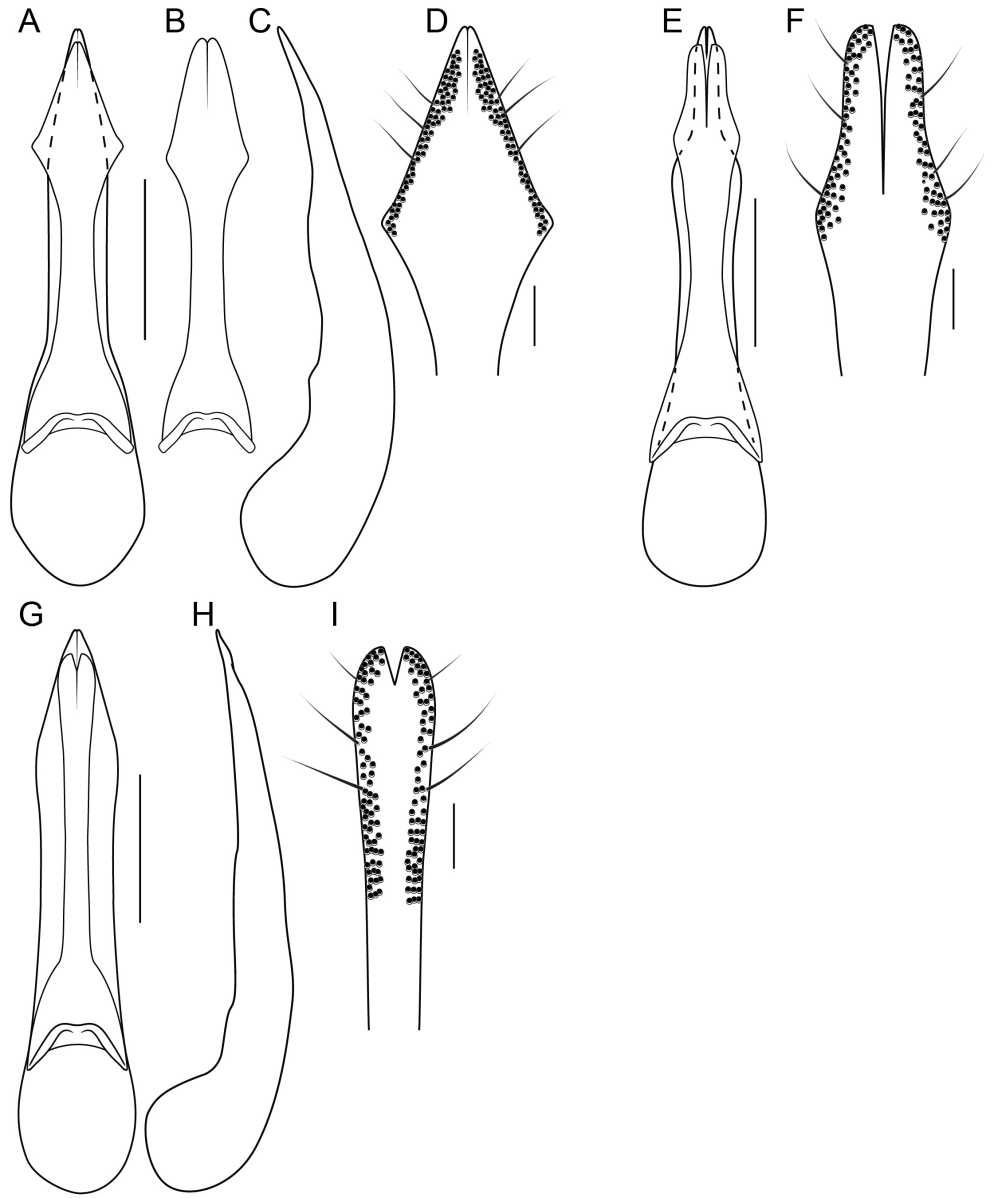
Aedeagus, parameral view (**A, E, G**). Aedeagus, lateral view (**C, H**). Paramere, parameral view (**B**). Underside of parameral lobe, with peg setae (D, F, **I**). *Bolitogyrus gracilis* Brunke (**A–D**); *Bolitogyrus longistellus* Brunke (**E–F**), median lobe in lateral view similar to *Bolitogyrus gracilis*; *Bolitogyrus thomasi* Brunke (**G–I**). Scale bar = 0.5 mm (**A–C, E, G, H**), 0.1 mm (**D, F, I**).

**Figure 23.**
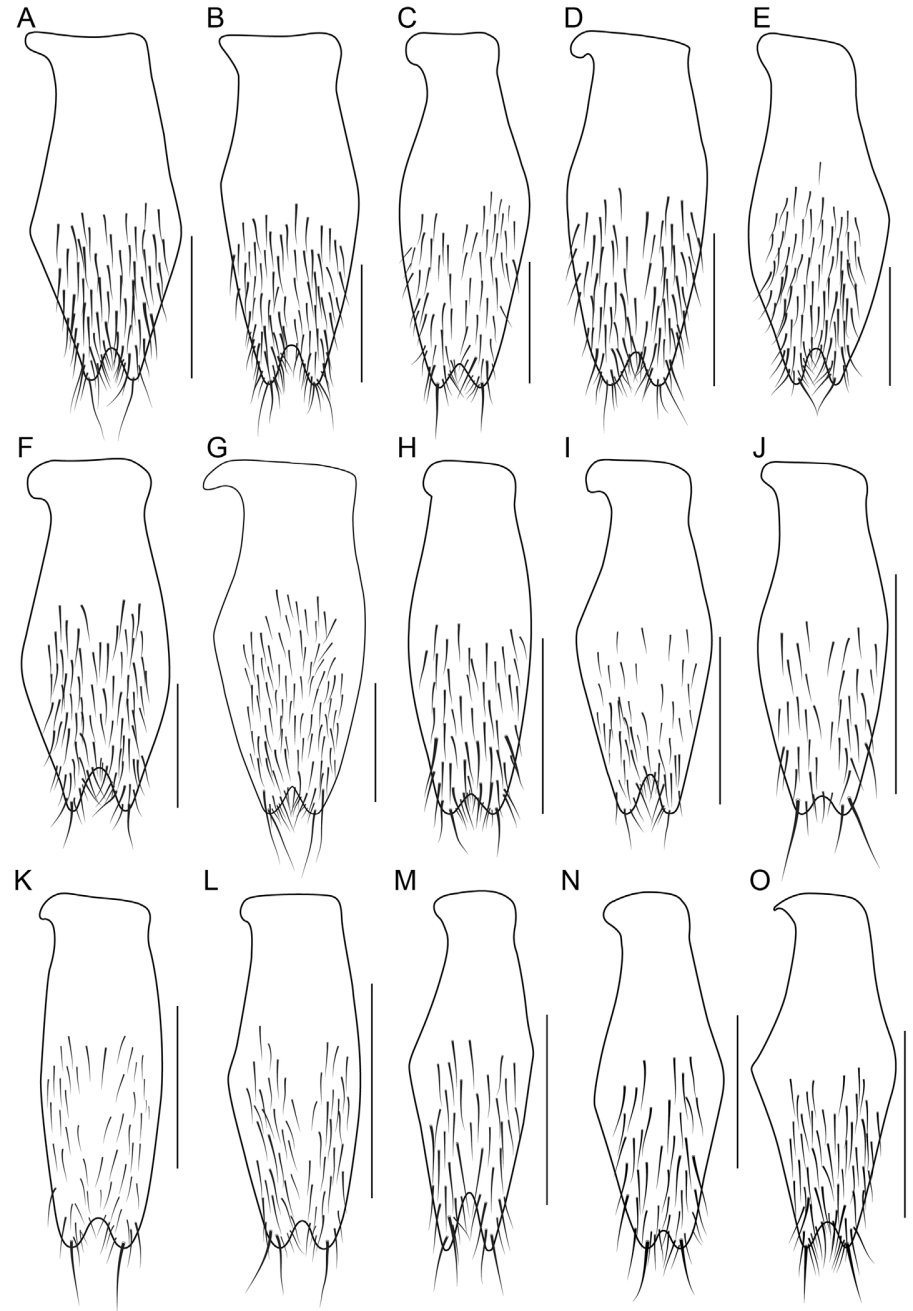
Male sternite IX. *Bolitogyrus buphthalmus* (Erichson) (**A**); *Bolitogyrus costaricensis* Wendeler (**B**); *Bolitogyrus pulchrus* Brunke (**C**); *Bolitogyrus sallei* (Kraatz) (**D**); *Bolitogyrus salvini* (Sharp) (**E–F**), lectotype (**E**), ‘morphotype III’ (**F**); *Bolitogyrus bechyneorum* (Scheerpeltz) (**G**); *Bolitogyrus newtoni* Brunke (**H**); *Bolitogyrus marquezi* Brunke (**I**); *Bolitogyrus strigifrons* (Wendeler) (**J**); *Bolitogyrus silex* Brunke (**K**); *Bolitogyrus viridescens* Brunke (**L**); *Bolitogyrus bullatus* (Sharp) (**M**); *Bolitogyrus ashei* Brunke (**N**); *Bolitogyrus apicofasciatus* Brunke (**O**). Scale bar = 0.5 mm.

**Figure 24.**
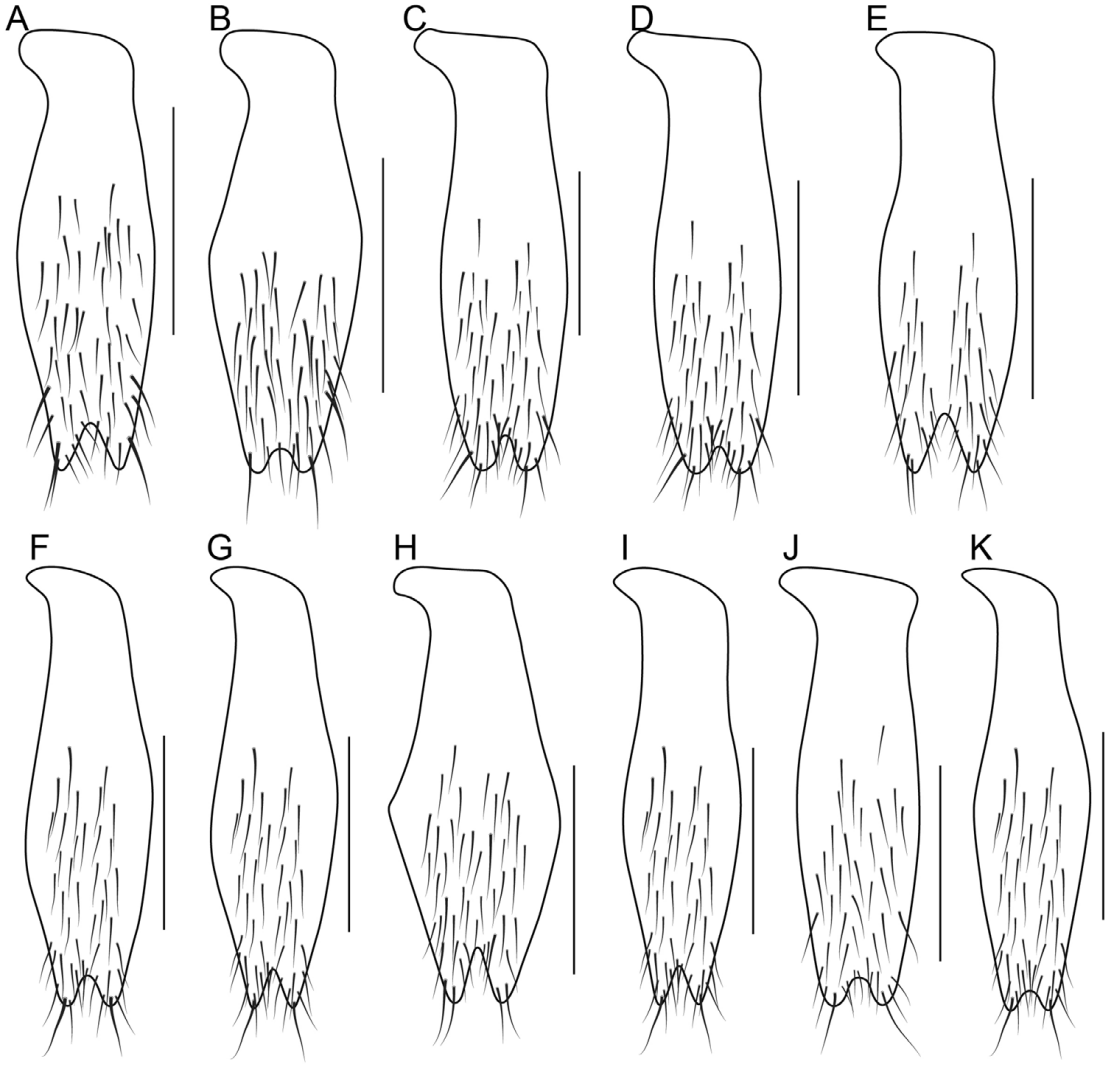
Male sternite IX. *Bolitogyrus tortifolius* Brunke (**A**); *Bolitogyrus pseudotortifolius* Brunke (**B**); *Bolitogyrus divisus* Brunke (same as *Bolitogyrus falini* Brunke) (**C**); *Bolitogyrus inexspectatus* Brunke (**D**); *Bolitogyrus cornutus* Brunke (**E**); *Bolitogyrus brevistellus* Brunke (**F**); *Bolitogyrus bufo* Brunke (**G**); *Bolitogyrus cheungi* Brunke (**H**); *Bolitogyrus gracilis* Brunke (**I**); *Bolitogyrus longistellus* Brunke (**J**); *Bolitogyrus thomasi* Brunke (**K**). Scale bar = 0.5 mm.

**Figure 25.**
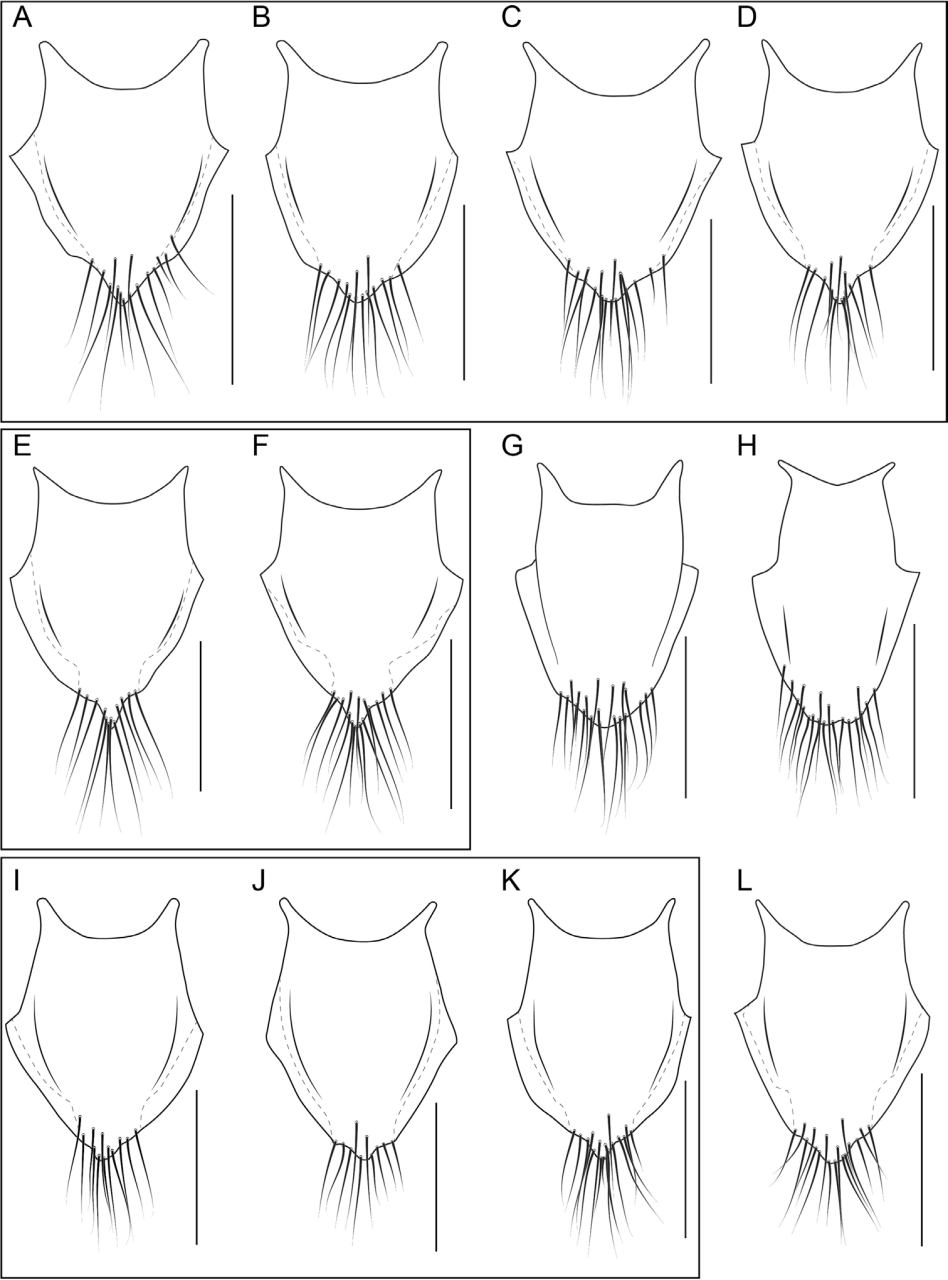
Female tergite X. *Bolitogyrus costaricensis* (Wendeler) (**A–D**), holotype (**A**); holotype of *Bolitogyrus nevermanni* (Scheerpeltz) (**B**); *Bolitogyrus buphthalmus* (Erichson) (**E–F**), paralectotype of *Bolitogyrus cyanescens* (Sharp) (**E**); *Bolitogyrus bechyneorum* (Scheerpeltz) (**G**); *Bolitogyrus newtoni* Brunke (**H**); *Bolitogyrus erythrurus* (Kraatz) (**I–K**), syntype (**I**); *Bolitogyrus sallei* (Kraatz) (**L**). Scale bar = 0.5 mm.

**Figure 26.**
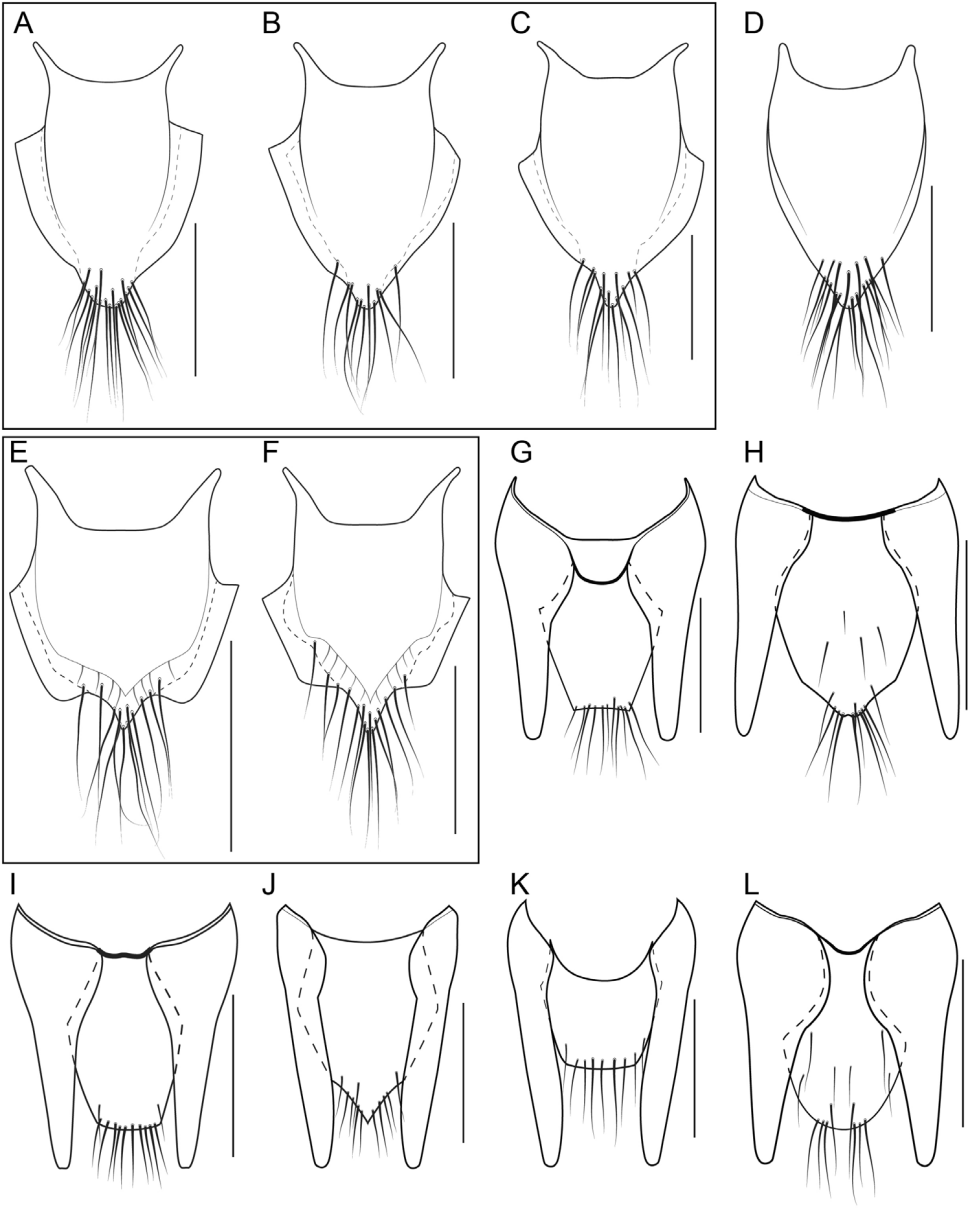
Female tergite X. *Bolitogyrus salvini* (Sharp) (**A–C**), paralectotype (**A**), ‘morphotype II’ (**B**), ‘morphotype III’ (**C**); *Bolitogyrus pulchrus* Brunke (**D**); *Bolitogyrus fulgidus* (Sharp) (**E–F**), holotype (**E**), ‘dark Pacific morphotype’ (**F**); *Bolitogyrus marquezi* Brunke (**G**); *Bolitogyrus silex* Brunke (**H**); *Bolitogyrus viridescens* Brunke (**I**); *Bolitogyrus bullatus* (Sharp) (**J**); *Bolitogyrus ashei* Brunke (**K**); *Bolitogyrus apicofasciatus* Brunke (**L**). Scale bar = 0.5 mm.

**Figure 27.**
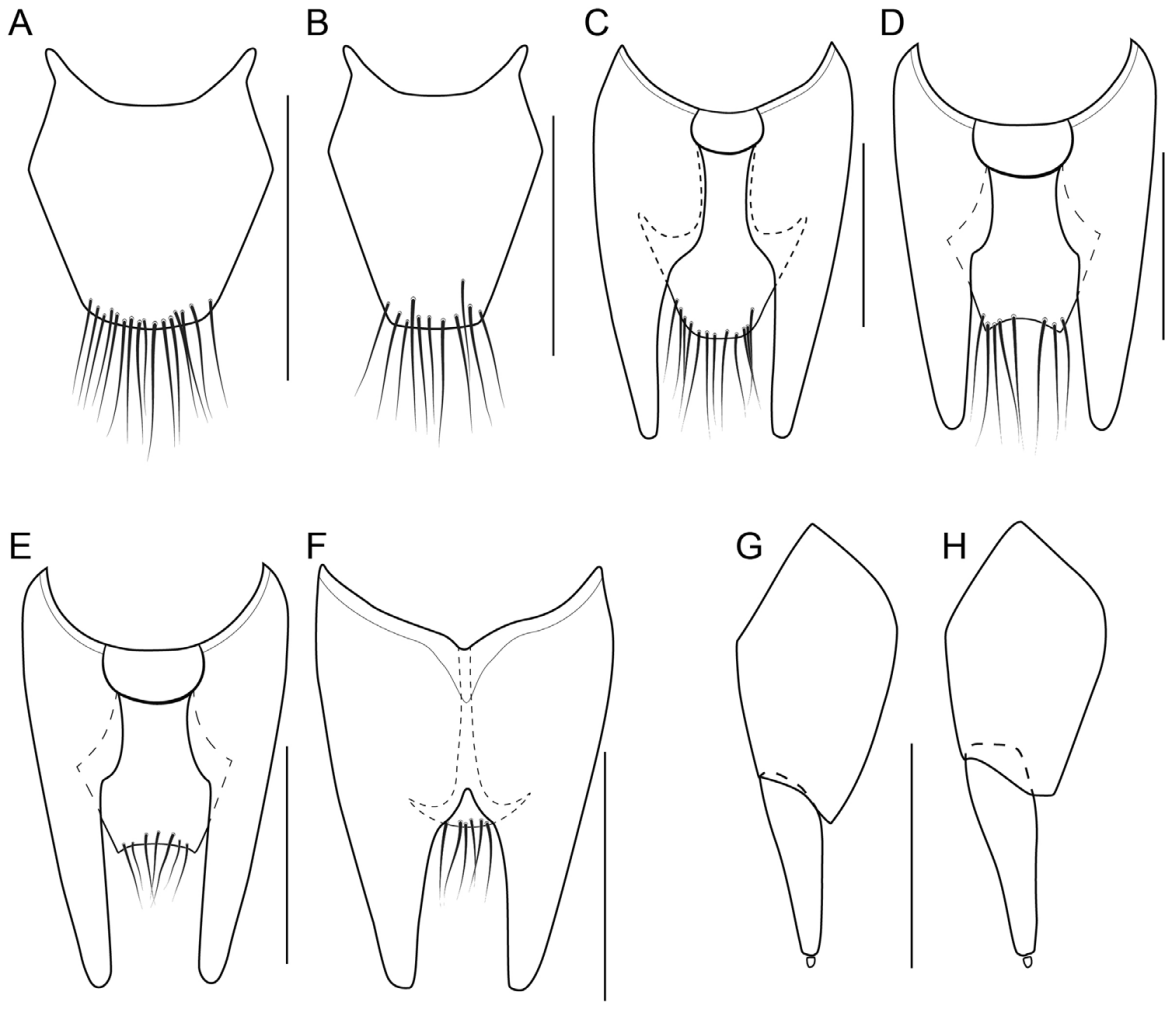
Female tergite X: *Bolitogyrus tortifolius* Brunke (**A**); *Bolitogyrus pseudotortifolius* Brunke (**B**); *Bolitogyrus divisus* Brunke (same as *Bolitogyrus falini* Brunke) (**C**); *Bolitogyrus cornutus* Brunke (**D**); *Bolitogyrus bufo* Brunke (same as *Bolitogyrus gracilis*) (**E**); *Bolitogyrus cheungi* Brunke (**F**). Female gonocoxite (setae omitted): *Bolitogyrus salvini* (Sharp) (**G**); *Bolitogyrus fulgidus* (Sharp) (**H**). Scale bar = 0.5 mm

**Figure 29.**
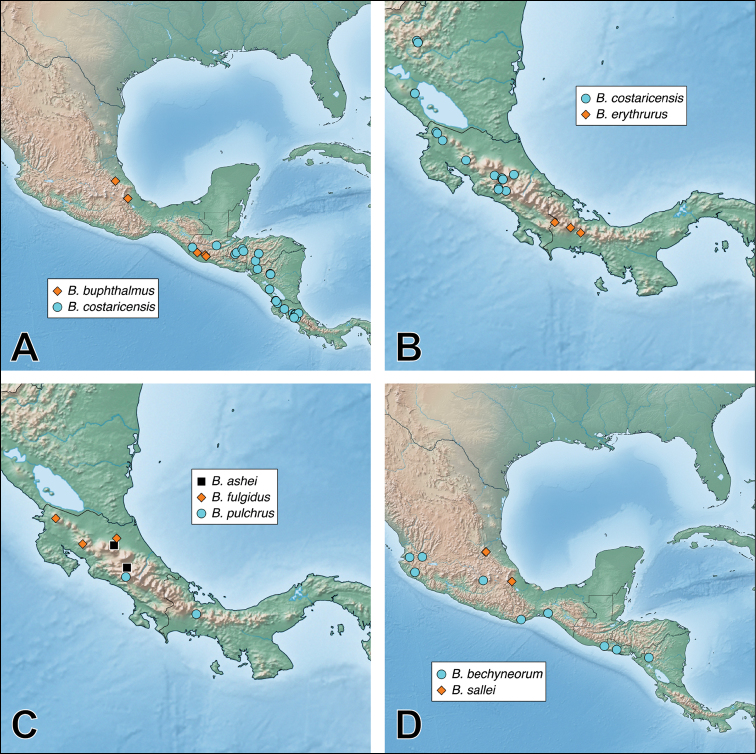
Distribution of: *Bolitogyrus buphthalmus* (Erichson) and *Bolitogyrus costaricensis* (Wendeler) (**A**); *Bolitogyrus costaricensis* and *Bolitogyrus erythrurus* (Kraatz) (**B**); *Bolitogyrus ashei* Brunke, *Bolitogyrus fulgidus* (Sharp) and *Bolitogyrus pulchrus* Brunke (**C**); *Bolitogyrus bechyneorum* (Scheerpeltz) and *Bolitogyrus sallei* (Kraatz) (**D**).

**Figure 30.**
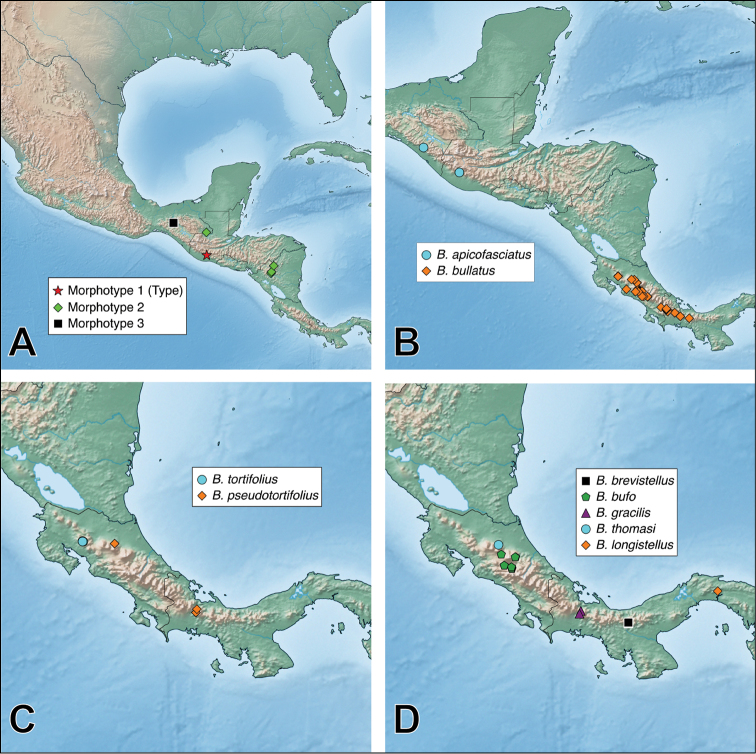
Distribution of: *Bolitogyrus salvini* (Sharp) morphotypes (**A**); *Bolitogyrus apicofasciatus* Brunke and *Bolitogyrus bullatus* (Sharp) (**B**); *Bolitogyrus pseudotortifolius* Brunke and *Bolitogyrus tortifolius* Brunke (**C**); *Bolitogyrus brevistellus* Brunke, *Bolitogyrus bufo* Brunke, *Bolitogyrus gracilis* Brunke, *Bolitogyrus longistellus* Brunke and *Bolitogyrus thomasi* Brunke (**D**).

Similar to *Bolitogyrus buphthalmus* and differing only in the following: head and pronotum black, glossy and with dark green metallic reflection; elytra black with bronze to dark green-bronze metallic reflection; antennomeres VI–X hardly to distinctly darker than I–V, apical antennomere not paler than preceding segments; coxa dark in both sexes, contrasting with pale femur, very apex of mesofemur with diffuse dark band (female) or without band (male), metafemur with dark apical band; all tibiae slightly (male) to distinctly (female) darkened; forebody distinctly shorter than in *Bolitogyrus buphthalmus*; head slightly less transverse than in *Bolitogyrus buphthalmus*; median frontal impression barely perceivable or absent; pronotum with pair of depressions but without distinct protuberance in lateral view; surface of elytra only weakly uneven; suture-to-pronotum length ratio greater than in *Bolitogyrus buphthalmus*; abdominal sternites with relatively coarse transverse microsculpture, interspaces wider than lines; median lobe in lateral view sinuate and projected ventrad (Fig. 17B); apical portion of median lobe in parameral view not constricted at base (Fig. 17A); paramere only weakly divided near apex and flexed over the apex of the median lobe; peg setae in distinctive arrangement (Fig. 17C); male sternite IX narrower and with emargination shallower and wider, base distinctly less asymmetrical (Fig. 23H); female tergite X much narrower, with raised disc only weakly converging apicad, with apex slightly emarginate and not produced (Fig. 25H).

###### Distribution.

**Figure 31.**
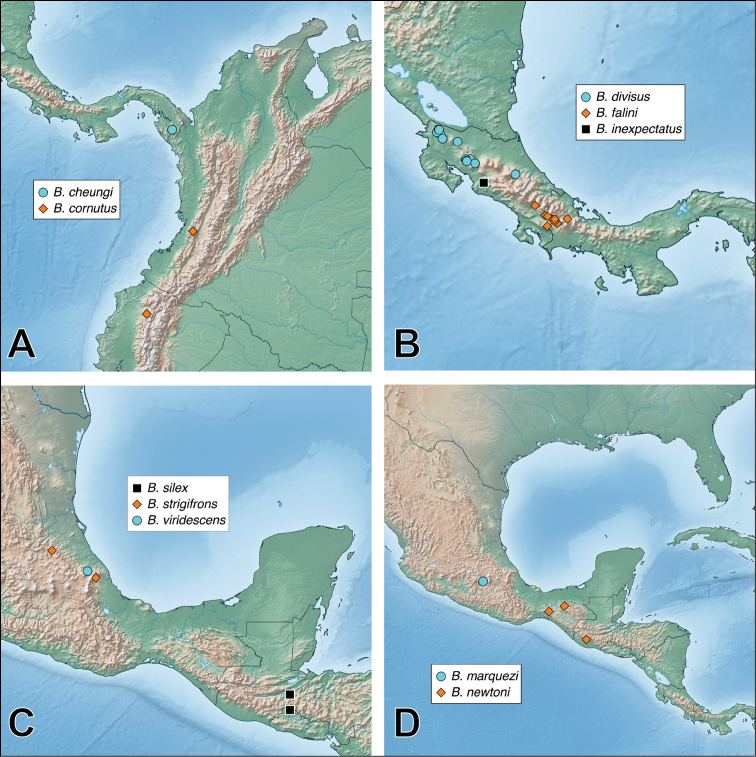
Distribution of: *Bolitogyrus cheungi* Brunke and *Bolitogyrus cornutus* Brunke (**A**); *Bolitogyrus divisus* Brunke, *Bolitogyrus falini* Brunke, *Bolitogyrus inexspectatus* Brunke (**B**); *Bolitogyrus silex* Brunke, *Bolitogyrus strigifrons* (Wendeler) and *Bolitogyrus viridescens* Brunke (**C**); *Bolitogyrus marquezi* Brunke and *Bolitogyrus newtoni* Brunke (**D**).

**Figure 32.**
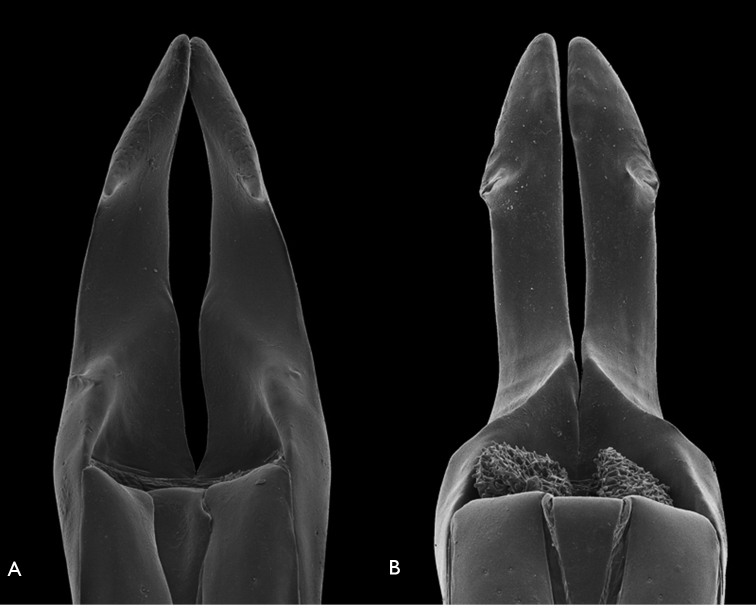
rSEM of apical portion of the median lobe: *Bolitogyrus divisus* Brunke (**A:** left) and *Bolitogyrus falini* (**B:** right). To view these figures, Adobe Acrobat version 8 or higher is required.

Figure 31D. *Bolitogyrus newtoni* is known from northern Chiapas in Mexico and from Suchitepéquez state in southern Guatemala.

###### Bionomics.

Specimens were collected from the forest floor and from a cluster of mushrooms on a log, at 1550–1676 m. The holotype was collected in a dung trap but an association of this species with the dung community is unlikely. Specimens were collected in June and August.

###### Etymology.

The first author would like to dedicate this species to Dr. Alfred (Al) Newton (FMNH), a friend and mentor. While collecting the holotype of *Bolitogyrus newtoni*, Al was sampling for *Platydracus* (Staphylininae: Staphylinina), a genus that he is currently revising and the subject of our first collaborative project.

###### Comments.

*Bolitogyrus newtoni* is most similar to and sympatric with *Bolitogyrus buphthalmus* and the dark individuals of *Bolitogyrus salvini* (morphotype III). However, it may be distinguished by the apical antennomere which is not paler than the previous segments and the sparse, microsculpture on the ventral abdomen.

### Undescribed species of *Bolitogyrus*

#### Undescribed species #1

**PANAMA:** *Darien*, Cana Biological Station, 7°45'18"N, 77°41'6"W, 500-550 m, ex. fungusy log, 3.VI.1996, J. Ashe and R. Brooks, PAN1AB96 009, SM0042289, 1 ♀ (SEMC).

This single female from eastern Panama is similar to *Bolitogyrus newtoni* in habitus and small size, and also lacks the median frontal impression. Unlike in *Bolitogyrus newtoni*, the forebody has a violet-blue metallic reflection, the procoxae are pale, the hind legs are entirely dark brown, the ventral abdominal microsculpture is extremely fine and the form of female tergite X is different. It is almost certainly belongs to an undescribed species but without characters of the male aedeagus, describing a species from such a poorly collected region may create taxonomic problems in the future.

#### Undescribed species #2

**MEXICO:** *Jalisco*: Tequila, Volcán de Tequila, bosque mesófilo [=cloud forest], ex. bajo musgo [=under moss] 2350 m, 20.II.1994, J. L. Navarrete col., 1 ♀ (CZUJ). *Morelos*: Cuernavaca, Ocuilan, VII.1992, A. Pérez, 1 ♂ [missing aedeagus] (CZUJ).

The male and female specimens, each from Mexico, are similar to *Bolitogyrus bechyneorum* in habitus, and to *Bolitogyrus newtoni* as they lack a median frontal impression. Unlike these two species, the elytra and abdomen, except for the apex of the latter, are reddish. The male is unfortunately missing the aedeagus and this species cannot be described.

## Supplementary Material

XML Treatment for Bolitogyrus

XML Treatment for Bolitogyrus buphthalmus

XML Treatment for Cyrtothorax sallei

XML Treatment for Cyrtothorax cyanescens

XML Treatment for Bolitogyrus costaricensis

XML Treatment for Cyrtothorax nevermanni

XML Treatment for Bolitogyrus erythrurus

XML Treatment for Bolitogyrus fulgidus

XML Treatment for Bolitogyrus pulchrus

XML Treatment for Bolitogyrus sallei

XML Treatment for Bolitogyrus salvini

XML Treatment for Bolitogyrus bullatus

XML Treatment for Bolitogyrus apicofasciatus

XML Treatment for Bolitogyrus ashei

XML Treatment for Bolitogyrus tortifolius

XML Treatment for Bolitogyrus pseudotortifolius

XML Treatment for Bolitogyrus cornutus

XML Treatment for Bolitogyrus brevistellus

XML Treatment for Bolitogyrus bufo

XML Treatment for Bolitogyrus cheungi

XML Treatment for Bolitogyrus gracilis

XML Treatment for Bolitogyrus longistellus

XML Treatment for Bolitogyrus thomasi

XML Treatment for Bolitogyrus divisus

XML Treatment for Bolitogyrus falini

XML Treatment for Bolitogyrus inexspectatus

XML Treatment for Bolitogyrus strigifrons

XML Treatment for Bolitogyrus silex

XML Treatment for Bolitogyrus viridescens

XML Treatment for Bolitogyrus bechyneorum

XML Treatment for Bolitogyrus marquezi

XML Treatment for Bolitogyrus newtoni

## Figures and Tables

**Figure 28.**
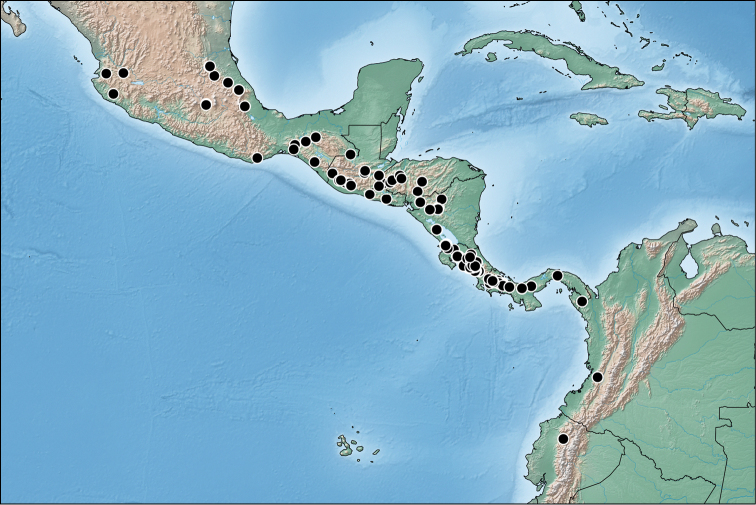
Distribution of *Bolitogyrus* Chevrolat in the Neotropical Region.

## References

[B1] Alcock J (1991) Adaptive Mate-Guarding by Males of *Ontholestes cingulatus* (Coleoptera: Staphylinidae). Journal of Insect Behavior 4: 763–771. doi: 10.1007/BF01052230

[B2] Bernhauer M (1915) Neue Staphyliniden der indo-malaiischen Fauna, insbesondere der Sunda-Insel Borneo. Verhandlungen der k k zoologisch-botanischen Gesellschaft in Wien 65: 134–158

[B3] Blackwelder RE (1952) The generic names of the beetle family Staphylinidae, with an essay on genotypy. United States National Museum Bulletin 200: 1–483

[B4] Brunke A Solodovnikov A (2013) *Alesiella* gen.n. and a newly discovered relict lineage of Staphylinini (Coleoptera: Staphylinidae). Systematic Entomology 38: 689–707. doi: 10.1111/syen.12021

[B5] Cameron M (1932) The fauna of British India including Ceylon and Burma. Coleoptera. Staphylinidae. Talyor and Francis, London, 443 pp

[B6] Cameron M (1937) Fauna Javanica. The Staphylinidae collected by Mr. F. C. Drescher. Part II. Tijdschrift voor Entomologie 80: 1–37

[B7] Cameron M (1942) New species of Staphylinidae (Col.) from Borneo. The Entomologist’s Monthly Magazine 78: 136–139

[B8] Chatzimanolis S Cohen IM Schomann AS Solodovnikov A (2010) Molecular phylogeny of the mega-diverse rove beetle tribe Staphylinini (Insecta, Coleoptera, Staphylinidae). Zoologica Scripta 39: 436–449. doi: 10.1111/j.1463-6409.2010.00438.x

[B9] Chevrolat A (1842) *Bolitogyrus*. In: D’Orbigny C (Ed) Dictionnaire Universel d’Histoire Naturelle. Bureau Principal de Éditeurs, Paris, 2, 641

[B10] Cheung DK-B Brunke A Akkari N Souza C Pape T (2013) Rotational Scanning Electron Micrographs (rSEM): A novel and accessible tool to visualize and communicate complex morphology. ZooKeys 328: 47–57. doi: 10.3897/zookeys.328.5768 24146547 10.3897/zookeys.328.5768 PMC3800821

[B11] Dejean PFMA (1836) Catalogue de la collection de Coléoptères de M. le Comte Dejean. Méquignon-Marvis Père et Fils., Paris, 503 pp

[B12] Emlen D (1997) Alternative reproductive tactics and male-dimorphism in the horned beetle *Onthophagus acuminatus* (Coleoptera: Scarabaeidae). Behavioral Ecology and Sociobiology 41: 335–341. doi: 10.1007/s002650050393

[B13] Erichson WF (1840) Genera et species Staphylinorum insectorum coleopterorum familiae. FH Morin, Berlin, 400 pp

[B14] Fauvel A (1878) Révision du genre *Cyrtothorax*. Bulletin de la Société Linnéenne de Normandie 3: 163–166

[B15] Hammond PM (1984) An annotated checklist of Staphylinidae (Insecta: Coleoptera) recorded from Borneo. The Sarawak Museum Journal 33: 187–218

[B16] Hayashi Y (1991) Studies on Staphylinidae from Japan. III. The Entomological Review of Japan 46: 179–185

[B17] Herman L (2001a) Nomenclatural changes in the Staphylinidae (Insecta: Coleoptera). Bulletin of the American Museum of Natural History No. 264: 1–83

[B18] Herman L (2001b) Catalog of the Staphylinidae (Insecta, Coleoptera): 1758 to the end of the second millennium. Bulletin of the American Museum of Natural History 265: 1–4218

[B19] Horn W Kahle I Friese G Gaedike R (1990a) Collectiones entomologicae, Ein Kompendium über den Verbleib entomologischer Sammlungen der Welt bis 1960, Teil I: A bis K. Akademie der Landwirtschaftswissenschaften der Deutschen Demokratischen Republik, Berlin, 220 pp

[B20] Horn W Kahle I Friese G Gaedike R (1990b) Collectiones entomologicae, Ein Kompendium über den Verbleib entomologischer Sammlungen der Welt bis 1960, Teil II: L bis Z. Akademie der Landwirtschaftswissenschaften der Deutschen Demokratischen Republik, Berlin, 221–573

[B21] Hu J-Y Liu T-T Li L-Z (2011) New and Little-Known Species of the Genus *Bolitogyrus* Chevrolat from China (Coleoptera: Staphylinidae: Staphylininae). Journal of the Kansas Entomological Society 84: 58–63. doi: 10.2317/JKES101003.1

[B22] Kraatz G (1858) Einige neue und ausgezeichnete Staphylinen-Gattungen. Berliner Entomologische Zeitschrift 2: 361–368. doi: 10.1002/mmnd.18580020310

[B23] Lingafelter S Anderson R Timm R Falin Z Jameson M Newton AF Ball G Ahn KJ Leschen R (2006) In memoriam, James Stephen “Steve” Ashe (1947–2005). [obituary]. The Coleopterists Bulletin 60: 1–12. doi: 10.1649/889.1

[B24] Márquez J (2006) Primeros registros estatales y datos de distribución geográfica de especies Mexicanas de Staphylinidae (Coleoptera). Boletin Sociedad Entomológica Aragonesa 38: 181–198

[B25] Navarrete-Heredia JL Newton AF Thayer MK Ashe JS Chandler DS (2002) Guía ilustrada para los géneros de Staphylinidae (Coleoptera) de México. Illustrated guide to the genera of Staphylinidae (Coleoptera) of Mexico Universidad de Guadalajara y Conabio, México, 401 pp

[B26] Rougemont G (2001) A new species in each of the quediine genera *Bolitogyrus* Chevrolat and *Indoquedius* Cameron (Col., Staphylinidae) from India. Entomologist’s Monthly Magazine 137: 111–114

[B27] Scheerpeltz O (1962) Eine neue Gattung und Art der Tribus Quediini (Col. Staphylinidae). Annalen des Naturhistorischen Museums in Wien 65: 259–272

[B28] Scheerpeltz O (1974) Studien an den Arten der Gattung *Cyrtothorax* Kraatz mit Beschreibung neuer Arten sowie einer Dichotomik aller bis heute bekannt gewordenen Arten dieser Gattung (Coleoptera, Staphylinindae, Staphylininae, Quediini). Reichenbachia 15: 175–192

[B29] Selander RB Vaurie P (1962) A gazetter to accompany the ‘Insecta’ volumes of the ‘Biologia Centrali-Americana’. American Museum Novitates 2099: 1–70

[B30] Sharp D (1884) Staphylinidae Biologia Centrali-Americana Insecta Coleoptera 1 (2). Taylor and Francis, London, 313–392

[B31] Shibata Y (1979) New or little-known Staphylinidae (Coleoptera). The Entomological Review of Japan 33: 19–29

[B32] Shibata Y (1986) A list of genera and species new to Taiwan and new data on distribution of the Staphylinidae discovered from Taiwan since 1973 (Insecta: Coleoptera). Annual Bulletin of the Nichidai Sanko 24: 109–128

[B33] Solodovnikov A Schomann A (2009) Revised systematics and biogeography of ‘Quediina’ of sub-Saharan Africa: new phylogenetic insights into the rove beetle tribe Staphylinini (Coleoptera: Staphylinidae). Systematic Entomology 34: 443–466. doi: 10.1111/j.1365-3113.2008.00468.x

[B34] Solodovnikov A Yue Y Tarasov S Ren D (2013) Extinct and extant rove beetles meet in the matrix: Early Cretaceous fossils shed light on the evolution of a hyperdiverse insect lineage (Coleoptera: Staphylinidae: Staphylininae). Cladistics 29: 360–403. doi: 10.1111/j.1096-0031.2012.00433.x 10.1111/j.1096-0031.2012.00433.x 34836452

[B35] Smetana A (1971) Revision of the tribe Quediini of North America north of Mexico (Coleoptera: Staphylinidae). Memoirs of the Entomological Society of Canada 79: 1–303. doi: 10.4039/entm10379fv

[B36] Smetana A (1988) Revision of the tribes Quediini and Atanygnathini. Part II. The Himalayan region (Coleoptera: Staphylinidae). Quaestiones Entomologicae 24(2): 163–464

[B37] Smetana A (1995) Revision of the tribes Quediini and Tanygnathinini. Part III. Taiwan. (Coleoptera: Staphylinidae). National Museum of Natural Science (Special Publication) 6: 1–145

[B38] Smetana A (2000) Contributions to the knowledge of the Quediina (Coleoptera, Staphylinidae, Staphylinini) of China. Elytra 28: 327–330

[B39] Smetana A Zheng F (2000) Contributions to the knowledge of the Quediina (Coleoptera, Staphylinidae, Staphylinini) of China. Part 17. Genus *Bolitogyrus* Chevrolat, 1842. Section 1. Elytra 28: 55–64

[B40] Wendeler H (1927) Neue exotische Staphyliniden (Col.). Neue Beiträge zur systematischen Insektenkunde 4: 2–9

[B41] Wendeler H (1928) Neue exotische Staphyliniden. Neue Beiträge zru systematischen Insektenkunde 4: 32–35

[B42] Yuan X Zhao M-J Li L-Z Hayashi Y (2007) Contributions to the knowledge of the genus *Bolitogyrus* (Coleoptera: Staphylinidae) of China. Entomological Review of Japan 62: 145–155

[B43] Zheng F (1988) A new species of the genus *Cyrtothorax* Kraatz from Sichuan Province (Coleoptera: Staphylinidae, Quediinae). Acta Entomologica Sinica 31: 306–308

